# Unraveling Structural and Anticancer Properties of Pyridine-Oxadiazole Derivatives: Single-Crystal XRD, Hirshfeld Analysis, and Cytotoxicity against A549 Cells

**DOI:** 10.1021/acsomega.5c02152

**Published:** 2025-06-01

**Authors:** Yogeesha N. Nayak, Deepika Dwarakanath, Keerthana Suresh Kizhakkanoodan, Rajeev K. Sinha, K. Sreedhara Ranganath Pai, Bharath Raja Guru, Santosh L. Gaonkar

**Affiliations:** † Department of Chemistry, 76793 Manipal Institute of Technology, Manipal Academy of Higher Education (MAHE), Manipal, Karnataka 576104, India; ‡ Department of Biotechnology, Manipal Institute of Technology (MIT), Manipal Academy of Higher Education (MAHE), Manipal, Karnataka 576104, India; § Department of Physics, 28698 Birla Institute of Technology Mesra, Ranchi 835215, India; ∥ Department of Pharmacology, 76808 Manipal College of Pharmaceutical Sciences, Manipal Academy of Higher Education (MAHE), Manipal, Karnataka 576104, India

## Abstract

A new series of pyridine-based 1,3,4-oxadiazole derivatives was synthesized and structurally characterized using FTIR, NMR, HRMS, and single-crystal X-ray diffraction. Hirshfeld surface analysis of the meta-methyl-substituted derivative revealed key intermolecular interactions. Cytotoxicity was evaluated against A549 lung cancer cells via MTT assay, where compound **5k** (3,5-dichloro substitution) showed the highest activity (IC_50_ = 6.99 ± 3.15 μM), comparable to 5-fluorouracil. Structure–activity relationship analysis indicated that meta-substituents enhance activity, while bulky or strongly electron-withdrawing groups reduce it. In silico studies demonstrated favorable ADME properties, and molecular docking with CDK2 revealed strong binding affinities. Molecular dynamics simulations confirmed the stability of the 5k–CDK2 complex over 100 ns. These findings suggest that pyridine–oxadiazole hybrids, particularly **5k**, are promising candidates for further development as anticancer agents.

## Graphical abstract

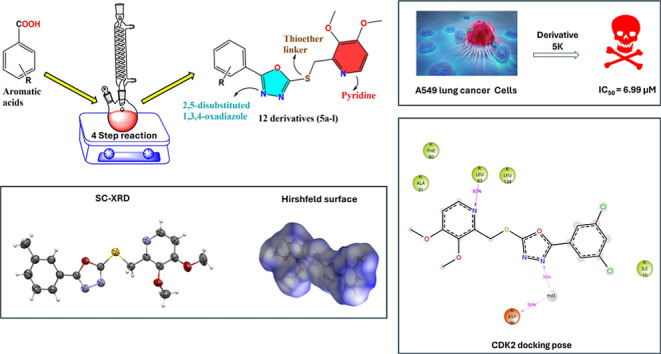

## 1 Introduction

Nitrogen-based heterocycles are fundamental to medicinal chemistry, forming the backbone of numerous bioactive molecules with therapeutic applications. The structural diversity and electronic properties of these systems enable precise tuning of molecular attributes such as solubility, stability, and target binding. Among heterocycles, five- and six-membered *N*-heterocycles hold particular significance due to their widespread presence in clinically relevant drugs. Their physicochemical properties contribute to enhanced bioavailability, metabolic stability, and biological activity, making them key components in the design of novel therapeutic agents, as exemplified by several five- and six-membered *N*-heterocycles featured in WHO essential medicines (Figure 1).

**Figure 1:**
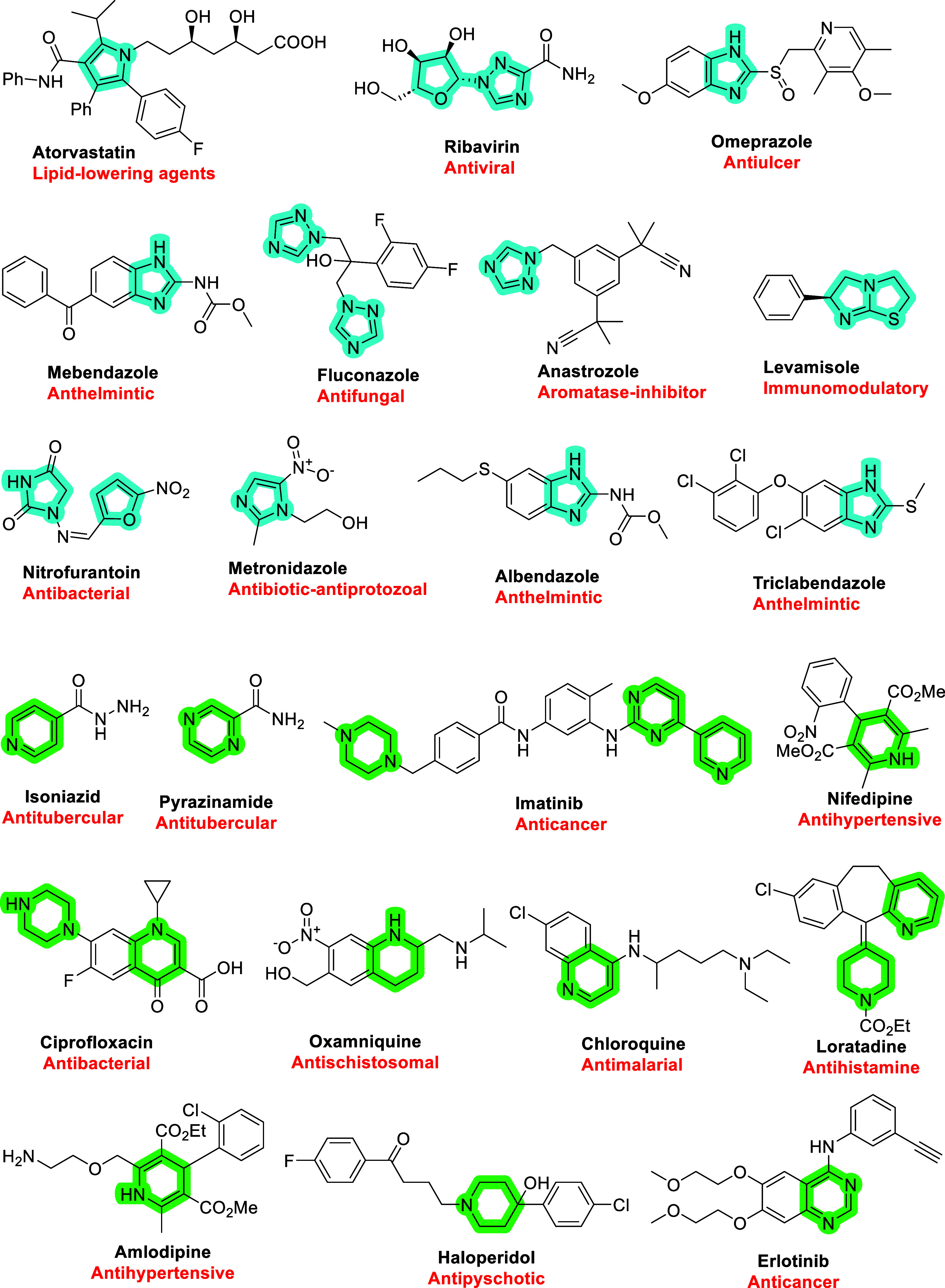
WHO essential medicines featuring five and six-membered *N*-heterocycles.

In this context, 1,3,4-oxadiazoles, a well-established class of five-membered *N*-heterocycles, have been extensively explored for their pharmacological potential. Their aromatic nature, combined with nitrogen and oxygen atoms, enhances metabolic stability, hydrogen bonding potential, and bioavailability. Recent studies report a wide range of pharmacological activities for these scaffolds, including anticancer,^1–3^ anti-inflammatory,^4,5^ antimicrobial,^6^ antiviral,^7^ and antioxidant^8^ effects. Their ability to act as versatile building blocks in the design of kinase inhibitors,^9,10^ antiangiogenic agents,^11^ immunomodulators, neuroprotective agents,^11^ and antidiabetic^12^ compounds further underscores their broad utility in drug discovery pipelines.

Similarly, pyridine-based six-membered *N*-heterocycles have played a pivotal role in the development of therapeutics across various disease areas, particularly in anticancer drug discovery. The presence of a nitrogen atom in the pyridine ring enhances interactions with biological targets through hydrogen bonding, π–π stacking, and metal coordination, which contribute to improved pharmacokinetic profiles and receptor binding affinity. Pyridine scaffolds are integral components of several FDA-approved anticancer agents, including regorafenib, sorafenib, nilotinib, and abemaciclib, underscoring their value in rational drug design. Incorporating pyridine into molecular frameworks often leads to enhanced bioavailability, metabolic stability, and target specificity, making it a favored structural motif in the design of small-molecule inhibitors. The continued exploration of pyridine-containing compounds has resulted in the development of numerous EGFR,^13^ VEGFR,^14^ and CDK^13^ pathway inhibitors, further affirming the importance of this scaffold in modern medicinal chemistry.^15–17^

Given the pharmacological significance of both five- and six-membered *N*-heterocycles, integrating them into a single molecular framework may lead to hybrid molecules with enhanced therapeutic properties. In this study, a new series of pyridine-based 1,3,4-oxadiazole derivatives was designed by integrating pyridine and 2,5-disubstituted 1,3,4-oxadiazoles using a thioether linker, known for enhancing lipophilicity and membrane permeability. This rational design aims to optimize drug-likeness and bioactivity while leveraging the complementary features of both heterocycles (Figure 2).

**Figure 2:**
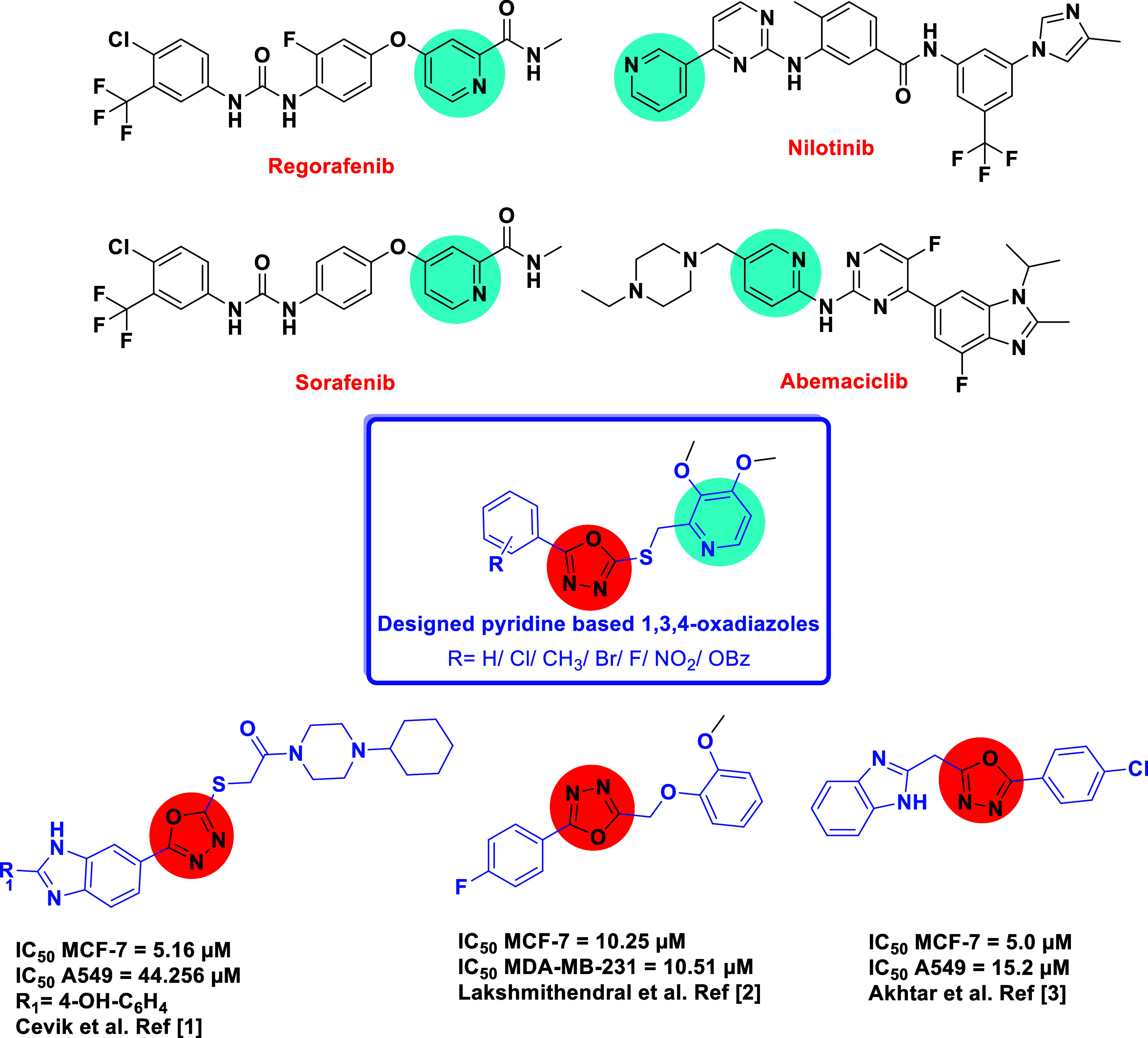
Design of pyridine-based 1,3,4-oxadiazole derivatives.

This work presents the synthesis, structural elucidation, computational analysis, and cytotoxic evaluation of pyridine-based 1,3,4-oxadiazole derivatives. FTIR, ^1^H NMR, ^13^C NMR and HRMS analyses were conducted for all synthesized compounds, ensuring comprehensive structural confirmation. Additionally, SCXRD and Hirshfeld surface analyses were performed on the meta-methyl-substituted derivative to gain deeper insights into its crystal structure and intermolecular interactions. MTT assays were performed on A549 lung cancer cells to assess their cytotoxic potential. Computational studies, including molecular docking, molecular dynamics simulations, and ADME analysis, offered insights into the binding properties and drug-likeness of the synthesized molecules.

## 2 Results and Discussion

### 2.1 Synthesis and Characterization

A new series of pyridine–oxadiazole compounds **5a–l** was synthesized through a four-step process. Initially, aromatic benzoic acids **1a–l** were esterified to form the corresponding esters **2a–l**. These aromatic esters were then treated with hydrazine hydrate, resulting in acid hydrazides **3a–l**. Cyclization of these acid hydrazides with carbon disulfide in an alkaline medium (potassium hydroxide) produced thio-1,3,4-oxadiazole derivatives **4a–l**, which served as key intermediates.^18^ Finally, these intermediates were condensed with 2-(chloromethyl)-3,4-dimethoxypyridine to yield the target pyridine–oxadiazole derivatives **5a–l** (Scheme 1). The products were purified by recrystallization, and their structures were confirmed using FTIR, ^1^H NMR, ^13^C NMR and HRMS.

**Scheme 1:**
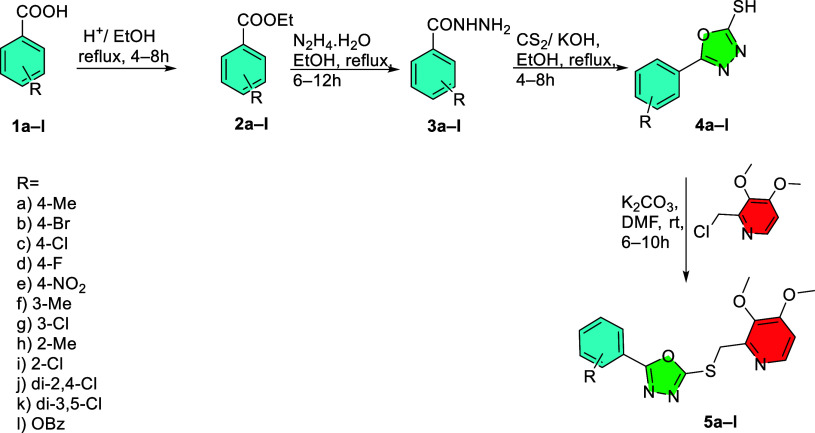
Synthesis of Pyridine-Based 1,3,4-Oxadiazole Derivatives **5a–l**

The FTIR analysis of these derivatives indicated the presence of the –C=N functional group, with absorption bands appearing within 1544–1617 cm^–1^. The ^1^H NMR spectra displayed aromatic proton signals in the range of 6.80–8.40 ppm, while the methoxy groups on the pyridine ring appeared as distinct singlets at 3.80–3.95 ppm. The CH_2_ group was observed as a singlet between 4.66 and 4.73 ppm, except in derivative **5e**, where the presence of an electron-withdrawing nitro group caused a downfield shift to 5.47 ppm. The benzyloxy-substituted derivative **5l** exhibited a singlet at 5.22 ppm for the OCH_2_ group. For para-, meta-, and ortho-substituted derivatives (**5a**, **5f**, and **5h**), the methyl protons resonated at 2.40, 2.41, and 2.60 ppm, respectively.

To further illustrate, the ^1^H NMR spectrum of derivative **5a** confirms the presence of pyridine, oxadiazole, and phenyl rings. In the aromatic region, four distinct doublets were observed. Two of these corresponded to the pyridine ring protons, with one appearing significantly deshielded at 8.15 ppm because of its proximity to the nitrogen atom, while the other resonated at 7.11 ppm. The remaining two doublets were attributed to the phenyl group attached to the oxadiazole core, with the aromatic proton closer to the oxadiazole ring appearing more deshielded (7.87 ppm) compared to the other (7.41 ppm). The methyl group attached to the phenyl ring appeared as a singlet at 2.40 ppm. Additionally, two methoxy groups were observed as singlets at 3.82 and 3.90 ppm. The methylene bridge connecting these heterocycles appeared as a singlet at 4.68 ppm.

In the ^13^C NMR spectra of derivatives **5a–l**, the methoxy carbons attached to the pyridine ring were observed as distinct singlets in the range of 54.75–64.05 ppm. The methylene carbon that links the oxadiazole and pyridine rings exhibited a singlet between 32.98 and 34.38 ppm, except for nitro derivative **5e**, where the carbon was deshielded to 56.50 ppm because of the electron-withdrawing effect of the nitro group. In the aromatic region, the carbons located between the oxygen and nitrogen atoms in the oxadiazole ring appeared highly deshielded, resonating in the range of 158.73–165.95 ppm. For the para-, meta- and ortho-substituted methyl derivatives (**5a**, **5f** and **5h**), the methyl carbons resonated at 21.61, 21.28, and 21.83 ppm, respectively. The benzyloxy derivative **5l** displayed a carbon peak for the OCH_2_ group at 70.01 ppm. In derivative **5d**, the fluorine atom at the para position caused neighboring carbon peaks to split into doublets due to fluorine–carbon coupling (*J* ^1^ = 252 Hz, *J* ^2^ = 22 Hz, *J* ^3^ = 9 Hz, *J* ^4^ = 3 Hz) (Figure 3). HRMS spectra further confirmed the formation of the desired compounds, with the observed molecular masses closely matching their calculated values.

**Figure 3:**
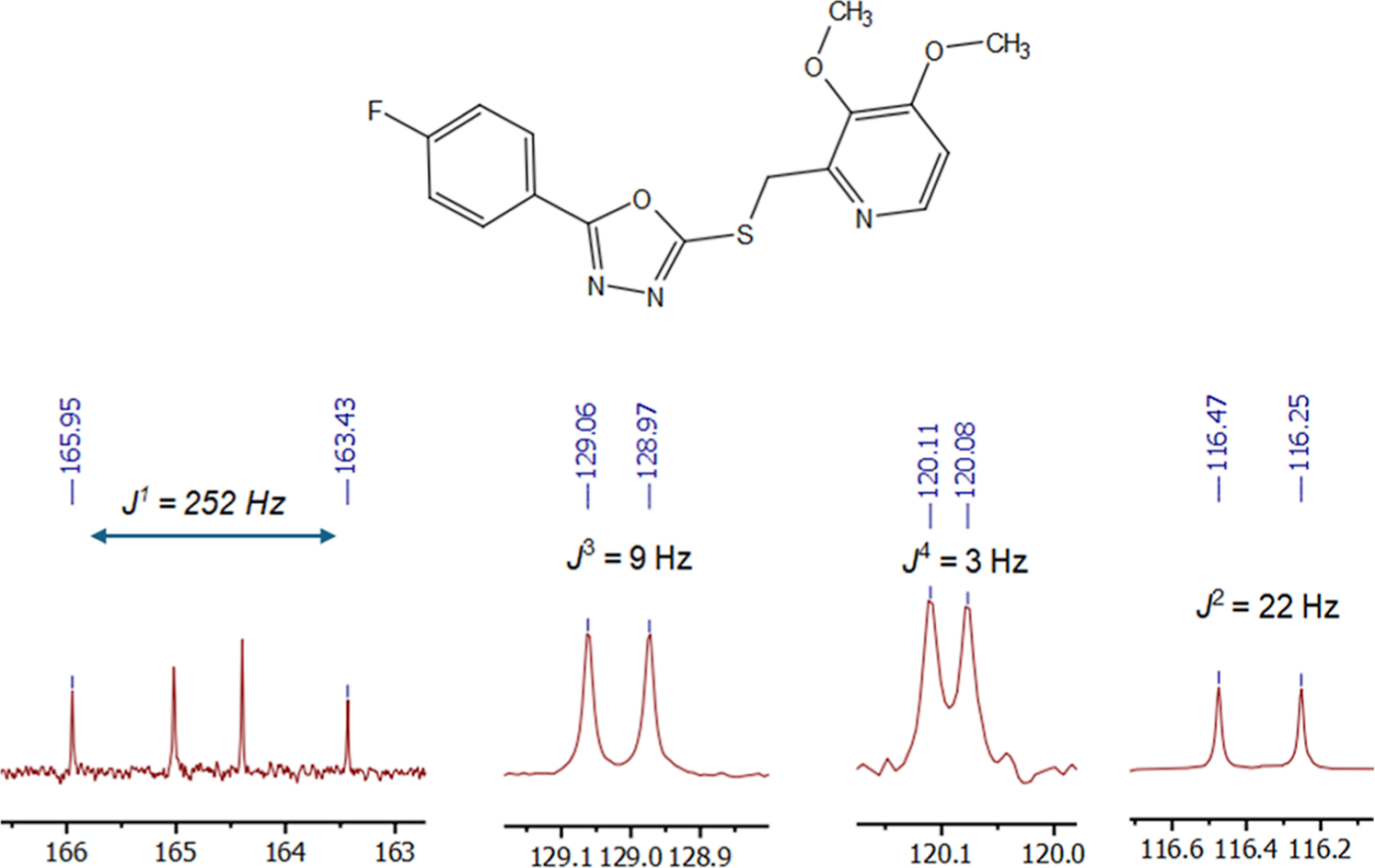
Carbon–Fluorine coupling observed in the ^13^C NMR (100 MHz, CDCl_3_) spectrum of compound **5d**, indicating characteristic *J*(C–F) splitting patterns.

### 2.2 Single Crystal-XRD

The crystallographic analysis of compound **5f** revealed that the molecule adopts a centrosymmetric arrangement in the orthorhombic crystal system with the space group *Pbca*, comprising eight molecules per unit cell (*Z* = 8). The unit cell parameters were established as *a* = 14.583(3) Å, *b* = 9.3773(19) Å, and *c* = 24.386(4) Å, with all angles (α = β = γ = 90°), confirming the orthorhombic geometry. The cell volume was calculated as 3334.8(11) Å^3^, and the crystal density was determined to be 1.368 g/cm^3^. The calculated value of *F*(000) was 1440 electrons. The Oak Ridge Thermal Ellipsoid Plot (ORTEP) at a 50% probability level is illustrated in Figure 4, providing detailed structural insights into the molecular arrangement. The structural refinement data and parameters are summarized in Table 1. The crystal structure has been deposited in the Cambridge Crystallographic Data Centre under deposition number 2406362 and is accessible at http://www.ccdc.cam.ac.uk.

**Figure 4:**
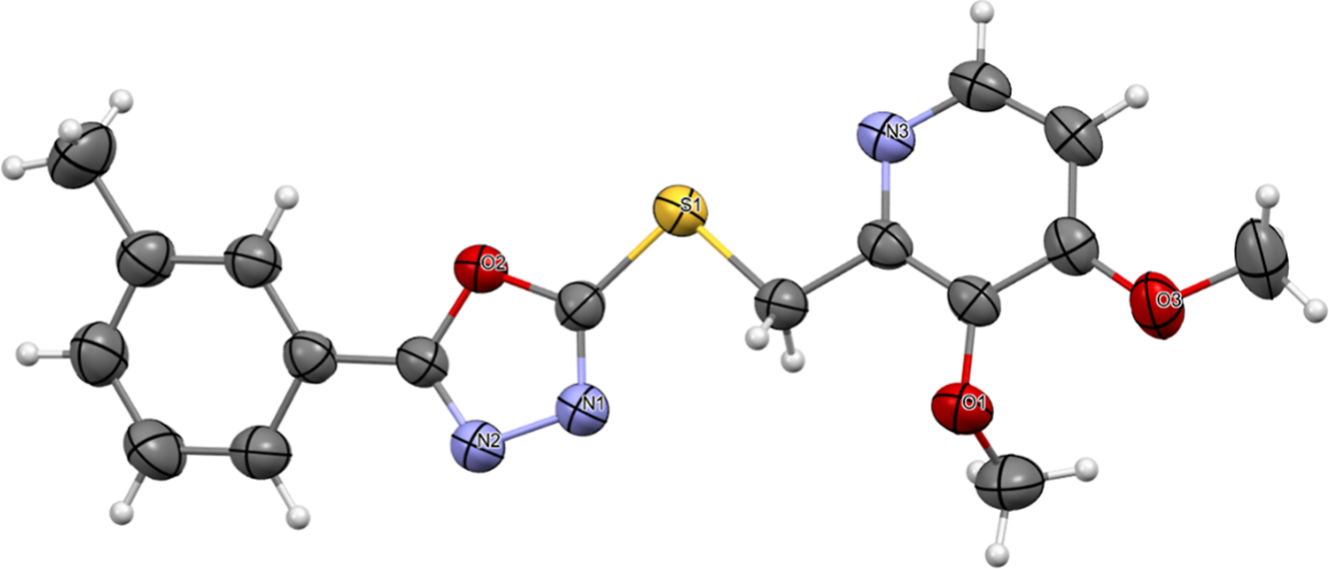
ORTEP representation of derivative **5f** with 50% probability ellipsoids.

**Table 1:** X-ray Crystallographic Data and Refinement Factors of Derivative **5f**

crystallographic parameters	value	
chemical formula	C_17_H_17_N_3_O_3_S	
formula weight	343.39 g/mol	
temperature	300(2) K	
crystal size	0.152 × 0.165 × 0.189 mm	
crystal habit	colorless crystal	
crystal system	orthorhombic	
space group	*Pbca*	
unit cell dimensions	*a* = 14.583(3) Å	α = 90°
	*b* = 9.3773(19) Å	β = 90°
	*c* = 24.386(4) Å	γ = 90°
volume	3334.8(11) Å^3^	
*Z*	8	
absorption coefficient	0.215 mm^–1^	
density (calculated)	1.368 g/cm^3^	
*F*(000)	1440	

### 2.3 Hirshfeld Analysis

Hirshfeld surface analysis is a powerful computational technique used to visualize and quantify intermolecular interactions in crystal structures. This method provides a detailed understanding of molecular packing and noncovalent interactions, which play crucial roles in determining the stability and properties of crystalline materials. The analysis is based on the partitioning of the total electron density in a crystal, allowing for the identification of close intermolecular contacts and interaction patterns.

In this study, Hirshfeld surface analysis of the meta-methyl-substituted pyridine-based 1,3,4-oxadiazole derivative **5f** was performed using CrystalExplorer 17. The *d*_norm_-mapped surfaces highlighted significant intermolecular interactions, with red regions indicating close contacts, whereas blue and white regions represented longer contacts or nonsignificant interactions (Figure 5).^19^ Additional mappings of *d*_i_, *d*_e_, and curvedness further characterized the surface properties, confirming the presence of strong intermolecular forces along different crystallographic axes.

**Figure 5:**
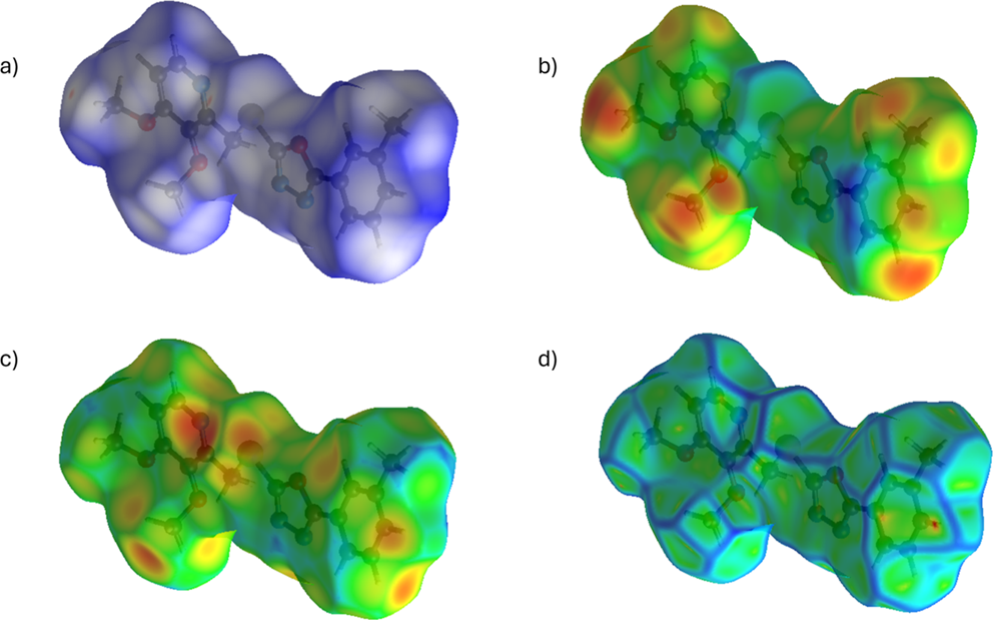
Hirshfeld surface analysis of the molecule **5f**, illustrating various surface properties. (a) *d*_norm_ Mapped in the range −0.1021 to 1.2464 au, highlighting significant intermolecular interactions; red regions indicate close contacts, while blue and white regions represent longer or insignificant interactions. (b) *d*_i_ mapped in the range 1.0369–2.5831 au, representing internal atomic distances from the surface. (c) *d*_e_ mapped in the range 1.0371–2.5413 au, indicating external atomic distances from the surface. (d) Curvedness mapped in the range −4.00 to 4.00 au, revealing surface shape variations and molecular interaction patterns.

The quantitative description of interatomic contacts can be obtained from the two-dimensional (2D) fingerprint plots. Figure 6 shows the reciprocal contacts observed in the supramolecular assembly. Hydrogen bonding played a major role in crystal packing, with N···H (14.8%) and O···H (11.0%) interactions contributing to molecular stabilization. H···H interactions (45.0%) dominated the surface, while C···H (10.4%) and C···C (4.9%) interactions suggested π–π stacking and van der Waals forces. S···H interactions (6.9%) indicated a minor role of sulfur in intermolecular contacts. Other interactions, such as O–C (3%), N–C (2.4%), S–C (1.1%), N–N (0.3%), and S–N (0.3%), also played minor roles in the supramolecular assembly.

**Figure 6:**
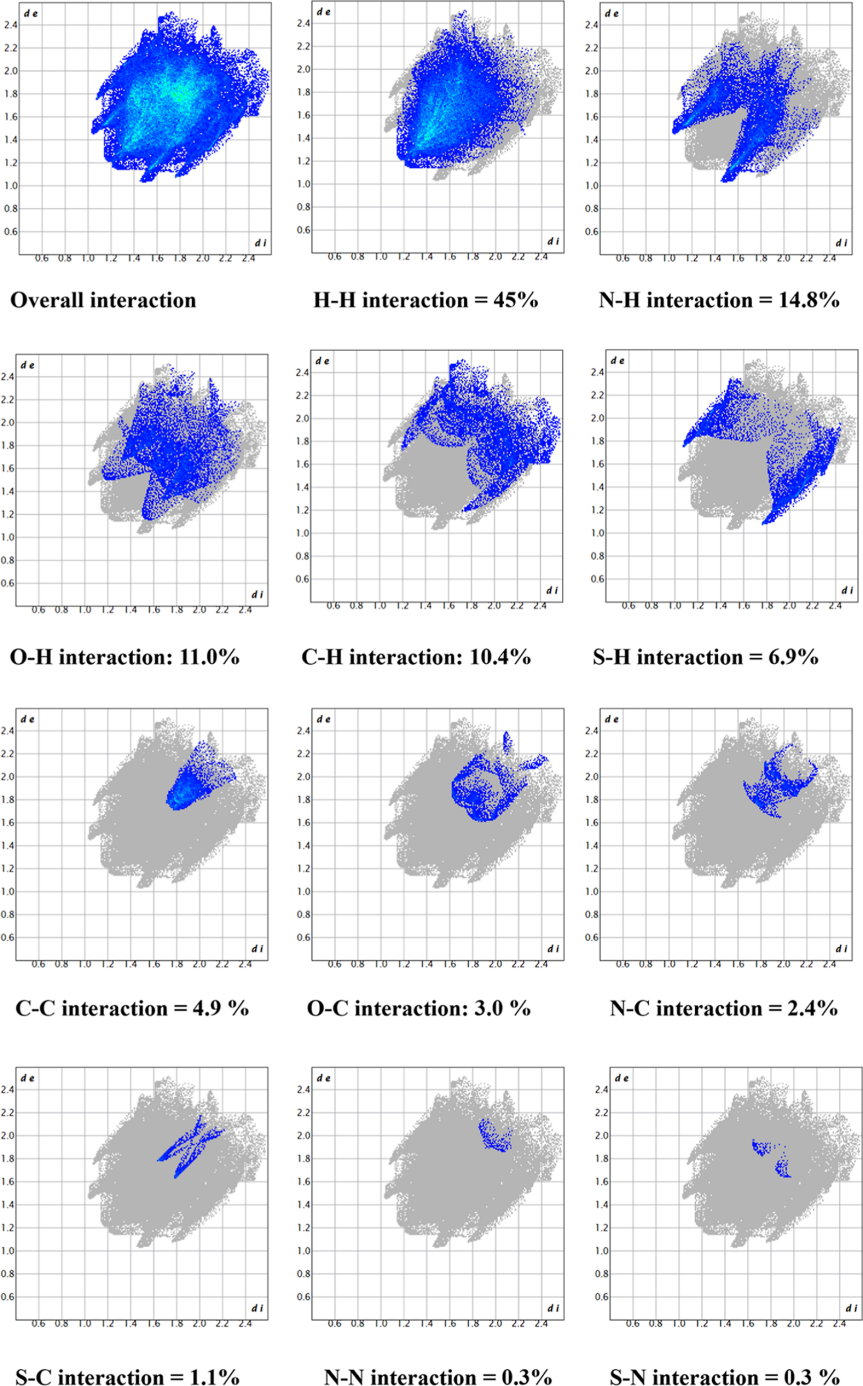
2D fingerprint plots illustrating the contributions of various intermolecular interactions to the crystal packing of **5f**.

Electrostatic potential calculations at the B3LYP/6-31G­(d,p) level revealed a charge distribution range of −0.080 to 0.0632 au, identifying donor and acceptor groups. The red and blue regions on the electrostatic potential surface, shown in Figure 7, represent the H-bond donor and acceptor groups, respectively.

**Figure 7:**
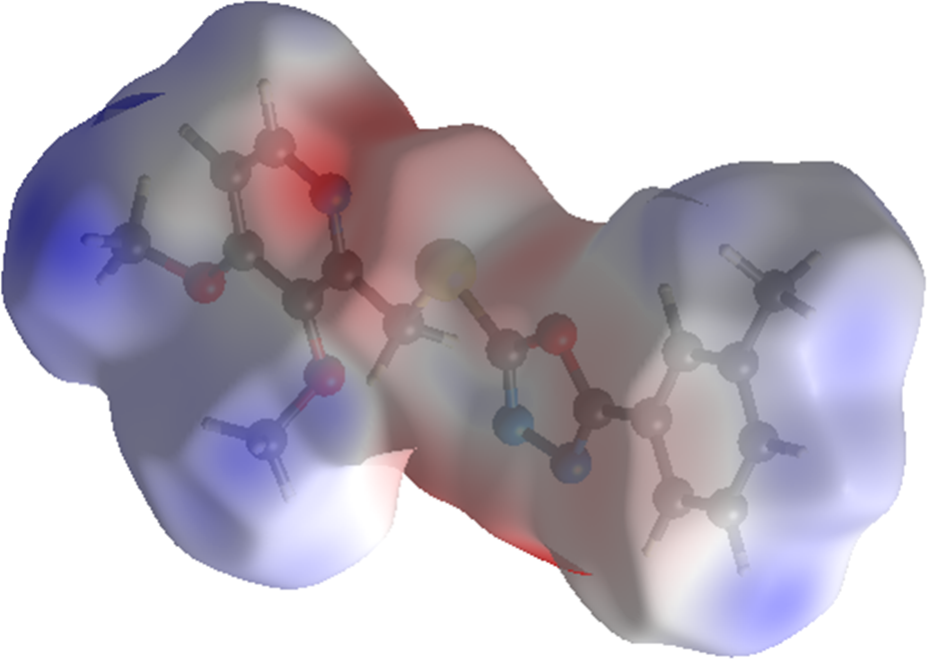
Electrostatic potential map illustrating charge distribution in the crystal structure of **5f**.

Void analysis revealed a calculated void volume of 354.45 Å^3^ and a void percentage of 10.63%, suggesting efficient molecular packing with no large cavities in the crystal structure. The void surface area was 1264.39 Å^2^, with globularity (0.192) and asphericity (0.248) values indicating an anisotropic molecular arrangement (Figure S57). Energy framework calculations provided a visualization of the stabilizing interactions, highlighting the dominant role of dispersion forces in crystal lattice stability (Figure S59). The total energy framework, which was mapped along different crystallographic axes, underscored the importance of weak intermolecular forces in maintaining the structural integrity of **5f**. The molecular packing was further analyzed using interaction energy calculations with TONTO (built-in in CrystalExplorer), which considers a molecular cluster within a 3.8 Å radius. The total energy was computed as the sum of the Coulomb electrostatic, dispersion, polarization, and repulsion energies (Table S11). The Coulomb electrostatic energy was highest for the molecular pair with a centroid distance of 10.11 Å, whereas the dispersion energy was greatest for the pair with a centroid distance of 4.9 Å. Consequently, the total energy also reached its maximum magnitude for the molecular pair at 4.9 Å. The energy framework analysis of Coulombic, dispersion, and total energies is shown in Figure 8. The cylinders in the figures join the molecular centers, and their width is proportional to the strength of the corresponding energies. As mentioned above, the dispersion energy plays a critical role in the stabilization of the supramolecular structure.

**Figure 8:**
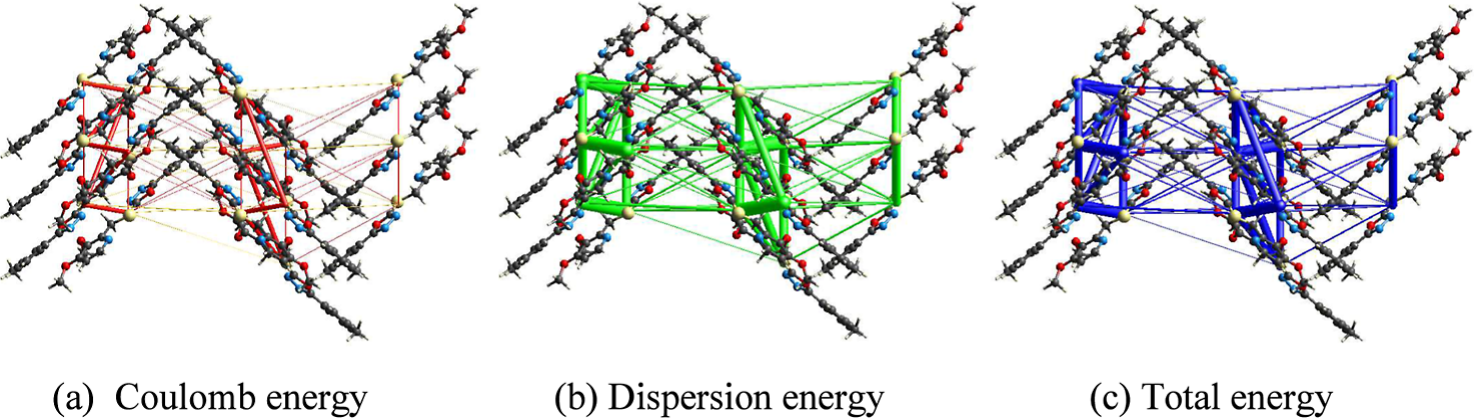
Energy framework for Coulomb electrostatic, dispersion and total energies in molecule **5f**.

### 2.4 Cytotoxicity Evaluation

The MTT assay was conducted on A549 cells after 72 h of incubation to assess the cytotoxic potential of the synthesized pyridine-based 1,3,4-oxadiazole derivatives. Among the tested compounds, derivative **5k** exhibited the highest cytotoxicity (POI = 59.57 ± 15.01%) at 10 μM, closely approaching the activity of the reference drug 5-fluorouracil (5-Fu, POI = 62.65 ± 10.87%) (Figure 9, Table 2). The enhanced activity of **5k** can be attributed to the presence of a 3,5-dichlorophenyl moiety, where symmetrical meta-disubstitution with electron-withdrawing chlorine atoms increases lipophilicity and redistributes electron density across the aromatic ring, facilitating favorable interactions at the binding site.

**Figure 9:**
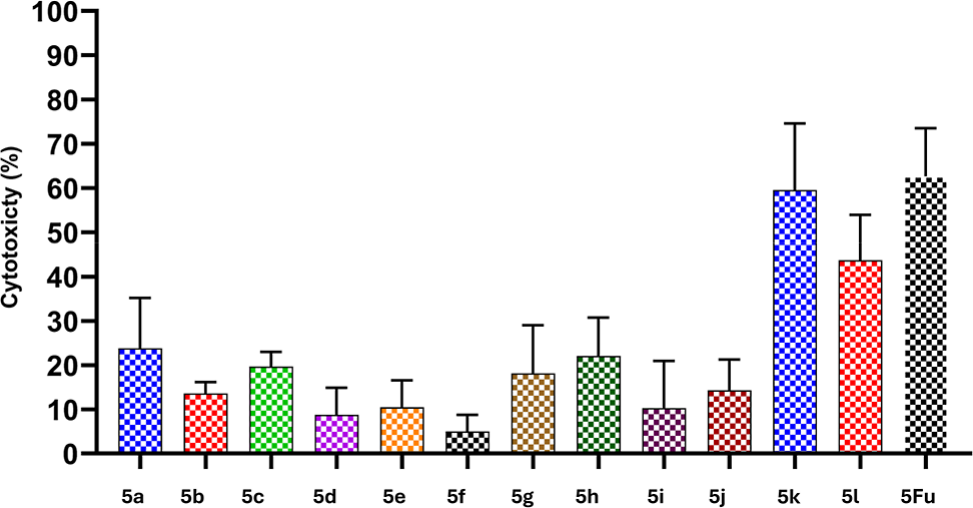
Graphical representation of the percentage of cytotoxicity (±S.D.) of pyridine-oxadiazole hybrids at 10 μM.

**Table 2:** Percentage of Inhibition (POI ± S.D.) of Pyridine-Oxadiazole Hybrids at 10 μM

compounds	POI ± SD
**5a**	23.79 ± 11.43
**5b**	13.61 ± 2.575
**5c**	19.7 ± 3.319
**5d**	8.745 ± 6.111
**5e**	10.49 ± 6.059
**5f**	4.964 ± 3.775
**5g**	18.16 ± 10.87
**5h**	22.09 ± 8.654
**5i**	10.29 ± 10.68
**5j**	14.3 ± 6.984
**5k**	59.57 ± 15.01
**5L**	43.72 ± 10.22
**5Fu**	62.65 ± 10.87

The benzyloxy-substituted derivative **5l** also demonstrated appreciable cytotoxicity (43.72 ± 10.22%), ranking second among the series. The benzyloxy group enhances membrane permeability and support hydrophobic or π–π stacking interactions with the target. In contrast, monosubstituted derivatives such as **5f** (3-CH_3_) and **5g** (3-Cl) showed lower activity indicating that a single meta substituent does not contribute significantly to bioactivity. Similarly, para-substituted analogs like **5a** (4-CH_3_) and **5c** (4-Cl) exhibited moderate activity, likely due to electronic influences. Derivatives bearing bulky or strongly electron-withdrawing para-substituents, such as **5b** (4-Br), **5d** (4-F), and **5e** (4-NO_2_), displayed poor cytotoxicity, possibly due to steric hindrance or unfavorable desolvation energies that limit target engagement and membrane permeability. Likewise, ortho-substituted analogs including **5h** (2-CH_3_) and **5i** (2-Cl) showed reduced activity suggesting that steric clashes at the ortho position hinder optimal molecular orientation for binding.

To further quantify cytotoxic potency, the IC_50_ values of **5k** and 5-Fu were determined to be 6.99 ± 3.15 μM and 6.41 ± 3.55 μM, respectively (Figure 10), confirming the promising anticancer potential of **5k**. Future work will aim to evaluate its cytotoxicity in normal human lung fibroblast cells (e.g., MRC-5) to assess selectivity and determine its therapeutic index.

**Figure 10:**
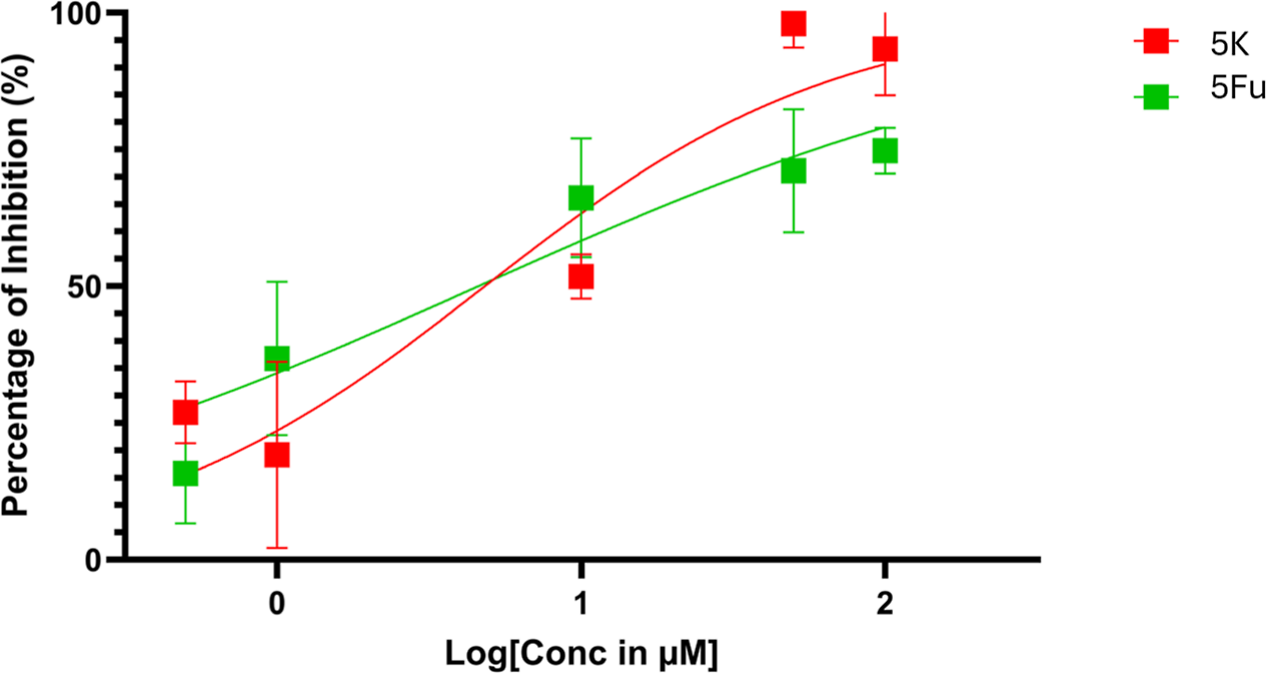
IC_50_ plots of the pyridine-based 1,3,4-oxadiazole derivative **5k** and 5-fluorouracil (5-Fu).

### 2.5 ADME Analysis

ADME analysis offers critical insights into the pharmacokinetic properties of the ligands. Derivatives **5a–j** adhered to Lipinski’s rule of five (ROF) without any violations, whereas **5l** exhibited a single violation, which falls within the permissible range. Most of the synthesized oxadiazole derivatives exhibited high Caco-2 permeability (QPPCaco) scores, suggesting strong intestinal absorption, whereas the nitro-substituted derivative **5e** demonstrated the lowest permeability. Additionally, in silico ADME predictions revealed good human oral absorption (HOA) and adequate aqueous solubility (QPlogS). Other parameters, such as polar surface area (PSA), binding potential to human serum albumin (QPlogKhsa), and compliance with the rule of three (ROT), also remained within acceptable limits (Table 3).^20,21^

**Table 3:** QikProp ADME Values of the Synthesized Pyridine-Based 1,3,4-Oxadiazole Derivatives^a^

derivative	MolW	HD	HA	QP log Po/w	ROF	ROT
**5a**	343.400	0	5	4.019	0	0
**5b**	408.269	0	5	4.278	0	0
**5c**	363.818	0	5	4.198	0	0
**5d**	347.363	0	5	4.050	0	0
**5e**	374.370	0	6	2.941	0	0
**5f**	343.400	0	5	4.068	0	0
**5g**	363.818	0	5	4.054	0	0
**5h**	343.400	0	5	3.967	0	0
**5i**	363.818	0	5	4.135	0	0
**5j**	398.263	0	5	4.641	0	1
**5k**	398.263	0	5	4.704	0	1
**5l**	435.497	0	5.75	5.094	1	1
**RV**	130–725	0–6	2.0–20.0	–2–6.5	Max 4	Max 3

title	QPPCaco	QPlogKhsa	QPlogS	HOA	PSA
**5a**	2128.582	0.273	–5.208	3	64.906
**5b**	2137.330	0.252	–5.492	3	64.878
**5c**	2140.046	0.226	–5.367	3	64.879
**5d**	3728.939	0.112	–4.778	3	60.530
**5e**	253.859	0.001	–4.645	3	109.935
**5f**	2535.817	0.266	–5.208	3	62.755
**5g**	1883.852	0.198	–5.006	3	62.976
**5h**	2387.138	0.234	–4.808	3	60.558
**5i**	2938.016	0.179	–4.989	3	60.960
**5j**	2944.624	0.307	–5.755	3	60.868
**5k**	2122.394	0.355	–6.135	3	64.946
**5l**	2135.654	0.509	–5.201	3	65.087
**RV**	<25 poor	–1.5–1.5	–6.5–0.5	1-low	max 200
	>500 great			2-med	
				3-high	

a MolW—molecular weight, HD—hydrogen bond donor, HA—hydrogen bond acceptor, QPlogPo/w—predicted octanol–water partition coefficient, RV—recommended values.

### 2.6 Docking Studies

Molecular docking is a cornerstone technique in medicinal chemistry, widely employed to predict ligand–protein interactions and provide crucial insights for drug development. In this study, the binding potential of the synthesized hybrid molecules with cyclin-dependent kinase 2 (CDK2) was evaluated. CDKs are intracellular serine/threonine kinases that regulate the cell cycle and transcription. Their dysregulation is frequently observed in various cancers, underscoring their importance as therapeutic targets in oncology.^22–24^ The crystal structure of CDK2 (PDB ID: 2XMY; resolution: 1.90 Å) was utilized for docking analysis.^25–34^ To validate the docking protocol, the minimized cocrystal structure was compared with the docked pose, yielding a root-mean-square deviation (RMSD) of 1.033 Å. This value, well within the acceptable threshold of 2 Å, confirmed the accuracy and reliability of the docking methodology.^35,36^

Figure S60 illustrates the 2D docking poses of the pyridine-based oxadiazole derivatives within the CDK2 binding site. Derivative **5a** exhibited a hydrogen bond with Leu83, whereas **5b** formed a hydrogen bond with Lys89 and a halogen bond with Asp145. Derivative **5c** showed a water-mediated hydrogen bond with Asp86 and a halogen bond with Leu83. Both **5f** and **5g** displayed π–π interactions with Phe80. Additionally, **5i** formed a hydrogen bond with Asp86, whereas **5j** exhibited a water-mediated hydrogen bond with Asp86 and a halogen bond with Leu83. Derivative **5k** demonstrated a hydrogen bond with Lys89 and water-mediated polar interactions (H-bond and halogen bond) with Asp145. Lastly, **5l** showed both a water-mediated hydrogen bond and a direct hydrogen bond with Asp86 (Table S12).

### 2.7 Molecular Dynamics

Derivative **5k**, a 3,5-dichloro-substituted pyridine-based 1,3,4-oxadiazole, was selected for molecular dynamics simulation based on its highest cytotoxic activity. The RMSD of the resulting protein–ligand complex (2XMY-5k) stabilized after initial fluctuations and remained between 2.4 Å and 2.8 Å (Figure 11). This range falls within the acceptable threshold for small proteins, indicating that the complex maintained structural stability throughout the 100 ns simulation. After initial stabilization, both the protein and ligand maintained a steady RMSD pattern, indicating that the ligand remained stably bound within the protein binding site throughout the simulation.

**Figure 11:**
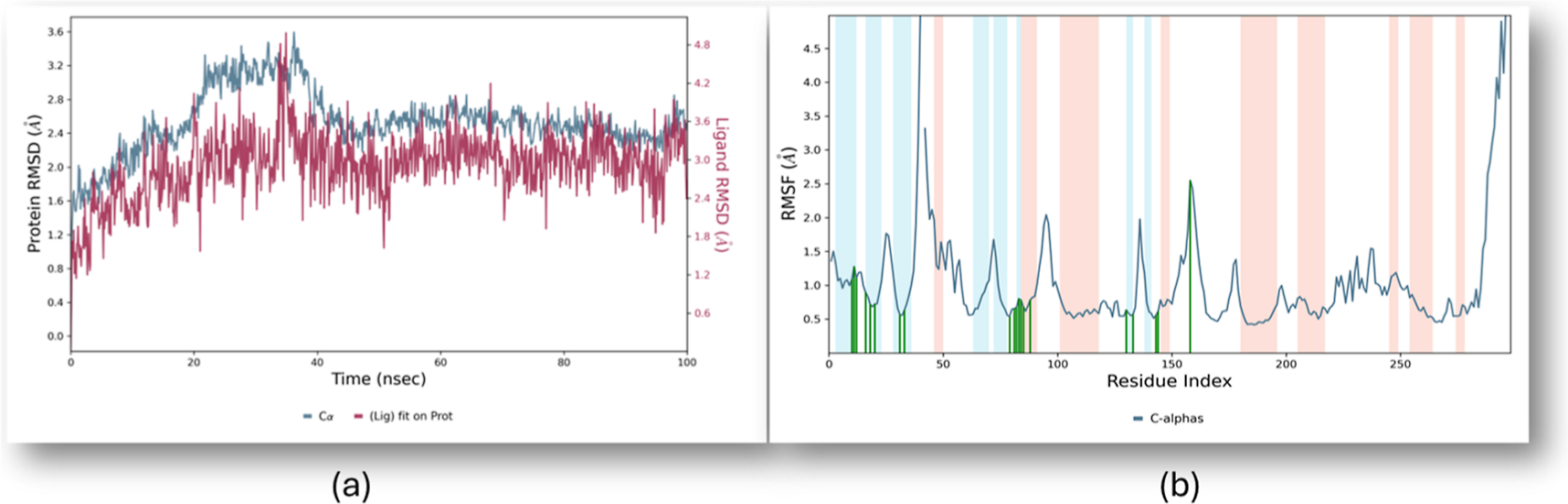
(a) RMSD plot depicting protein and ligand dynamics. Protein RMSD is shown in green (left *y*-axis), and ligand RMSD in maroon (right *y*-axis), plotted against simulation time (*x*-axis). (b) RMSF visualization of the protein–ligand complex. Secondary structure elements are color-coded: α-helices in orange, β-strands in sky blue, and loop regions in white. Green vertical lines indicate residues that interact with ligand **5k** (3,5-dichloro-substituted pyridine-based 1,3,4-oxadiazole derivative) during the simulation.

The RMSF analysis provided valuable insights into the flexibility of specific regions within the protein chain. Residues within the α-helical and beta-strand regions exhibited low fluctuations (RMSF < 2.0 Å), signifying stable secondary structures. In contrast, loop regions displayed greater flexibility, with fluctuations observed around residue indices 40–50 and >260, where RMSF values exceeded 3.0 Å. Key ligand-binding residues are marked by green vertical bars on the RMSF plot, emphasizing stable interactions primarily within the α-helical and beta-strand regions (Figure 11).

Protein–ligand contact analysis identified critical interactions contributing to the stability of the complex. A hydrogen bond with Leu83 was present for 83% of the simulation time, whereas two water-mediated hydrogen bonds with His84 and Asp86 were observed for 25% and 36% of the simulation time, respectively (Figure 12). The primary residues contributing to total interactions included Ile10 (59%), Ala31 (62%), Leu83 (98%), Asp86 (48%), and Leu134 (64%) (Figure 13).

**Figure 12:**
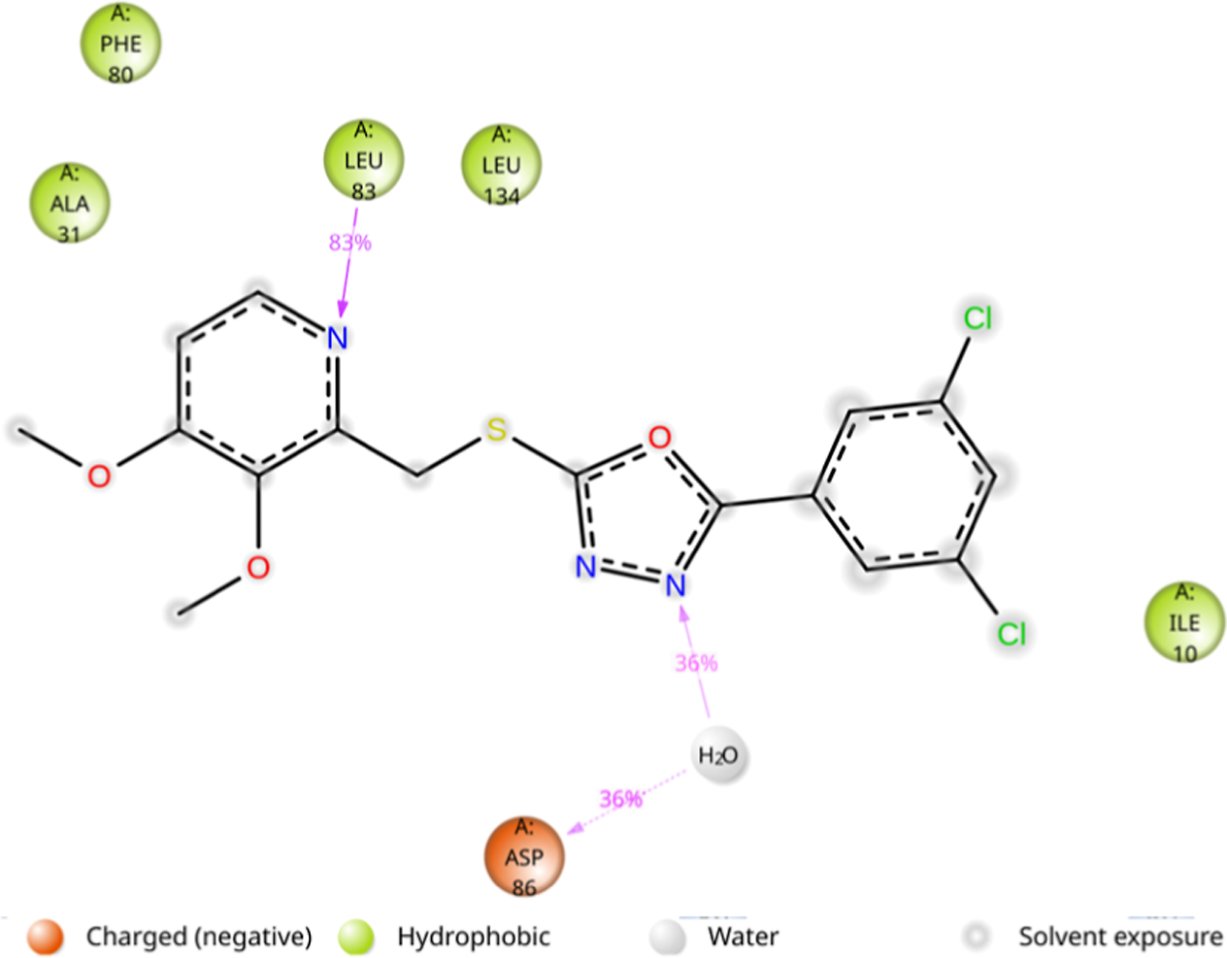
2D illustration of ligand–protein binding contacts between **5k** and the 2XMY protein during the MD simulation.

**Figure 13:**
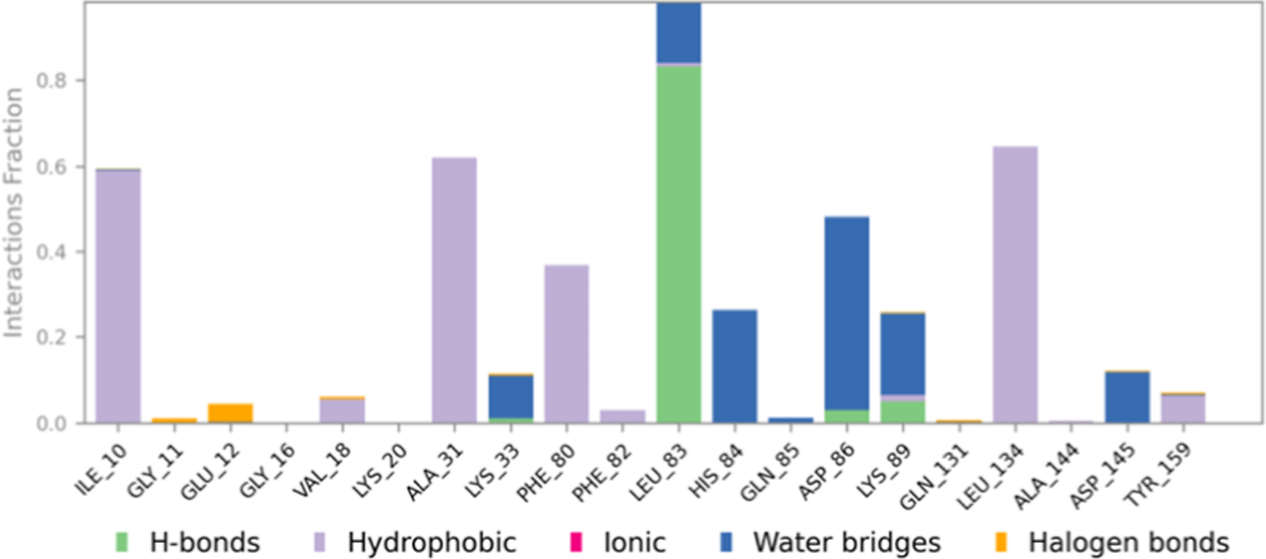
Histogram of protein–ligand contacts observed during the 100 ns MD simulation of the 2XMY–5k complex.

To further evaluate the structural compactness and solvent exposure of the 2XMY–5k complex, additional MD parameters including radius of gyration (*R*_g_), solvent-accessible surface area (SASA), polar surface area (PSA), and molecular surface area (MolSA) were analyzed specifically for compound **5k**. The Rg profile remained stable throughout the 100 ns simulation, fluctuating between 4.0 and 5.0 Å, indicating that the ligand maintained a compact conformation within the binding pocket (Figure S61). SASA values ranged from 50 to 200 Å^2^, with no significant deviations over time, suggesting consistent solvent exposure and the absence of major conformational changes (Figure S61). PSA values remained within 72–96 Å^2^, while MolSA values fluctuated between 300 and 330 Å^2^, both exhibiting stable trends that reflect consistent polar and molecular surface characteristics throughout the simulation. These findings, supported by RMSD and RMSF analyses, highlight the strong and stable affinity between derivative **5k** and the 2XMY protein, reinforcing the favorable binding behavior and overall structural stability of the complex.

## 3 Conclusion

In this study, a series of 12 novel pyridine–1,3,4-oxadiazole hybrids was successfully synthesized and comprehensively characterized using spectroscopic and crystallographic techniques. Single-crystal XRD and Hirshfeld surface analysis of the meta-methyl derivative provided detailed insights into molecular conformation and intermolecular interactions. Among the synthesized compounds, **5k**, bearing a 3,5-dichloro-substituted phenyl ring, exhibited the highest cytotoxicity against A549 lung cancer cells, with an IC_50_ value comparable to 5-fluorouracil. SAR analysis indicated that meta-substitution plays a key role in enhancing activity, while bulky or strongly electron-withdrawing substituents attenuate cytotoxic effects.

In silico studies further substantiated these findings: compound **5k** displayed favorable ADME properties, strong binding affinity to CDK2 in docking studies, and stable interaction profiles in 100 ns molecular dynamics simulations. Collectively, these results support the potential of pyridine–oxadiazole hybrids, particularly **5k**, as promising candidates for the development of anticancer therapeutics. Future work will focus on evaluating their selectivity, in vivo efficacy, and mechanistic pathways to further advance these scaffolds toward clinical relevance.

## 4 Materials and Methods

### 4.1 Chemistry

All reagents and solvents were procured from commercial sources and utilized without further purification. Fourier-transform infrared (FTIR) spectra of the pyrazoline derivatives were recorded using a Shimadzu FTIR spectrophotometer. Nuclear magnetic resonance (NMR) spectra, including ^1^H NMR (400 MHz) and ^13^C NMR (100 MHz), were acquired on a Bruker AM 400 MHz NMR spectrometer with tetramethylsilane (TMS) as an internal reference. High-resolution mass spectrometry (HRMS) data were obtained using a time-of-flight (TOF) electrospray ionization (ESI) technique. The progress and completion of the reaction were tracked by TLC on silica gel plates. Spots were visualized under ultraviolet light (365 nm). The melting points of the final products were determined using the open capillary method.

#### 4.1.1 Methods for the Synthesis of 2-(((3,4-Dimethoxypyridin-2-yl)­methyl)­thio)-5-substituted-phenyl-1,3,4-oxadiazoles (**5a–l**)

2-(Chloromethyl)-3,4-dimethoxypyridine (0.5 mmol) and thio-oxadiazoles (0.5 mmol) were stirred in DMF with K_2_CO_3_ at room temperature. The reaction progress was monitored using TLC (ethyl acetate/toluene, 1:3). After completion, cold water was added to the reaction mass, and the resulting solid was filtered. The residue was recrystallized from CH_3_OH to obtain the desired compounds (**5a–l**).

#### 4.1.2 Spectral Data

##### 4.1.2.1 2-(((3,4-Dimethoxypyridin-2-yl)­methyl)­thio)-5-(*p*-tolyl)-1,3,4-oxadiazole (**5a**)

Off white solid, mp 171–173 °C, yield = 90%, FTIR (cm^–1^): 3031 (C–H stretching), 1615 and 1585 (C=N stretching), 1501 (C=C stretching), (C–O–C stretching), 1060 (C–S stretching). ^1^H NMR (400 MHz, DMSO-*d*_6_): δ 8.15 (d, *J* = 5.5 Hz, 1H), 7.87 (d, *J* = 7.9 Hz, 2H), 7.41 (d, *J* = 7.9 Hz, 2H), 7.11 (d, *J* = 5.5 Hz, 1H), 4.68 (s, 2H), 3.90 (s, 3H), 3.82 (s, 3H), 2.40 (s, 3H) ppm. ^13^C NMR (100 MHz, DMSO-*d*_6_): δ 165.79, 163.72, 158.62, 148.82, 146.02, 143.32, 142.68, 130.47, 126.84, 120.81, 109.01, 61.02, 56.53, 34.37, 21.61 ppm. HRMS (ESI-TOF) calcd for C_17_H_17_N_3_O_3_S: [M + H]^+^, 344.1068; found, 344.0628.

##### 4.1.2.2 2-(4-Bromophenyl)-5-(((3,4-dimethoxypyridin-2-yl)­methyl)­thio)-1,3,4-oxadiazole (**5b**)

Off white solid, mp 170–172 °C, yield = 92%, FTIR (cm^–1^): 3050 (C–H stretching), 1604 and 1582 (C=N stretching), 1484 (C=C stretching), 1179 (C–O–C stretching), 1060 (C–S stretching), 718 (C–Br stretching). ^1^H NMR (400 MHz, DMSO-*d*_6_): δ 8.16 (d, *J* = 5.5 Hz, 1H), 7.92 (d, *J* = 8.5 Hz, 2H), 7.82 (d, *J* = 8.3 Hz, 2H), 7.11 (d, *J* = 5.5 Hz, 1H), 4.70 (s, 2H), 3.90 (s, 3H), 3.82 (s, 3H) ppm. ^13^C NMR (100 MHz, DMSO-*d*_6_): δ 165.05, 164.45, 158.62, 148.71, 146.02, 143.32, 133.02, 128.80, 126.10, 122.77, 109.04, 61.04, 56.54, 34.38 ppm. HRMS (ESI-TOF) calcd for C_16_H_14_BrN_3_O_3_S: [M + H]^+^, 408.0017; found, 408.0015.

##### 4.1.2.3 2-(4-Chlorophenyl)-5-(((3,4-dimethoxypyridin-2-yl)­methyl)­thio)-1,3,4-oxadiazole (**5c**)

White solid, mp 153–155 °C, yield = 88%, FTIR (cm^–1^): 3003 (C–H stretching), 1607 and 1582 (C=N stretching), 1487 (C=C stretching), 1291 (C–O–C stretching), 1062 (C–S stretching), 829 (C–Cl stretching). ^1^H NMR (400 MHz, CDCl_3_): δ 8.20 (d, *J* = 5.5 Hz, 1H), 7.96 (d, *J* = 8.3 Hz, 2H), 7.48 (d, *J* = 8.3 Hz, 2H), 6.82 (d, *J* = 5.5 Hz, 1H), 4.73 (s, 2H), 3.93 (s, 3H), 3.93 (s, 3H) ppm. ^13^C NMR (100 MHz, CDCl_3_): δ 163.98, 163.62, 157.57, 147.60, 144.70, 142.72, 136.80, 128.38, 126.96, 121.17, 106.55, 60.09, 54.76, 32.98 ppm. HRMS (ESI-TOF) calcd for C_16_H_14_ClN_3_O_3_S: [M + H]^+^, 364.0523; found, 364.0526.

##### 4.1.2.4 2-(((3,4-Dimethoxypyridin-2-yl)­methyl)­thio)-5-(4-fluorophenyl)-1,3,4-oxadiazole (**5d**)

White solid, mp 134–136 °C, yield = 94%, FTIR (cm^–1^): 3065 (C–H stretching), 1607 and 1588 (C=N stretching), 1465 (C=C stretching), 1300 (C–F stretching), 1286 (C–O–C stretching), 1066 (C–S stretching). ^1^H NMR (400 MHz, CDCl_3_): δ 8.20 (d, *J* = 5.5 Hz, 1H), 8.03 (dd, *J* = 8.5, 5.1 Hz, 2H), 7.19 (t, *J* = 8.5 Hz, 2H), 6.82 (d, *J* = 5.6 Hz, 1H), 4.73 (s, 2H), 3.94 (s, 3H), 3.94 (s, 3H) ppm. ^13^C NMR (100 MHz, CDCl_3_): δ 165.94 (d, *J* = 252 Hz), 165.01, 164.39, 158.60, 148.69, 145.73, 143.75, 129.06 (d, *J* = 9 Hz), 120.10 (d, *J* = 3 Hz), 116.47 (d, *J* = 22 Hz), 107.57, 61.12, 55.78, 34.02 ppm. HRMS (ESI-TOF) calcd for C_16_H_14_FN_3_O_3_S: [M + H]^+^, 348.0818; found, 348.0816.

##### 4.1.2.5 2-(((3,4-Dimethoxypyridin-2-yl)­methyl)­thio)-5-(4-nitrophenyl)-1,3,4-oxadiazole (**5e**)

White solid, mp 96–98 °C, yield = 85%, FTIR (cm^–1^): 3045 (C–H stretching), 1719 and 1592 (C=N stretching), 1524 (NO_2_ asymmetric stretching), 1494 (C=C stretching), 1349 (NO_2_ symmetric stretching) 1282 (C–O–C stretching), 1066 (C–S stretching). ^1^H NMR (400 MHz, DMSO-*d*_6_): δ 8.39–8.32 (m, 2H), 8.24–8.17 (m, 3H), 7.16 (d, *J* = 5.5 Hz, 1H), 5.47 (s, 2H), 3.91 (s, 3H), 3.81 (s, 3H) ppm. ^13^C NMR (100 MHz, DMSO-*d*_6_): δ 164.48, 158.73, 150.77, 148.28, 146.26, 143.99, 135.42, 131.22, 124.45, 109.35, 64.04, 61.17, 56.50 ppm. HRMS (ESI-TOF) calcd for C_16_H_14_N_4_O_5_S: [M + H]^+^, 375.0763; found, 375.0769.

##### 4.1.2.6 2-(((3,4-Dimethoxypyridin-2-yl)­methyl)­thio)-5-(*m*-tolyl)-1,3,4-oxadiazole (**5f**)

White solid, mp 108–110 °C, yield = 90%, FTIR (cm^–1^): 3088 (C–H stretching), 1585 and 1558 (C=N stretching), 1487 (C=C stretching), 1291 (C–O–C stretching), 1062 (C–S stretching). ^1^H NMR (400 MHz, DMSO-*d*_6_): δ 8.16 (dd, *J* = 5.7, 2.8 Hz, 1H), 7.82–7.74 (m, 2H), 7.54–7.41 (m, 2H), 7.12 (dd, *J* = 5.7, 2.7 Hz, 1H), 4.69 (s, 2H), 3.90 (s, 3H), 3.82 (s, 3H) 2.41 (s, 3H) ppm. ^13^C NMR (100 MHz, DMSO-*d*_6_): δ 165.77, 164.00, 158.61, 148.80, 146.01, 143.32, 139.43, 133.14, 129.82, 127.17, 124.05, 123.46, 109.01, 61.02, 56.53, 34.36, 21.28 ppm. HRMS (ESI-TOF) calcd for C_17_H_17_N_3_O_3_S: [M + H]^+^, 344.1068; found, 344.0628.

##### 4.1.2.7 2-(3-Chlorophenyl)-5-(((3,4-dimethoxypyridin-2-yl)­methyl)­thio)-1,3,4-oxadiazole (**5g**)

Off white solid, mp 136–138 °C, yield = 90%, FTIR (cm^–1^): 3091 (C–H stretching), 1584 and 1554 (C=N stretching), 1483 (C=C stretching), 1296 (C–O–C stretching), 1062 (C–S stretching), 831 (C–Cl stretching). ^1^H NMR (400 MHz, CDCl_3_): δ 8.21 (d, *J* = 5.6 Hz, 1H), 8.01 (t, *J* = 1.9 Hz, 1H), 7.95–7.88 (m, 1H), 7.53–7.47 (m, 1H), 7.44 (t, *J* = 7.8 Hz, 1H), 6.82 (d, *J* = 5.5 Hz, 1H), 4.73 (s, 2H), 3.94 (s, 3H), 3.94 (s, 3H) ppm. ^13^C NMR (100 MHz, CDCl_3_): δ 164.91, 164.66, 158.60, 148.63, 145.74, 143.76, 135.15, 131.61, 130.39, 126.70, 125.34, 124.80, 107.59, 61.12, 55.79, 34.04 ppm. HRMS (ESI-TOF) calcd for C_16_H_14_ClN_3_O_3_S: [M + H]^+^, 364.0522; found, 364.0521.

##### 4.1.2.8 2-(((3,4-Dimethoxypyridin-2-yl)­methyl)­thio)-5-(*o*-tolyl)-1,3,4-oxadiazole (**5h**)

White solid, mp 119–121 °C, yield = 87%, FTIR (cm^–1^): 3085 (C–H stretching), 1615 and 1588 (C=N stretching), 1494 (C=C stretching), 1294 (C–O–C stretching), 1066 (C–S stretching). ^1^H NMR (400 MHz, DMSO-*d*_6_): δ 8.15 (d, *J* = 5.5 Hz, 1H), 7.87 (d, *J* = 7.7 Hz, 1H), 7.51 (t, *J* = 7.4 Hz, 1H), 7.48–7.42 (m, 1H), 7.40 (d, *J* = 7.4 Hz, 1H), 7.11 (d, *J* = 5.5 Hz, 1H), 4.69 (s, 2H), 3.90 (s, 3H), 3.82 (s, 3H), 2.60 (s, 3H) ppm. ^13^C NMR (100 MHz, DMSO-*d*_6_): δ 165.87, 163.68, 158.62, 148.84, 146.01, 143.32, 137.95, 132.19, 131.95, 129.12, 126.97, 122.73, 109.01, 61.02, 56.53, 34.30, 21.83 ppm. HRMS (ESI-TOF) calcd for C_17_H_17_N_3_O_3_S: [M + H]^+^, 344.1068; found, 344.1066.

##### 4.1.2.9 2-(2-Chlorophenyl)-5-(((3,4-dimethoxypyridin-2-yl)­methyl)­thio)-1,3,4-oxadiazole (**5i**)

Off white solid, mp 141–143 °C, yield = 94%, FTIR (cm^–1^): 3157 (C–H stretching), 1588 and 1550 (C=N stretching), 1488 (C=C stretching), 1293 (C–O–C stretching), 1063 (C–S stretching), 824 (C–Cl stretching). ^1^H NMR (400 MHz, DMSO-*d*_6_): δ 8.16 (d, *J* = 5.5 Hz, 1H), 7.97 (dd, *J* = 7.8, 1.7 Hz, 1H), 7.72 (d, *J* = 8.0 Hz, 1H), 7.69–7.61 (m, 1H), 7.57 (t, *J* = 7.6 Hz, 1H), 7.12 (d, *J* = 5.6 Hz, 1H), 4.71 (s, 2H), 3.90 (s, 3H), 3.82 (s, 3H) ppm. ^13^C NMR (100 MHz, DMSO-*d*_6_): δ 164.81, 163.73, 158.62, 148.60, 146.00, 143.30, 133.74, 132.16, 131.67, 131.60, 128.37, 122.73, 109.04, 61.01, 56.54, 34.37 ppm. HRMS (ESI-TOF) calcd for C_16_H_14_ClN_3_O_3_S: [M + H]^+^, 364.0522; found, 364.0528.

##### 4.1.2.10 2-(2,4-Dichlorophenyl)-5-(((3,4-dimethoxypyridin-2-yl)­methyl)­thio)-1,3,4-oxadiazole (**5j**)

Off white solid, mp 145–147 °C, yield = 91%, FTIR (cm^–1^): 3081 (C–H stretching), 1588 and 1564 (C=N stretching), 1487 (C=C stretching), 1296 (C–O–C stretching), 1066 (C–S stretching), 824 (C–Cl stretching). ^1^H NMR (400 MHz, DMSO-*d*_6_): δ 8.16 (d, *J* = 5.6 Hz, 1H), 8.00 (d, *J* = 8.5 Hz, 1H), 7.93 (s, 1H), 7.68 (d, *J* = 6.4 Hz, 1H), 7.12 (d, *J* = 5.6 Hz, 1H), 4.71 (s, 2H), 3.91 (s, 3H), 3.83 (s, 3H) ppm. ^13^C NMR (100 MHz, DMSO-*d*_6_): δ 164.99, 163.06, 158.62, 148.55, 146.01, 143.29, 137.64, 133.22, 132.78, 131.23, 128.70, 121.70, 109.05, 61.01, 56.54, 34.37 ppm. HRMS (ESI-TOF) calcd for C_16_H_13_Cl_2_N_3_O_3_S: [M + H]^+^, 398.0132; found, 398.0135.

##### 4.1.2.11 2-(3,5-Dichlorophenyl)-5-(((3,4-dimethoxypyridin-2-yl)­methyl)­thio)-1,3,4-oxadiazole (**5k**)

White solid, mp 130–132 °C, yield = 90%, FTIR (cm^–1^): 3073 (C–H stretching), 1588 and 1544 (C=N stretching), 1544 (C=C stretching), 1179 (C–O–C stretching), 1065 (C–S stretching), 825 (C–Cl stretching). ^1^H NMR (400 MHz, DMSO-*d*_6_): δ 8.16 (d, *J* = 5.5 Hz, 1H), 7.98 (d, *J* = 2.0 Hz, 2H), 7.93 (d, *J* = 2.3 Hz, 1H), 7.12 (d, *J* = 5.6 Hz, 1H), 4.72 (s, 2H), 3.90 (s, 3H), 3.83 (s, 3H) ppm. ^13^C NMR (100 MHz, DMSO-*d*_6_): δ 165.25, 163.54, 158.62, 148.66, 146.00, 143.35, 135.69, 131.77, 126.67, 125.32, 109.04, 61.04, 56.54, 34.31 ppm. HRMS (ESI-TOF) calcd for C_16_H_13_Cl_2_N_3_O_3_S: [M + H]^+^, 398.0132; found, 398.0139.

##### 4.1.2.12 2-(4-(Benzyloxy)­phenyl)-5-(((3,4-dimethoxypyridin-2-yl)­methyl)­thio)-1,3,4-oxadiazole (**5L**)

White solid, mp 149–151 °C, yield = 89%, FTIR (cm^–1^): 3077 (C–H stretching), 1617 and 1582 (C=N stretching), 1582 (C=C stretching), 1291 (C–O–C stretching), 1060 (C–S stretching). ^1^H NMR (400 MHz, DMSO-*d*_6_): δ 8.15 (d, *J* = 5.5 Hz, 1H), 7.92 (d, *J* = 9.2 Hz, 2H), 7.52–7.31 (m, 5H), 7.22 (d, *J* = 8.6 Hz, 2H), 7.11 (d, *J* = 5.5 Hz, 1H), 5.22 (s, 2H), 4.66 (s, 2H), 3.89 (s, 3H), 3.81 (s, 3H) ppm. ^13^C NMR (100 MHz, DMSO-*d*_6_): δ 165.62, 163.28, 161.61, 158.61, 148.88, 146.01, 143.33, 136.92, 128.99, 128.76, 128.52, 128.32, 116.16, 116.09, 109.00, 70.01, 61.02, 56.53, 34.37 ppm. HRMS (ESI-TOF) calcd for C_23_H_21_Cl_2_N_3_O_4_S: [M + H]^+^, 506.0710; found, 506.0708.

### 4.2 Single Crystal XRD

Crystals of compound **5f** were grown via slow evaporation using methanol. Colorless crystals appeared after several days, and a representative crystal of C_17_H_17_N_3_O_3_S, (0.152 mm × 0.165 mm × 0.189 mm) was selected for X-ray analysis. Data collection was performed using a Bruker D8 QUEST diffractometer with X-ray radiation (λ = 0.71073 Å). The total exposure time was 0.62 h. Using the Bruker SAINT software package with a narrow-frame algorithm, 39,963 reflections were integrated, reaching a maximum θ angle of 28.42° (0.75 Å resolution). Of these, 4165 were independent (completeness = 99.2%, *R*_int_ = 9.38%, *R*_sig_ = 6.45%), and 2251 (54.05%) had intensities greater than 2σ­(*F* ^2^). Absorption effects were corrected using the multi-scan method (SADABS), yielding a minimum-to-maximum apparent transmission ratio of 0.872.

### 4.3 Cytotoxicity Assay

The 3-[4,5-dimethylthiazol-2-yl]-2,5-diphenyltetrazolium bromide (MTT) assay was employed to assess the cytotoxic effects of the test compounds on A549 lung cancer cells.^37–40^ The MTT assay is a colorimetric technique based on the enzymatic reduction of the yellow tetrazolium salt (MTT) into insoluble purple formazan crystals by mitochondrial succinate dehydrogenase, an enzyme involved in the electron transport chain of metabolically active cells.^41^

A549 cells were plated at a density of 10^4^ cells per well in 96-well plates containing DMEM supplemented with 10% FBS. The plates were placed in a humidified incubator at 37 °C with 5% CO_2_ for 24 h to allow cell adhesion. After incubation, the cells were washed with phosphate-buffered saline (PBS, pH 7.4) before treatment. The test compounds, dissolved in dimethyl sulfoxide (DMSO) and diluted in serum-deprived (1% FBS) DMEM to a final concentration of 10 μM, were added to the wells. The cells were then incubated with the test compounds for 72 h.

At the end of the incubation period, the medium was removed, and the cells were washed with PBS. Subsequently, 100 μL of MTT solution (1 mg/mL in PBS) was added to each well, and the plates were incubated in the dark for 4 h to facilitate the reduction of MTT to formazan crystals by viable cells. The resulting formazan crystals were solubilized by adding 100 μL of DMSO per well, and absorbance was measured at 570 nm using a microplate reader to determine cell viability.^42^ The IC_50_ of the most active compound was determined by treating the cells with serial concentrations of the test compound (100 μM, 50 μM, 10 μM, 1 μM, and 0.5 μM).^43–45^

### 4.4 ADME Analysis

The ADME values of the synthesized derivatives were predicted using the QikProp module in the Maestro software.

### 4.5 In-Silico Docking Analysis

Ligand–protein interactions were investigated through molecular docking using Schrödinger Maestro. The target protein (PDB ID: 2XMY) was extracted from the RCSB Protein Data Bank.^25–34^ Protein preparation was carried out using the Protein Preparation Wizard to ensure structural integrity. Missing loops and side chains were reconstructed with Prime, while nonessential chains were removed, retaining only the cocrystallized ligand-containing chain. Ligands were sketched in 2D and optimized with LigPrep. A grid box was generated around the cocrystal site, followed by ligand docking.

### 4.6 Molecular Dynamics

MD simulations were conducted for the most active compound **5k** identified in the cytotoxicity assay using the Desmond package in Schrödinger. The protein–ligand complex was enclosed within an orthorhombic simulation box (10 Å × 10 Å × 10 Å) using the TIP3P solvent model. System neutrality was ensured by adding Na^+^ and Cl^–^ ions along with 0.15 M NaCl. Energy minimization was performed using the steepest descent method. The 100 ns simulation was performed under *NPT* conditions, with the Nosé–Hoover chain thermostat maintaining a temperature of 300 K and the Martyna–Tobias–Klein barostat regulating pressure at 1.01 bar. Electrostatic interactions were evaluated using the smooth particle mesh Ewald method.^46–48^

## Supplementary Material



## Notes

General experimental procedures for synthesis of intermediates; spectral data (FTIR, ^1^H NMR, ^13^C NMR, HRMS) of all pyridine-based 1,3,4-oxadiazole hybrids, SCXRD; Hirshfeld surface analysis; molecular docking and dynamics–related figures and tables (PDF)
