# Supplementary material for: Unraveling Structural and Anticancer Properties of Pyridine-Oxadiazole Derivatives: Single-Crystal XRD, Hirshfeld Analysis, and Cytotoxicity against A549 Cells
Source: ACS Omega. 2025 Jun 1;10(22):23549–62. doi: 10.1021/acsomega.5c02152 (PMC12163846; doi:10.1021/acsomega.5c02152)
Supplement: Supplementary file 1 [file ao5c02152_si_001.pdf]

Unraveling Structural and Anticancer Properties of Pyridine-Oxadiazole Derivatives: Single-Crystal XRD, Hirshfeld Analysis, and Cytotoxicity Against A549 Cells

Yogeesha N. Nayak[1], Deepika Dwarakanath[1], Keerthana Suresh Kizhakkanoodan[2], Rajeev K. Sinha[3], K. Sreedhara Ranganath Pai[4], Bharath Raja Guru[2], Santosh L. Gaonkar[1*]

[1]Department of Chemistry, Manipal Institute of Technology, Manipal Academy of Higher Education (MAHE), Manipal, 576104, Karnataka, India.

[2]Department of Biotechnology, Manipal Institute of Technology (MIT), Manipal Academy of Higher Education (MAHE), Manipal, 576104, Karnataka, India

[3]Department of Physics, Birla Institute of Technology Mesra, Ranchi 835215, India

[4]Department of Pharmacology, Manipal College of Pharmaceutical Sciences, Manipal Academy of Higher Education (MAHE), Manipal, 576104, Karnataka, India.

[*]Corresponding author: sl.gaonkar@manipal.edu

Chemistry

1] Synthesis of intermediates

Methods for the preparation of aromatic esters (**2a–l**)

Various aromatic acids (**1a–l**) were esterified with ethanol (10 mL) in the presence of a few drops of concentrated H_2SO_4. This reaction yielded the corresponding esters. The progress of the reaction was monitored using TLC (ethyl acetate: toluene, 1:2). After completion, the reaction mixture was cooled and extracted with ethyl acetate following washing with a sodium bicarbonate solution. The collected organic layer was evaporated to afford ester **2a–l**.

Methods for the preparation of aromatic acid hydrazides (**3a–l**)

The aromatic esters **2a–l** were refluxed with hydrazine hydrate in ethanol for approximately 6 to 12 hours. The progress and completion of the reaction were monitored using TLC (ethyl acetate: toluene, 1:1). After cooling the reaction mixture in an ice bath, the resulting solid was filtered and washed with water, yielding the corresponding hydrazide derivatives **3a–l**.

Methods for the preparation of thio-oxadiazoles (**4a–l**)

Aromatic hydrazides **3a–l** were refluxed with carbon disulfide in ethanol. The progress and completion of the reaction were monitored using TLC (ethyl acetate: toluene, 1:1). After completion of the reaction, the reaction mixture was cooled in an ice bath, and dilute HCl was added with stirring. The resulting solid was filtered and recrystallized from methanol to obtain thio-oxadiazoles **4a–l**. (1–3)

2] Spectral Data (FTIR, NMR, and HRMS)

FTIR Spectra of Pyridine Based 1,3,4-Oxadiazole Derivatives

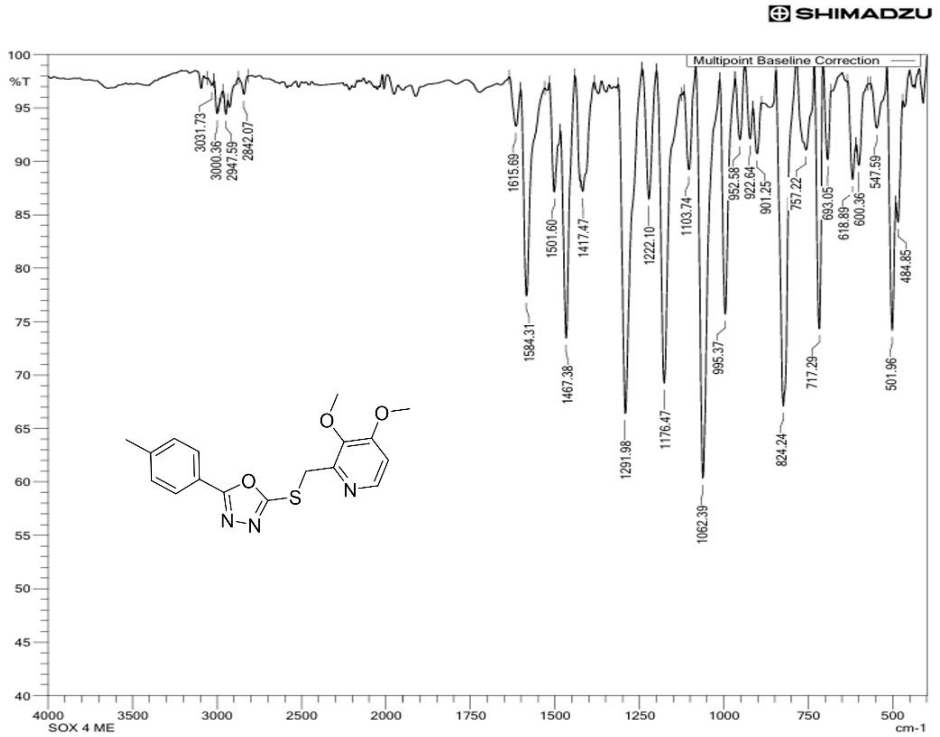

Figure S1: IR spectrum of compound **5a**

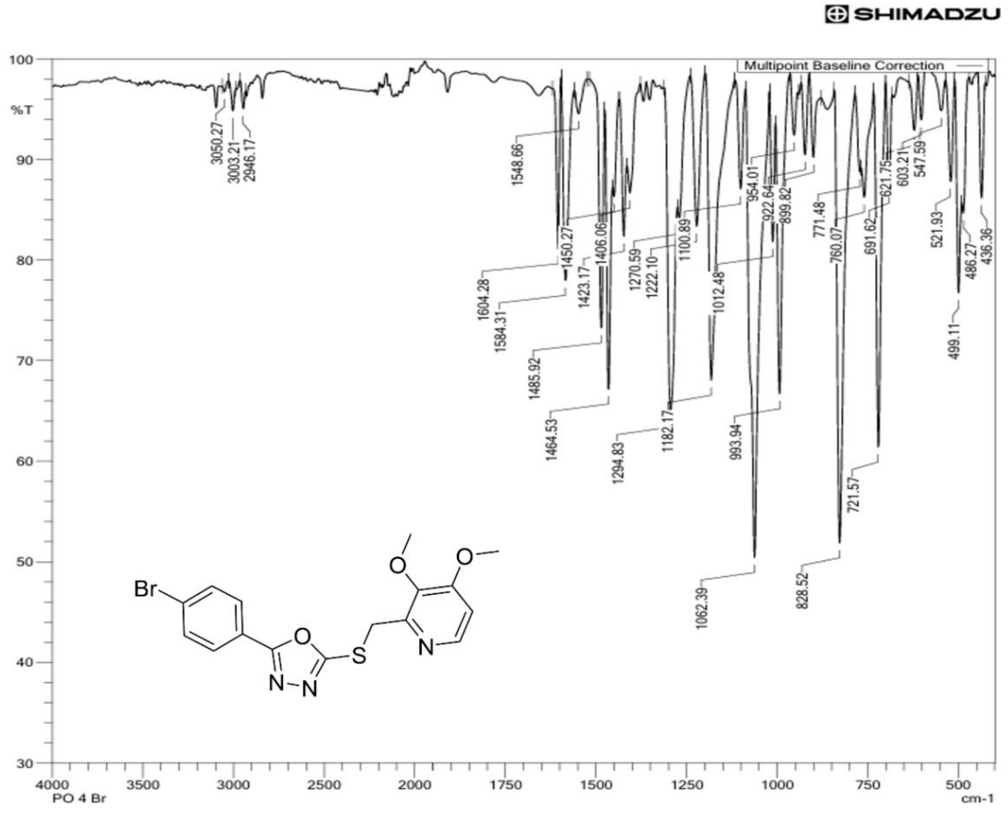

Figure S2: IR spectrum of compound **5b**

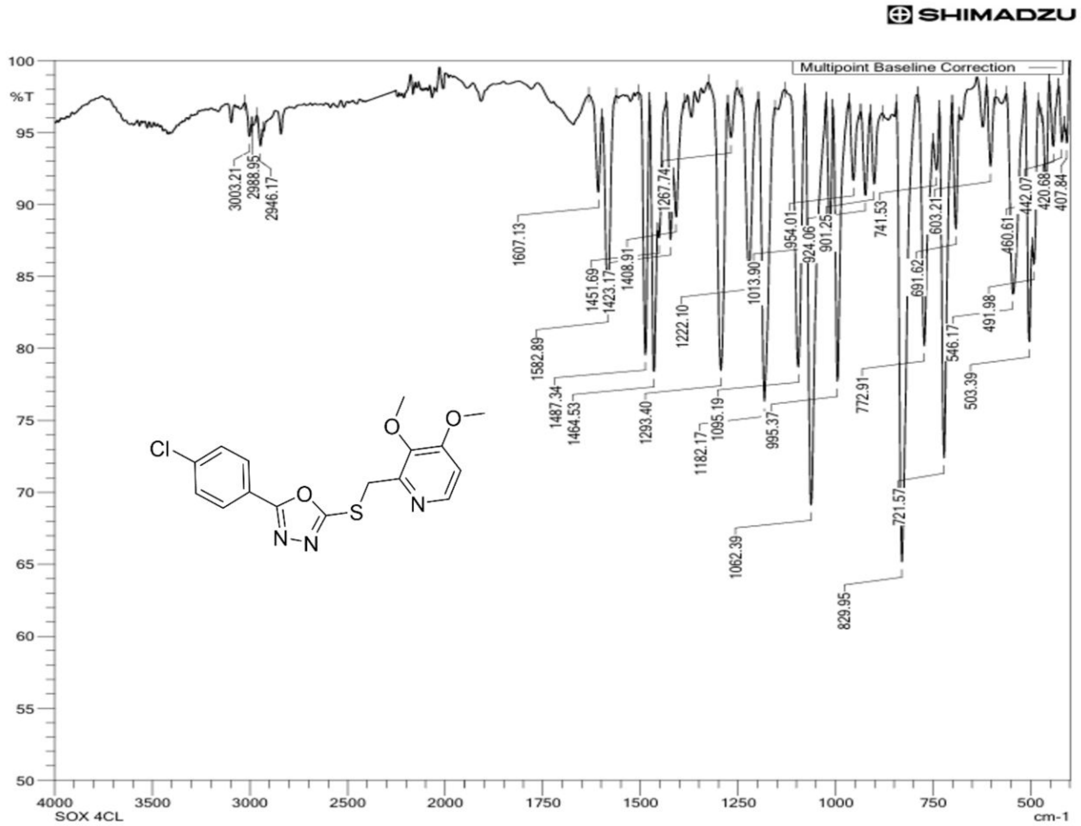

Figure S3: IR spectrum of compound **5c**

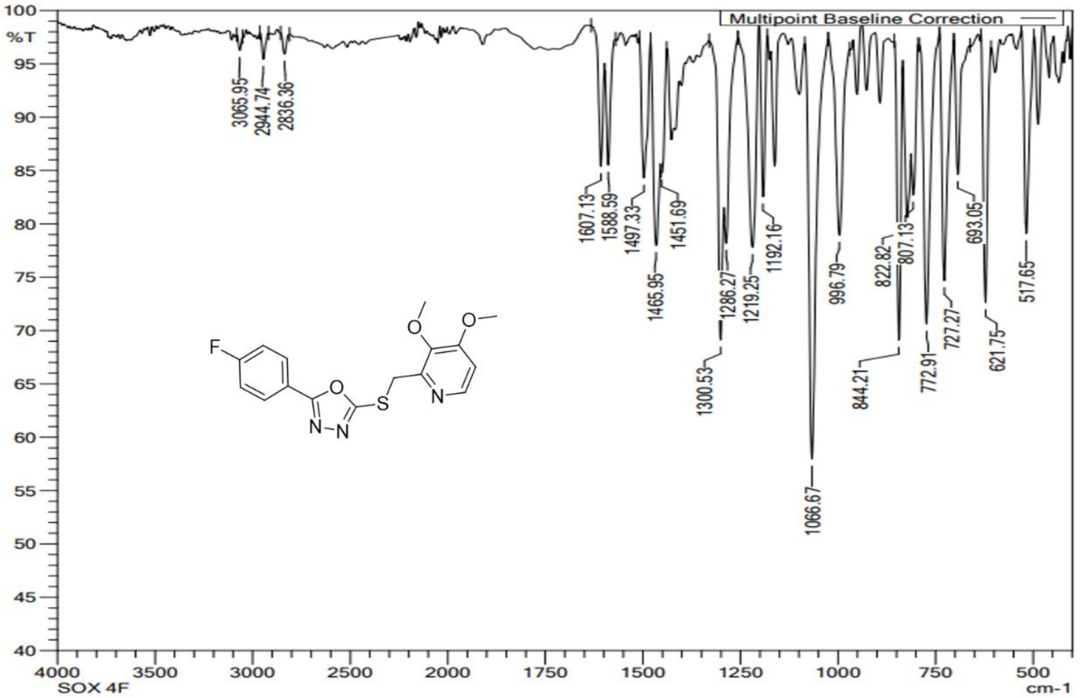

Figure S4: IR spectrum of compound **5d**

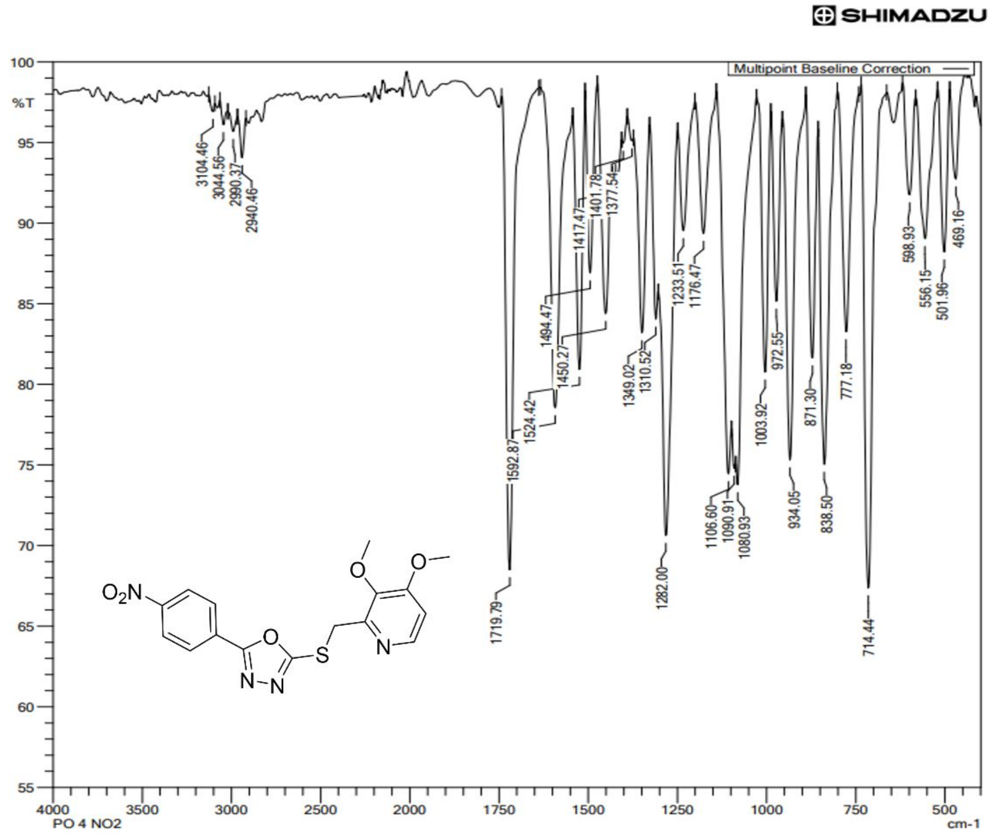

Figure S5: IR spectrum of compound **5e**

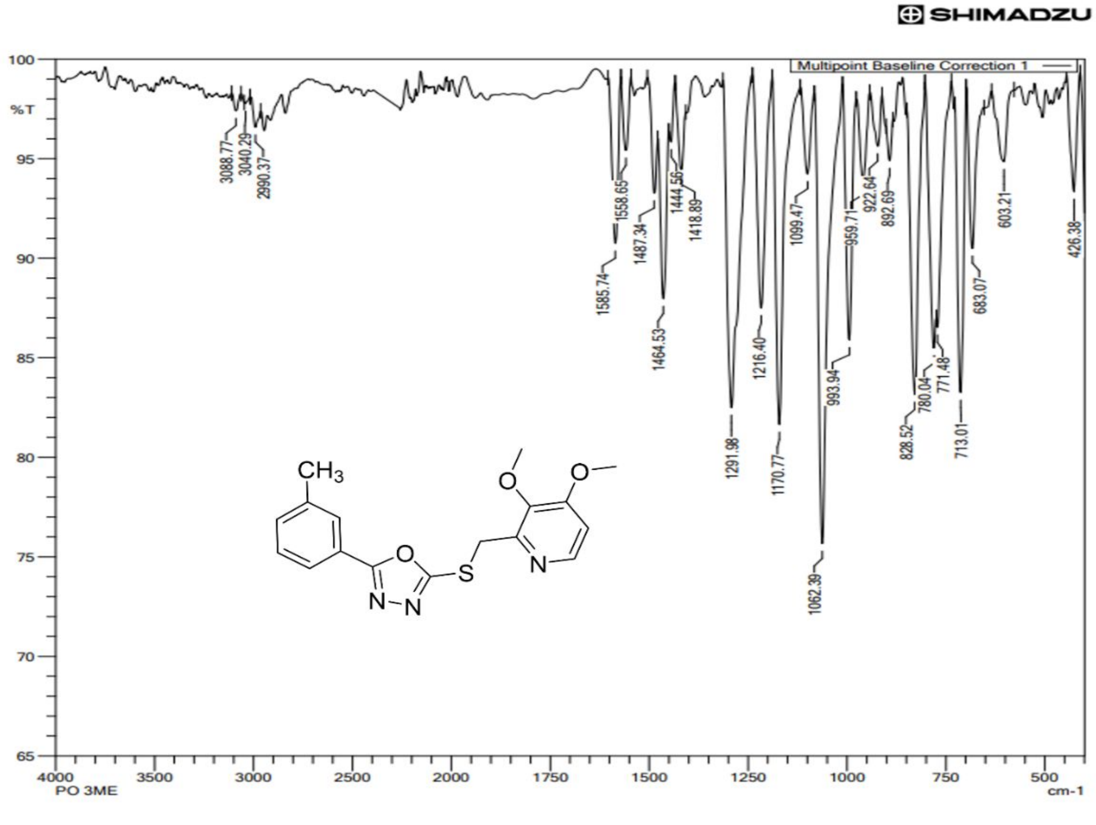

Figure S6: IR spectrum of compound **5f**

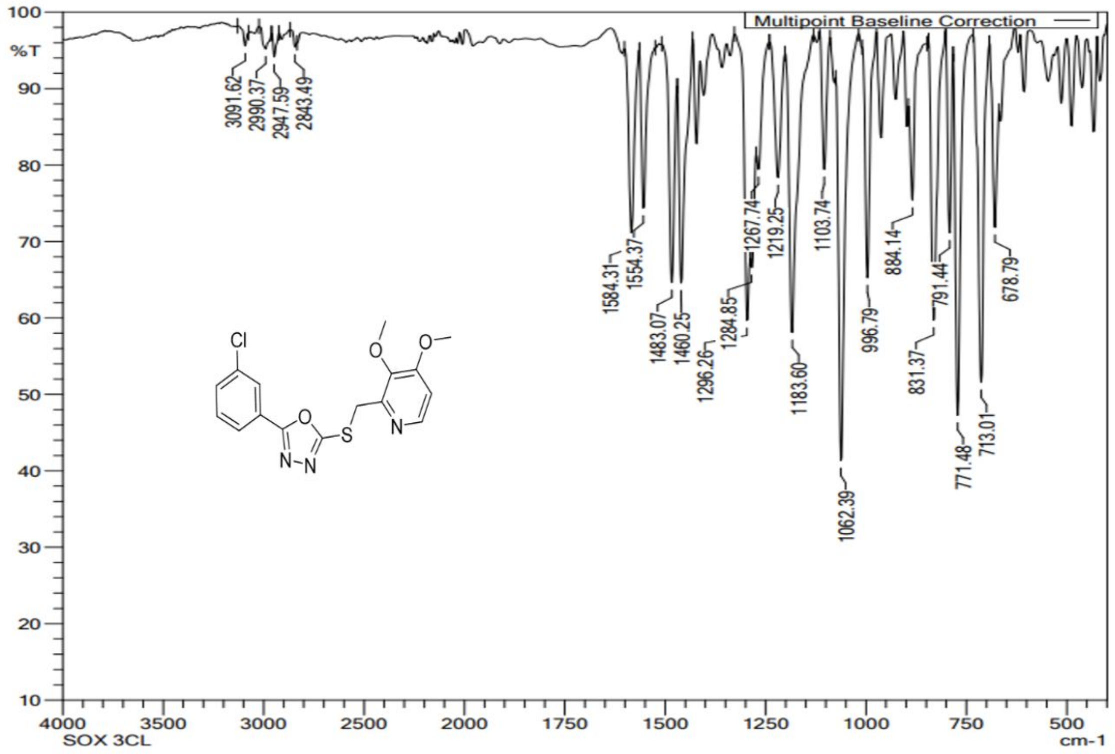

Figure S7: IR spectrum of compound **5g**

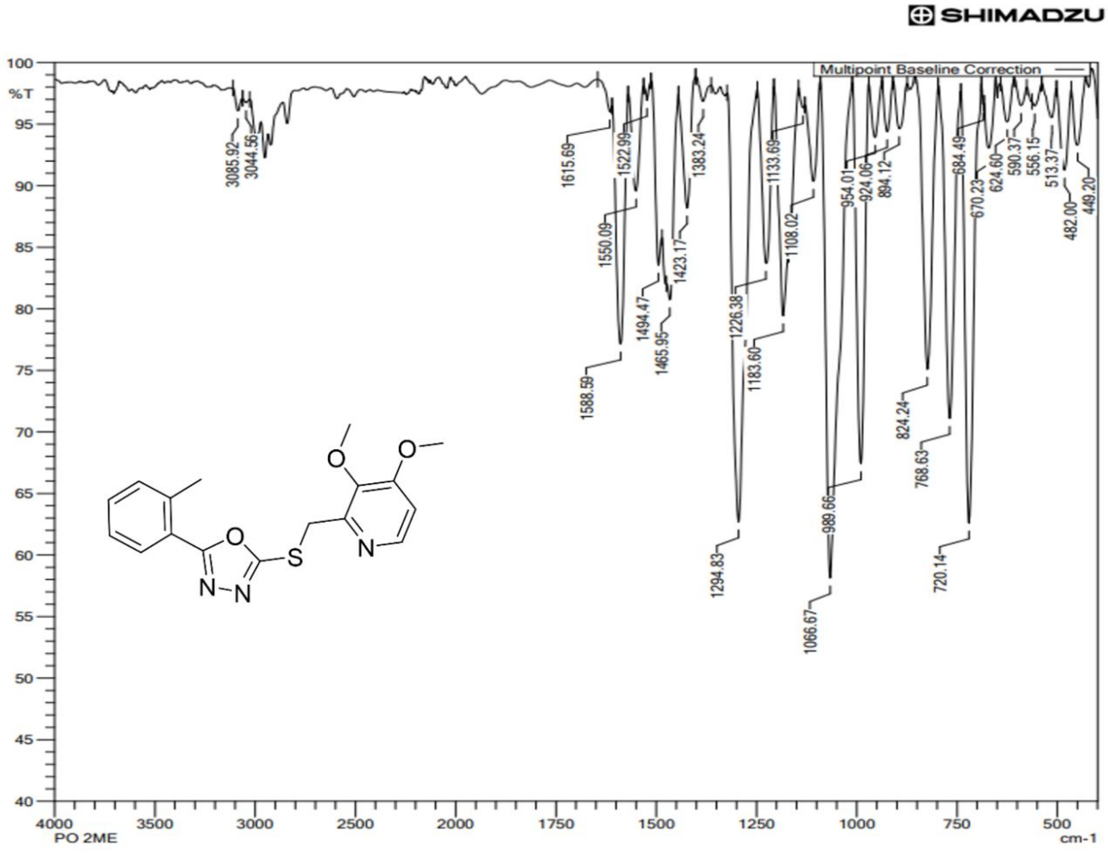

Figure S8: IR spectrum of compound **5h**

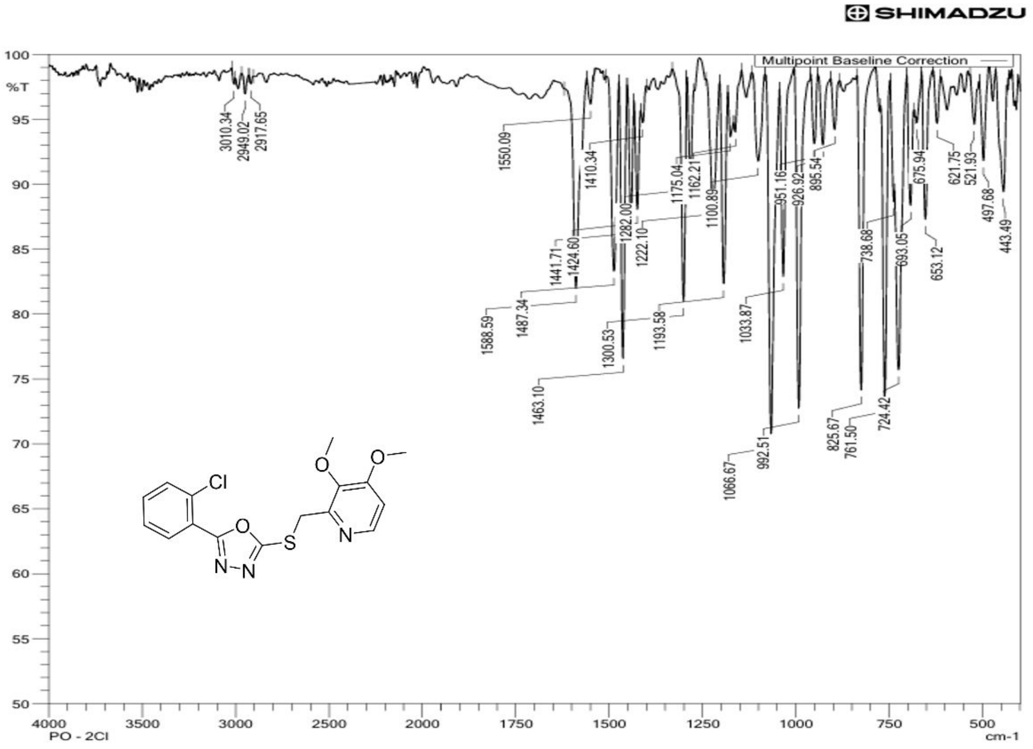

Figure S9: IR spectrum of compound **5i**

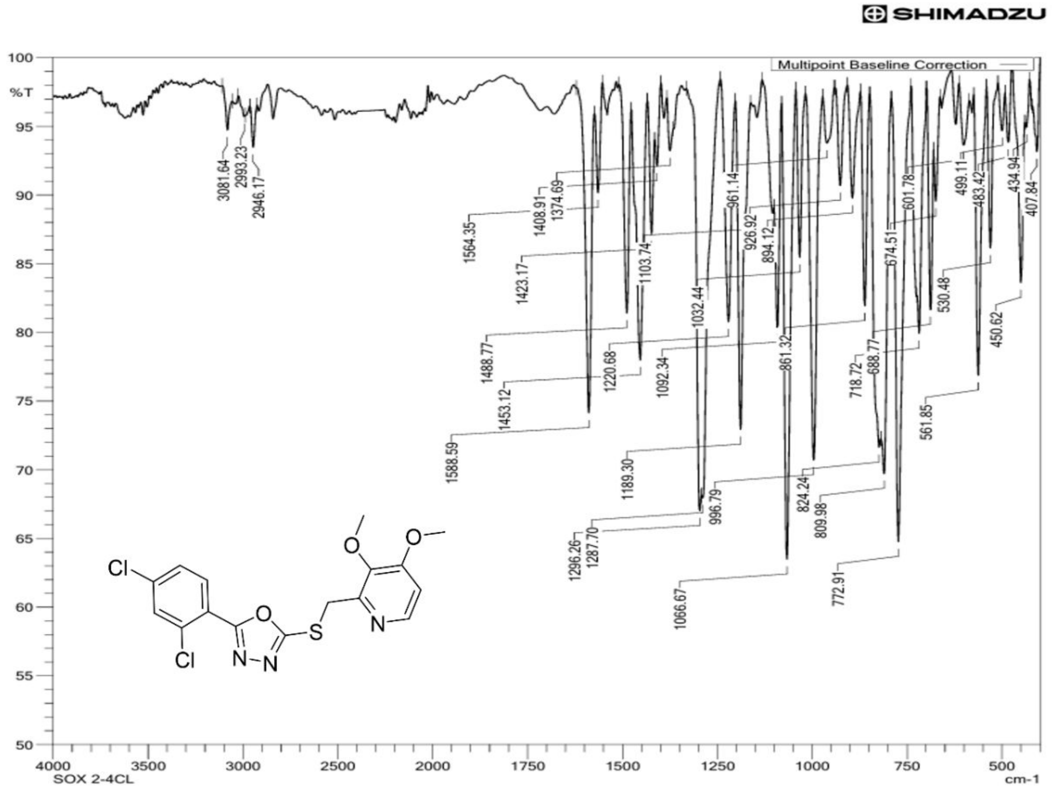

Figure S10: IR spectrum of compound **5j**

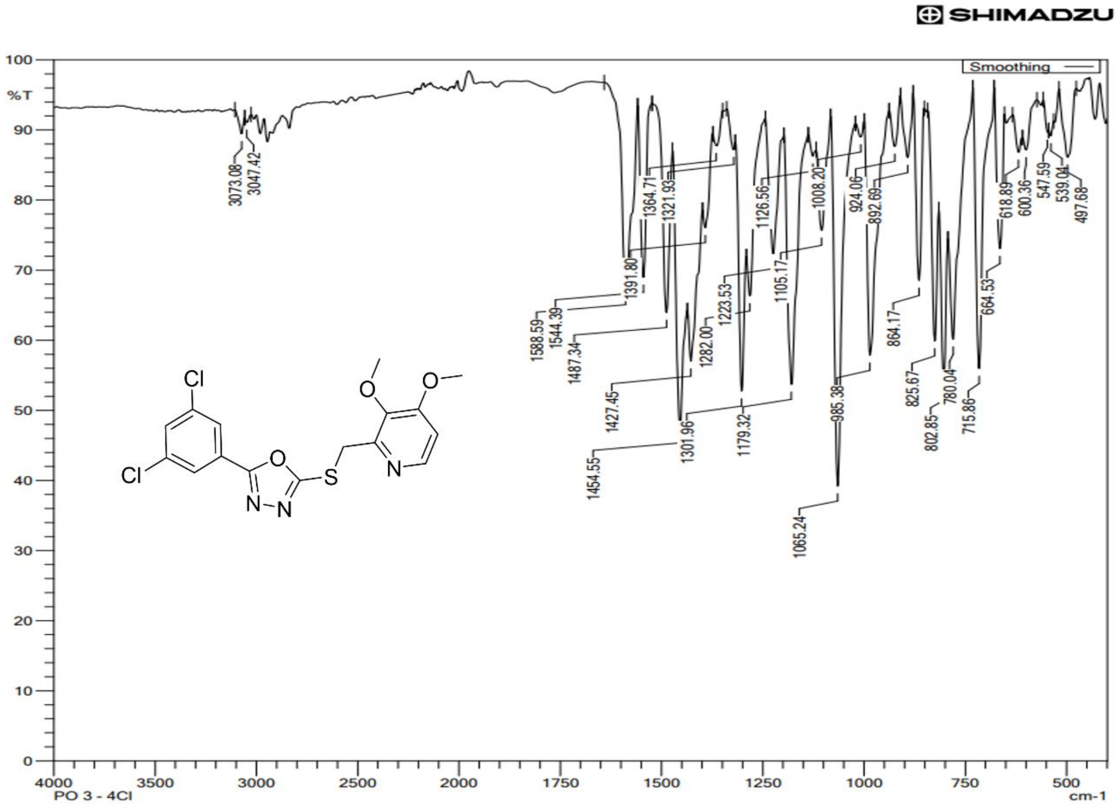

Figure S11: IR spectrum of compound **5k**

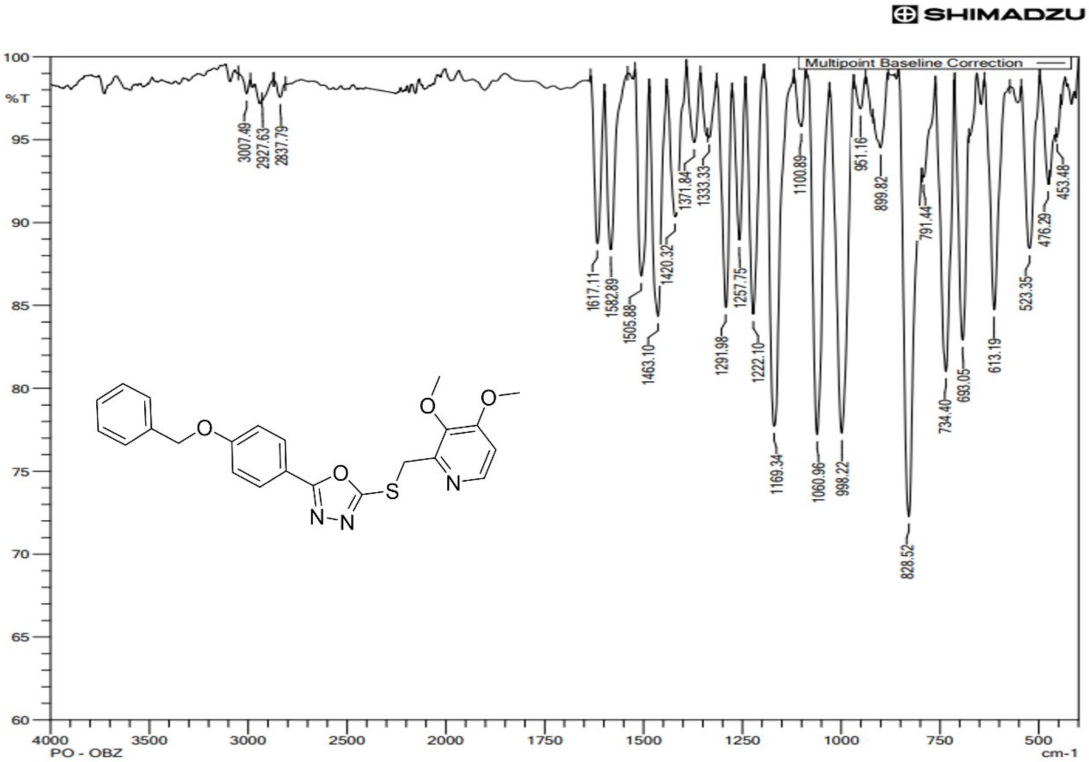

Figure S12: IR spectrum of compound **5l**

^{1}H-NMR Spectra of Pyridine Based 1,3,4-Oxadiazole Derivatives (5a–l)

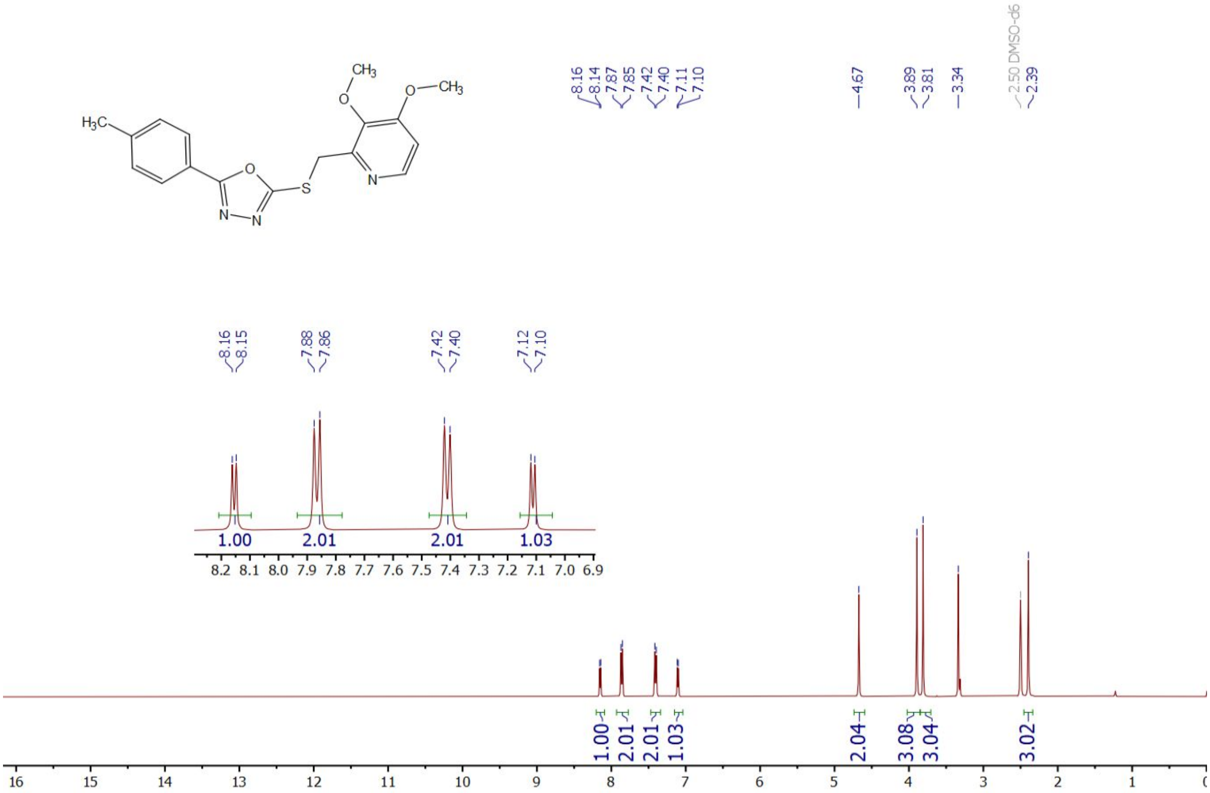

Figure S13: ^{1}H-NMR (400 MHz, DMSO-d_6) spectrum of compound **5a**

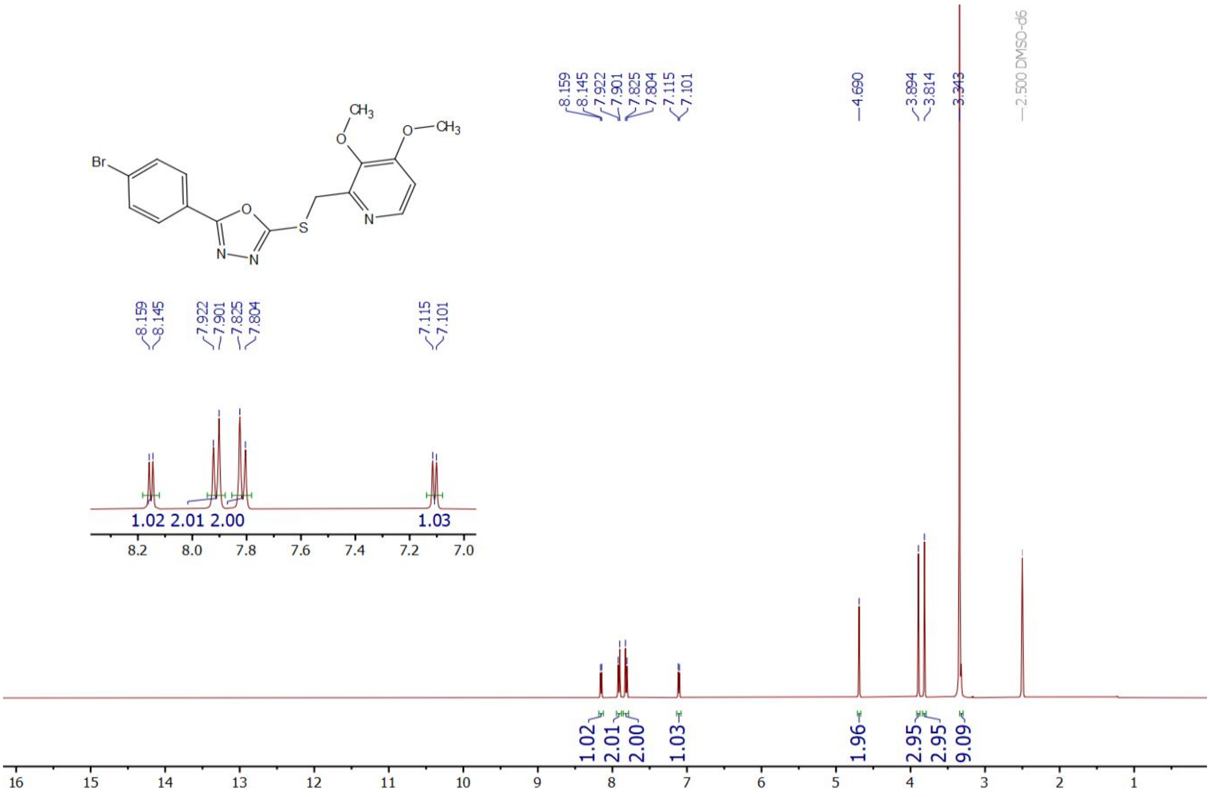

Figure S14: ^{1}H-NMR (400 MHz, DMSO-d_6) spectrum of compound **5b**

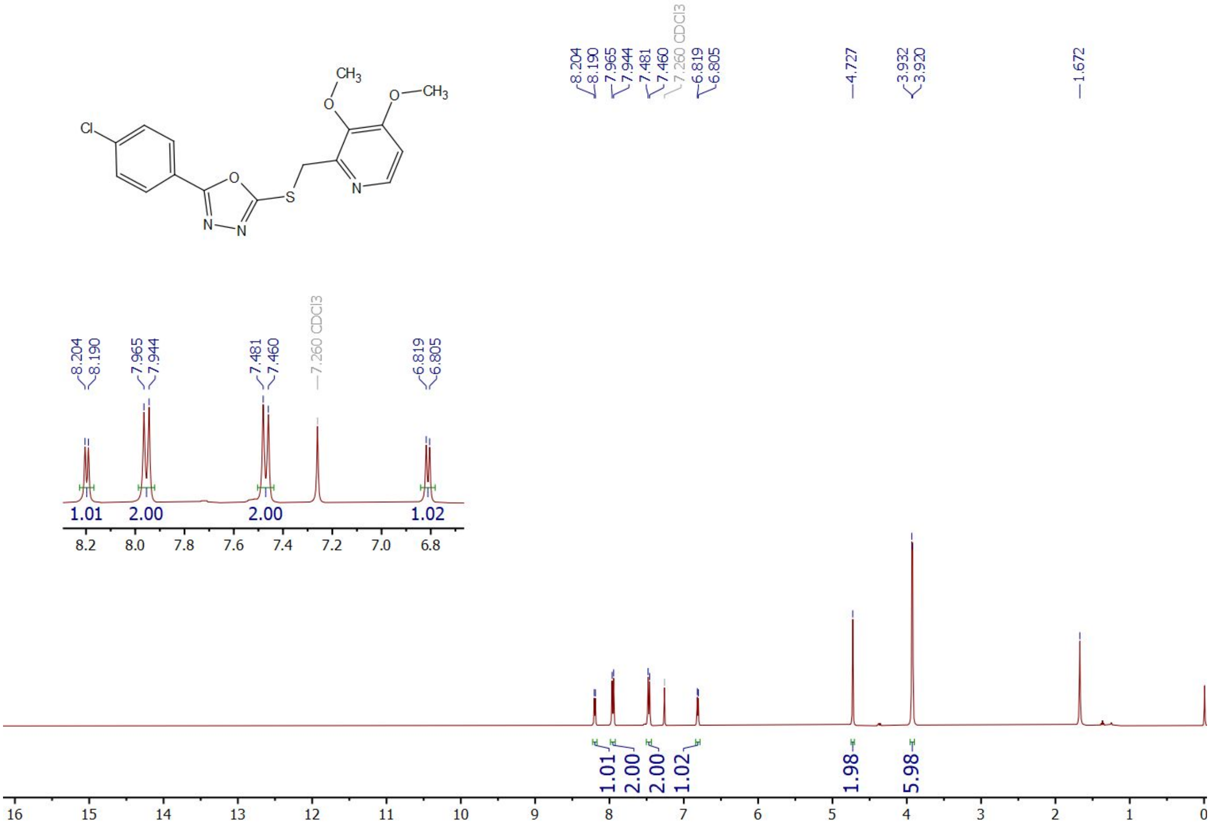

Figure S15: ^{1}H-NMR (400 MHz, $CDCl_3$) spectrum of compound **5c**

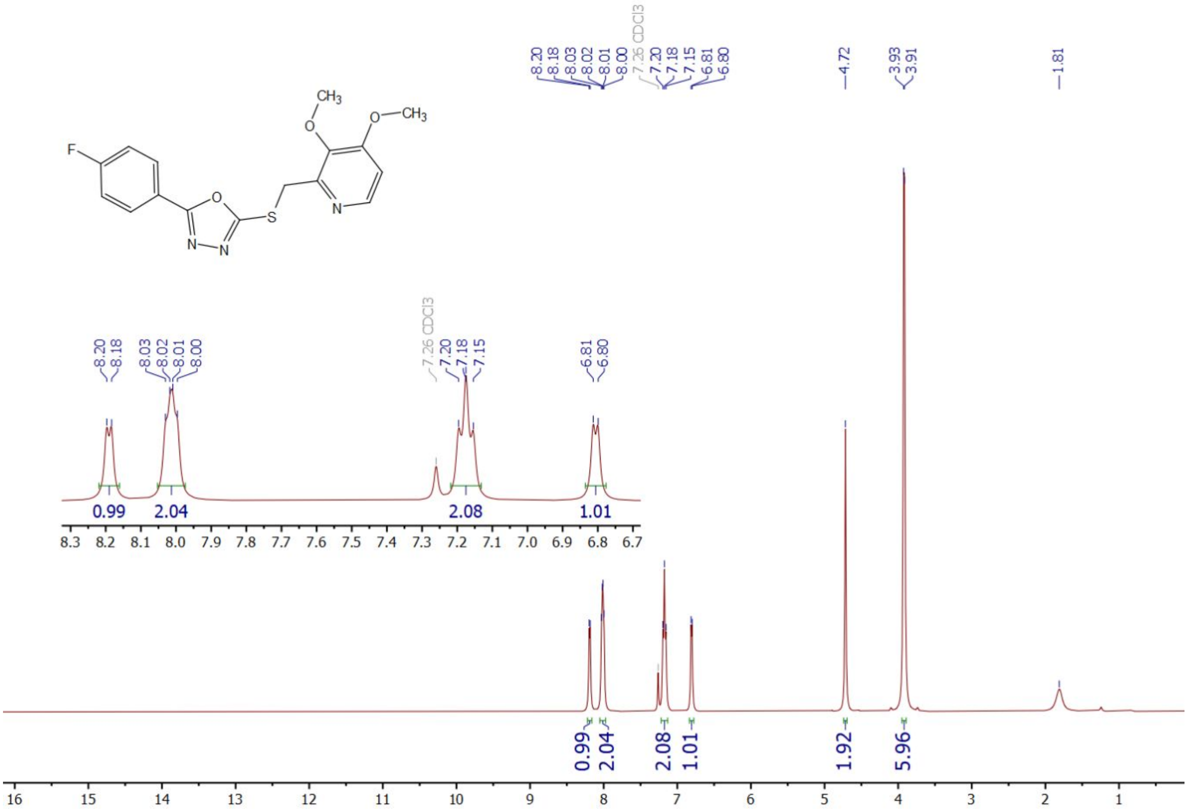

Figure S16: ^{1}H-NMR (400 MHz, $CDCl_3$) spectrum of compound **5d**

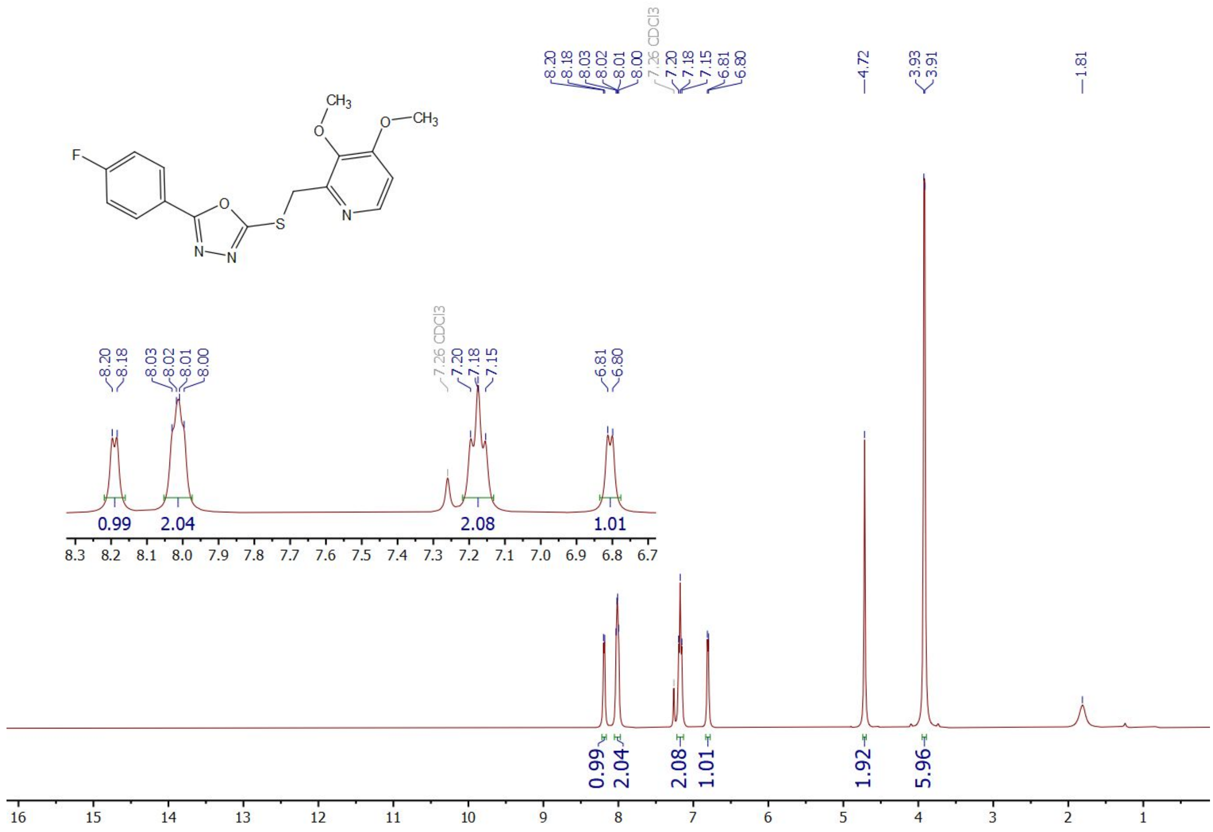

Figure S17: ^{1}H-NMR (400 MHz, DMSO-d$_6$) spectrum of compound **5e**

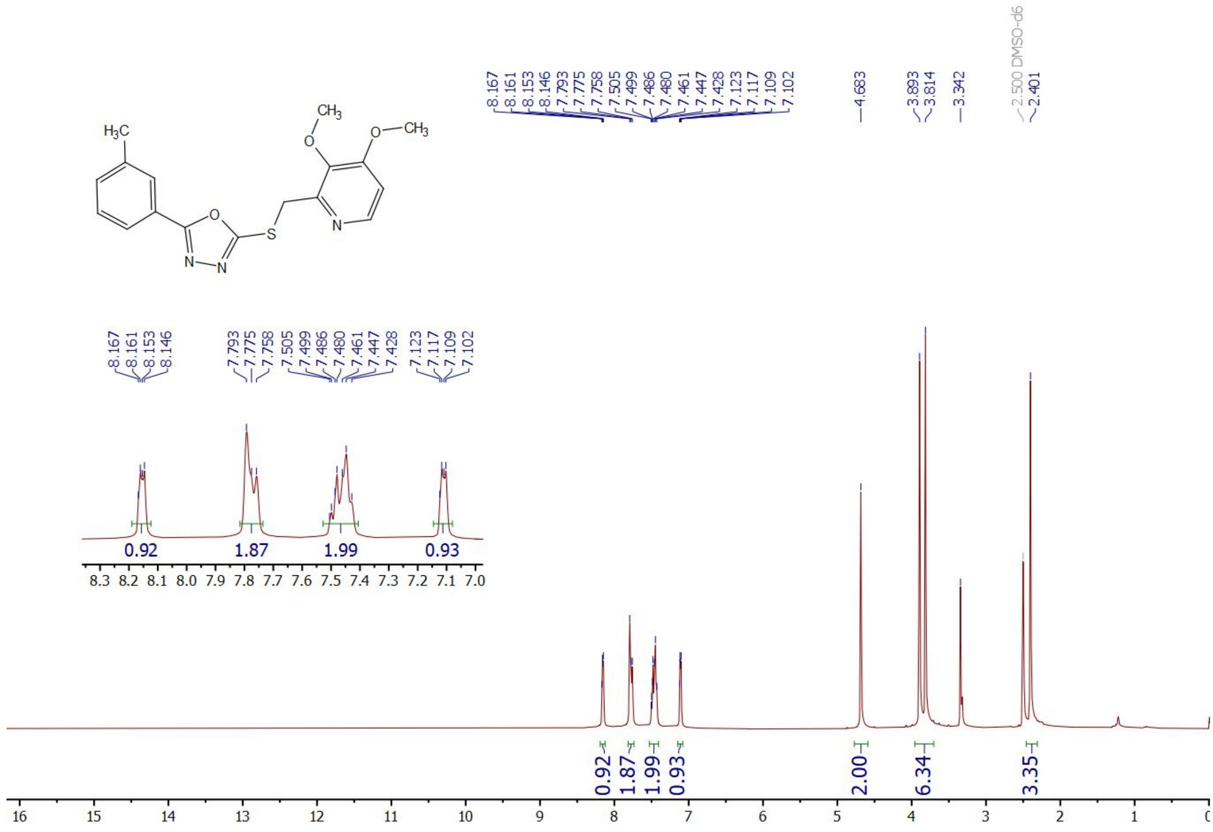

Figure S18: ^{1}H-NMR (400 MHz, DMSO-d6) spectrum of compound **5f**

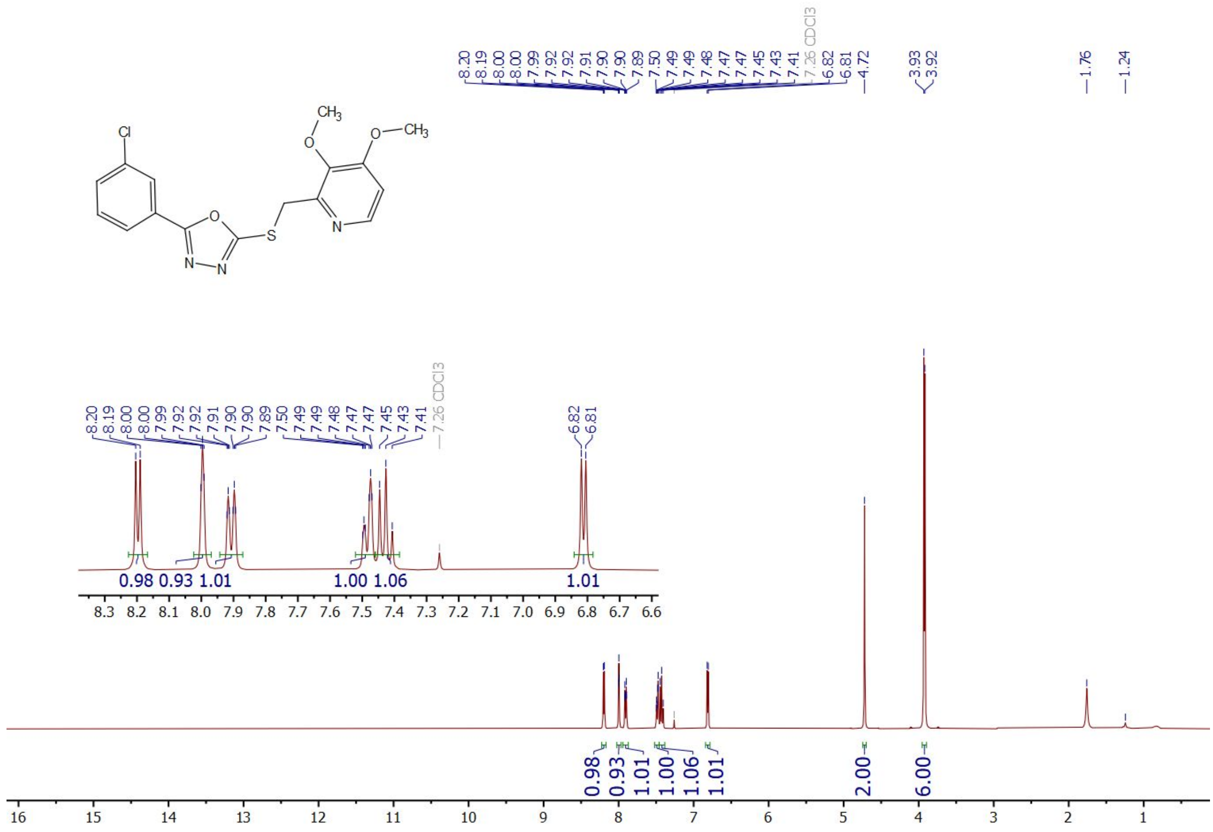

Figure S19: ^{1}H-NMR (400 MHz, DMSO-d$_6$) spectrum of compound **5g**

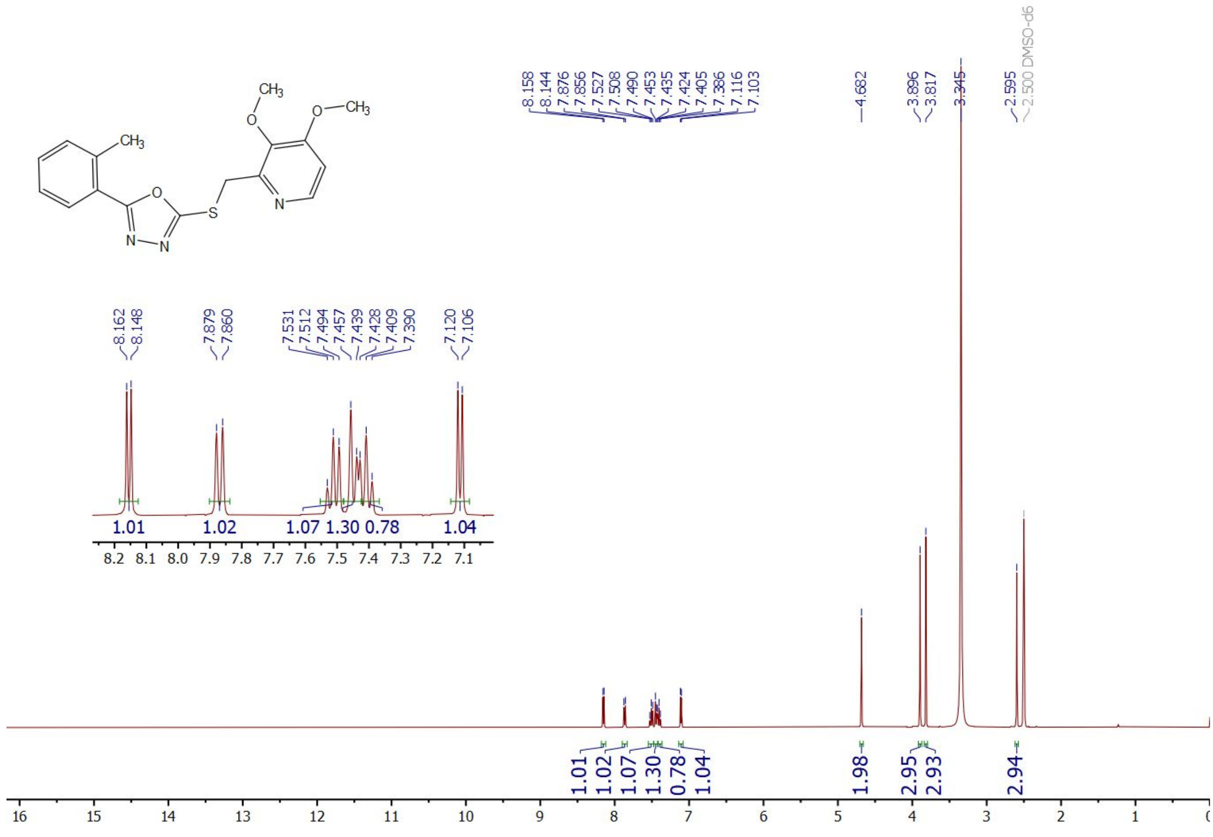

Figure S20: ^{1}H-NMR (400 MHz, DMSO-d$_6$) spectrum of compound **5h**

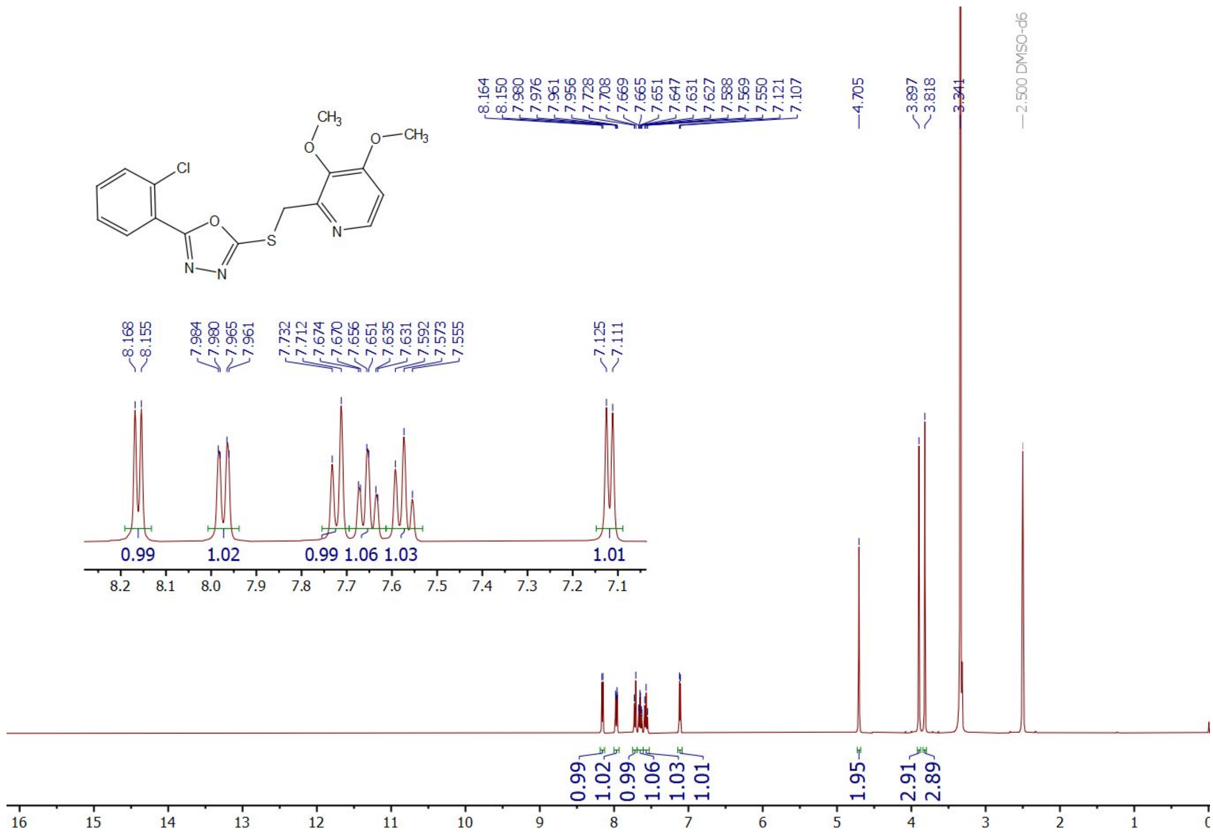

Figure S21: ^{1}H-NMR (400 MHz, DMSO-d$_6$) spectrum of compound **5i**

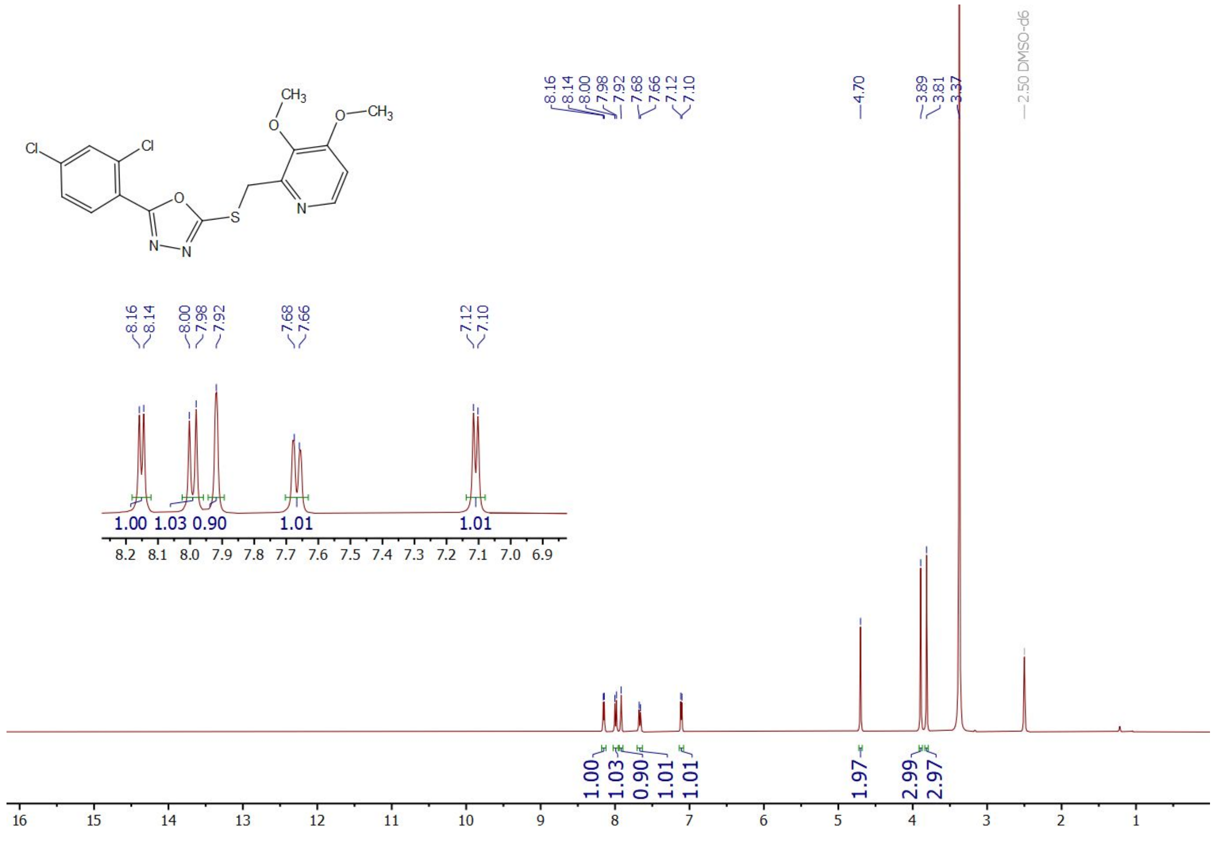

Figure 22: ^{1}H-NMR (400 MHz, DMSO-d$_6$) spectrum of compound **5j**

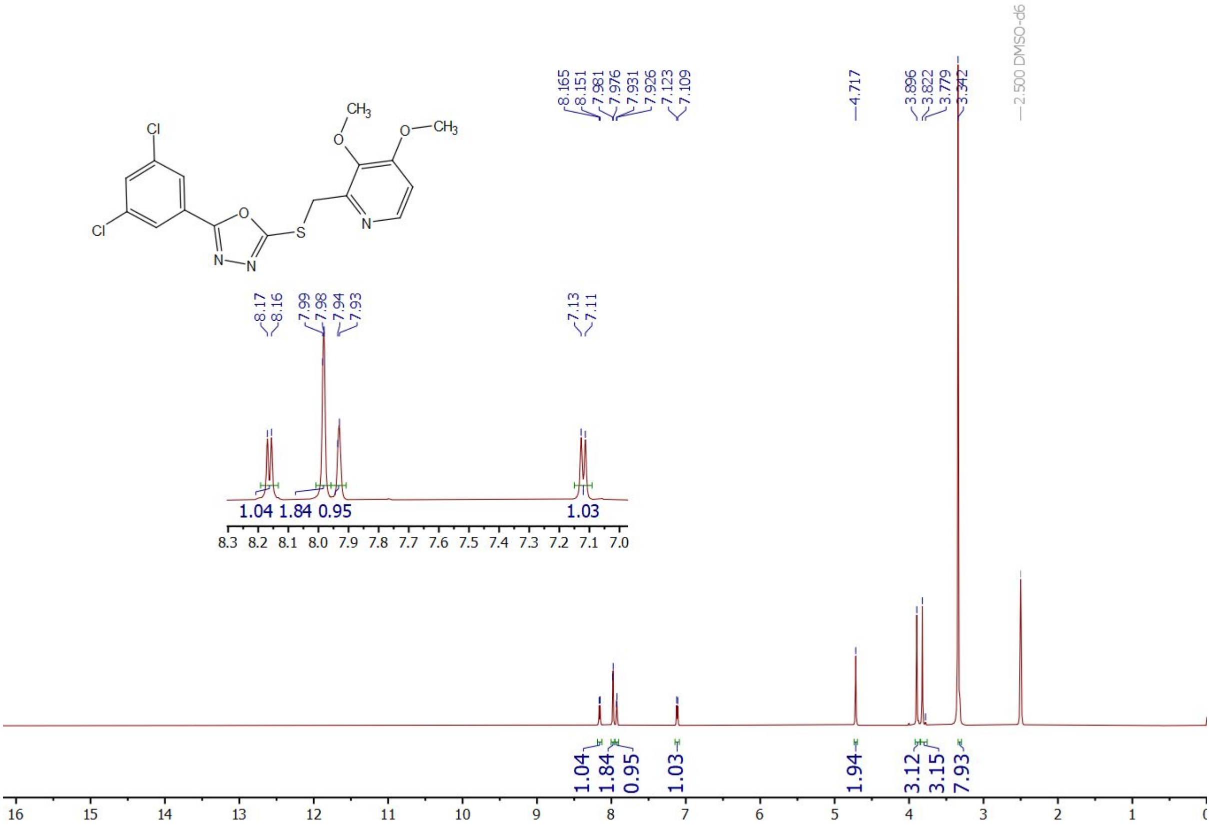

Figure S23: ^{1}H-NMR (400 MHz, DMSO-d_6) spectrum of compound **5k**

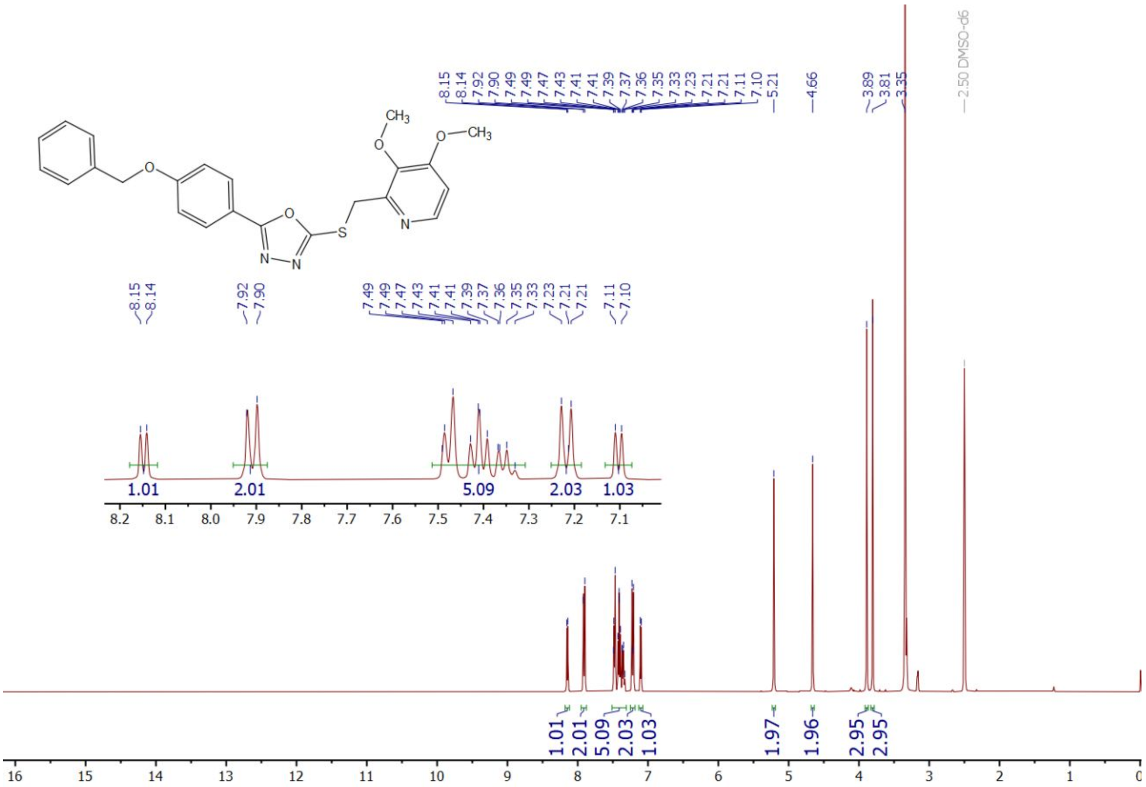

Figure S24: ^{1}H-NMR (400 MHz, DMSO-d_6) spectrum of compound **5l**

^{13}C-NMR Spectra of Pyridine Based 1,3,4-Oxadiazole Derivatives (5a–l)

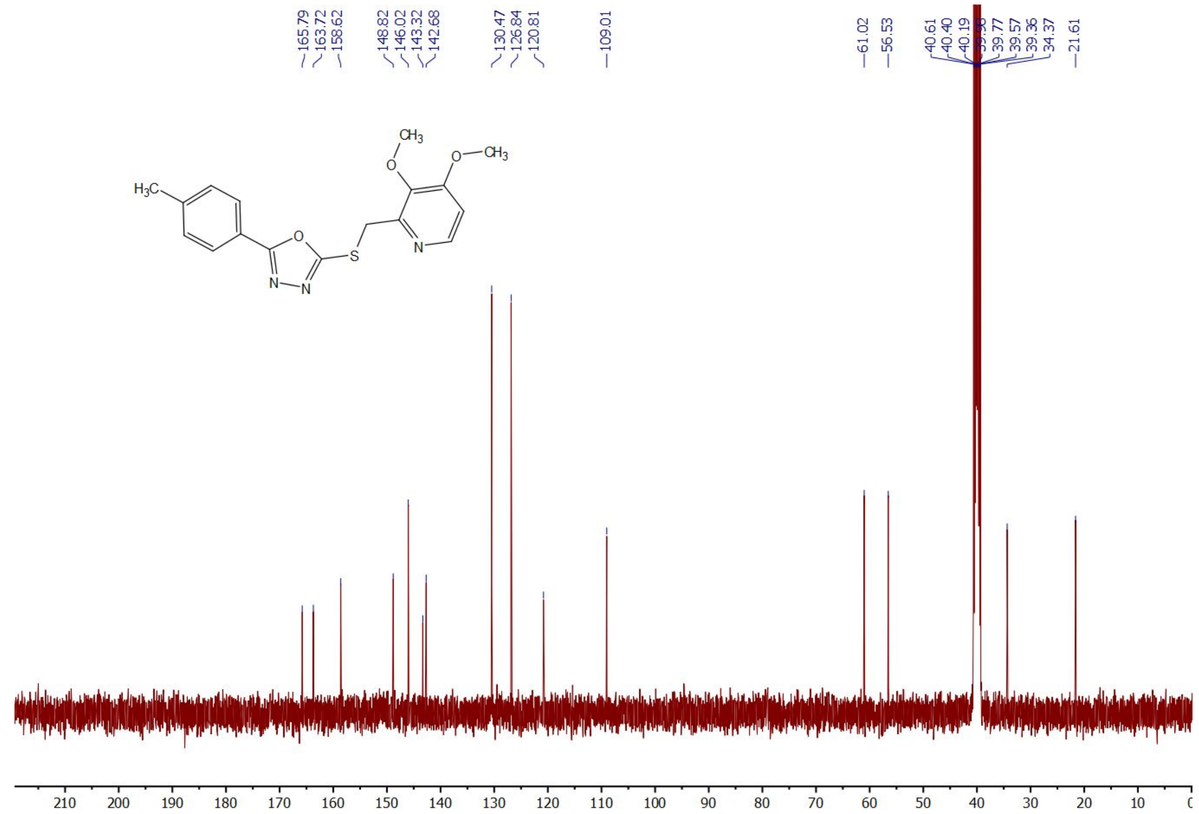

Figure S25: ^{13}C-NMR (100 MHz, DMSO-d_6) spectrum of compound **5a**

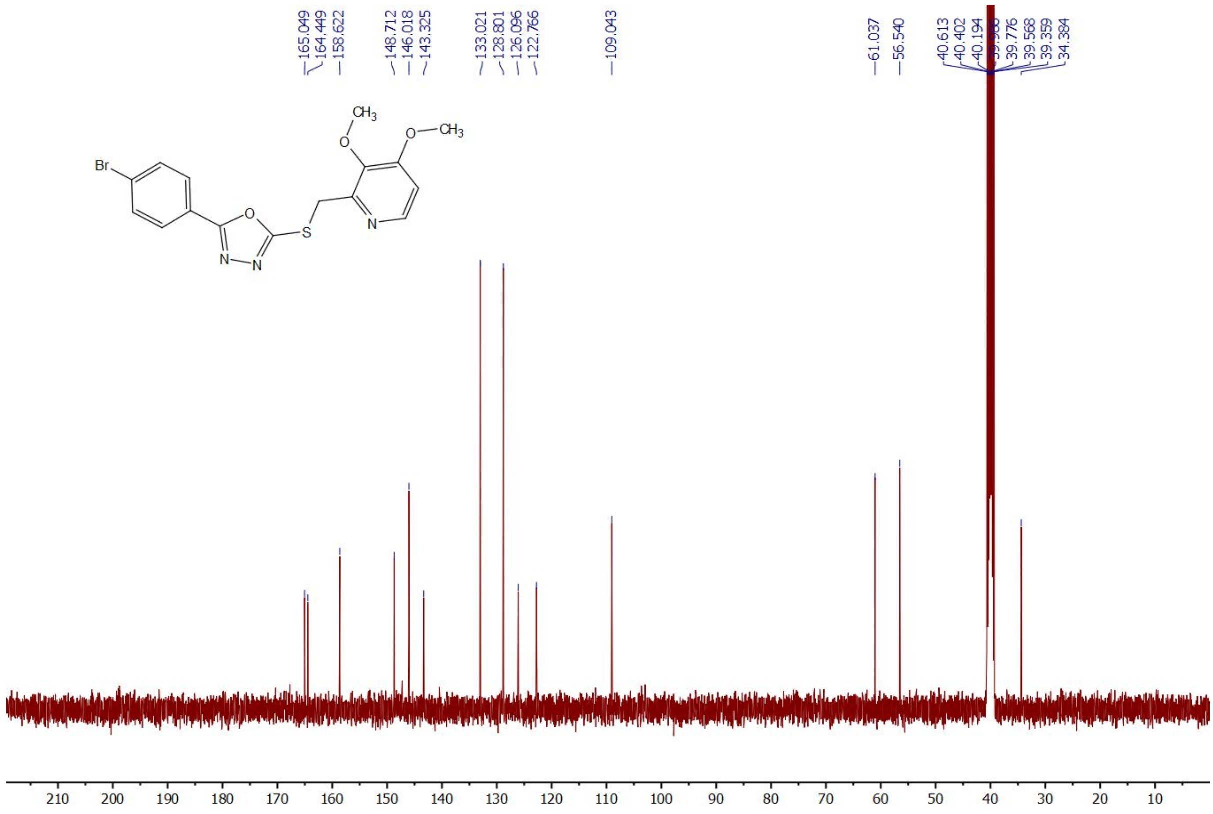

Figure S26: ^{13}C-NMR (100 MHz, DMSO-d_6) spectrum of compound **5b**

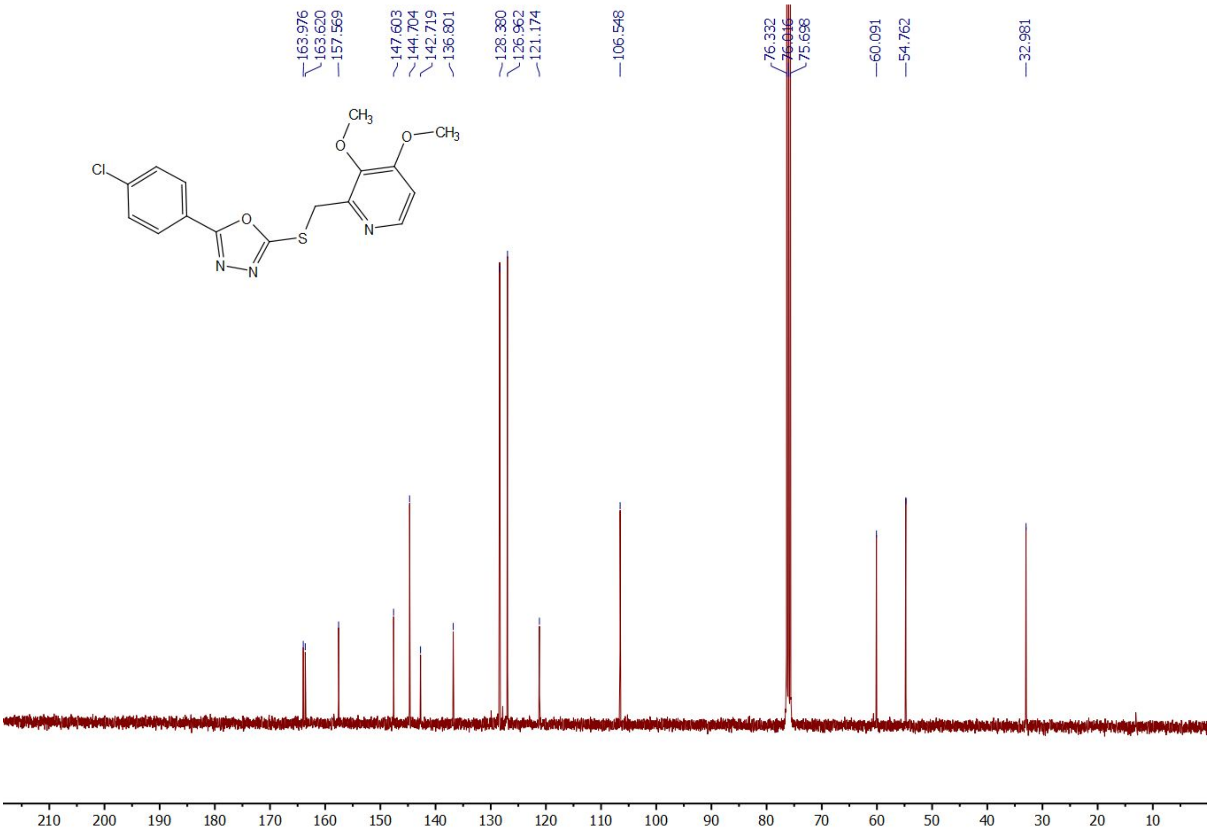

Figure S27: ^{13}C-NMR (100 MHz, $CDCl_3$) spectrum of compound **5c**

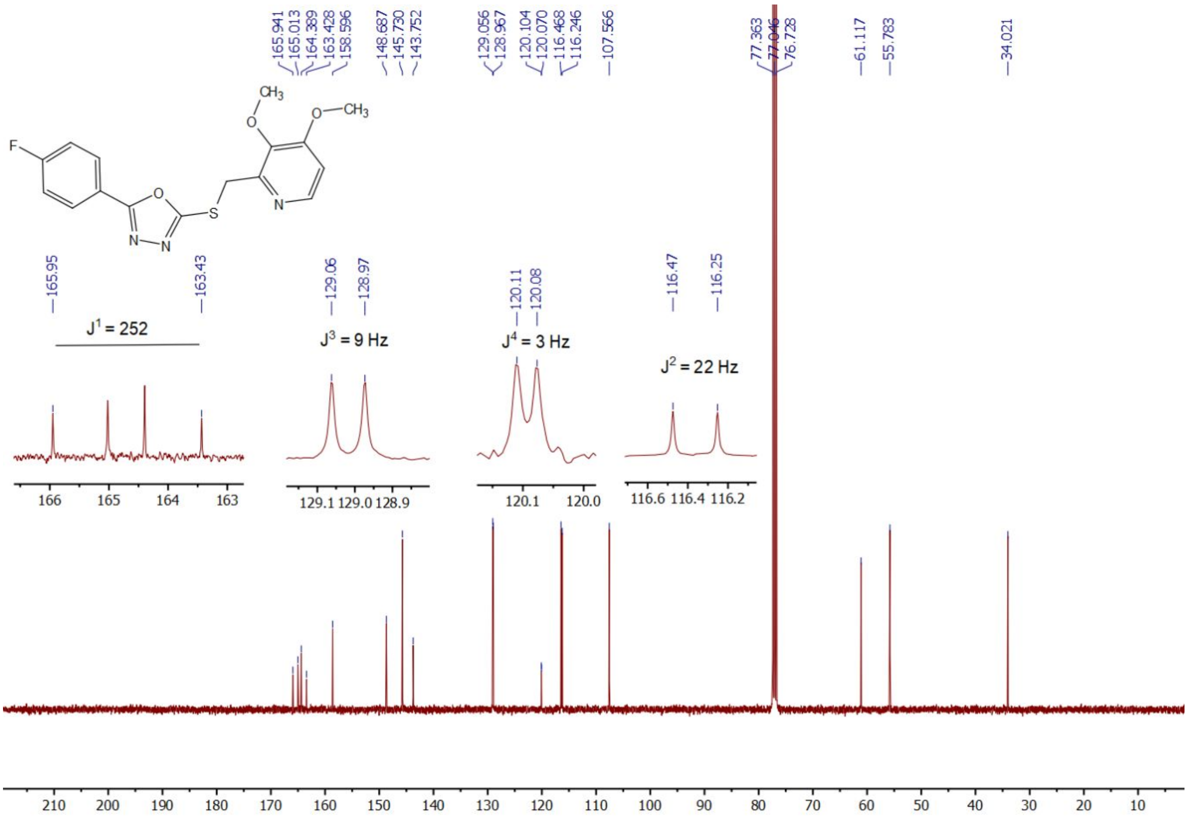

Figure S28: ^{13}C-NMR (100 MHz, $CDCl_3$) spectrum of compound **5d**

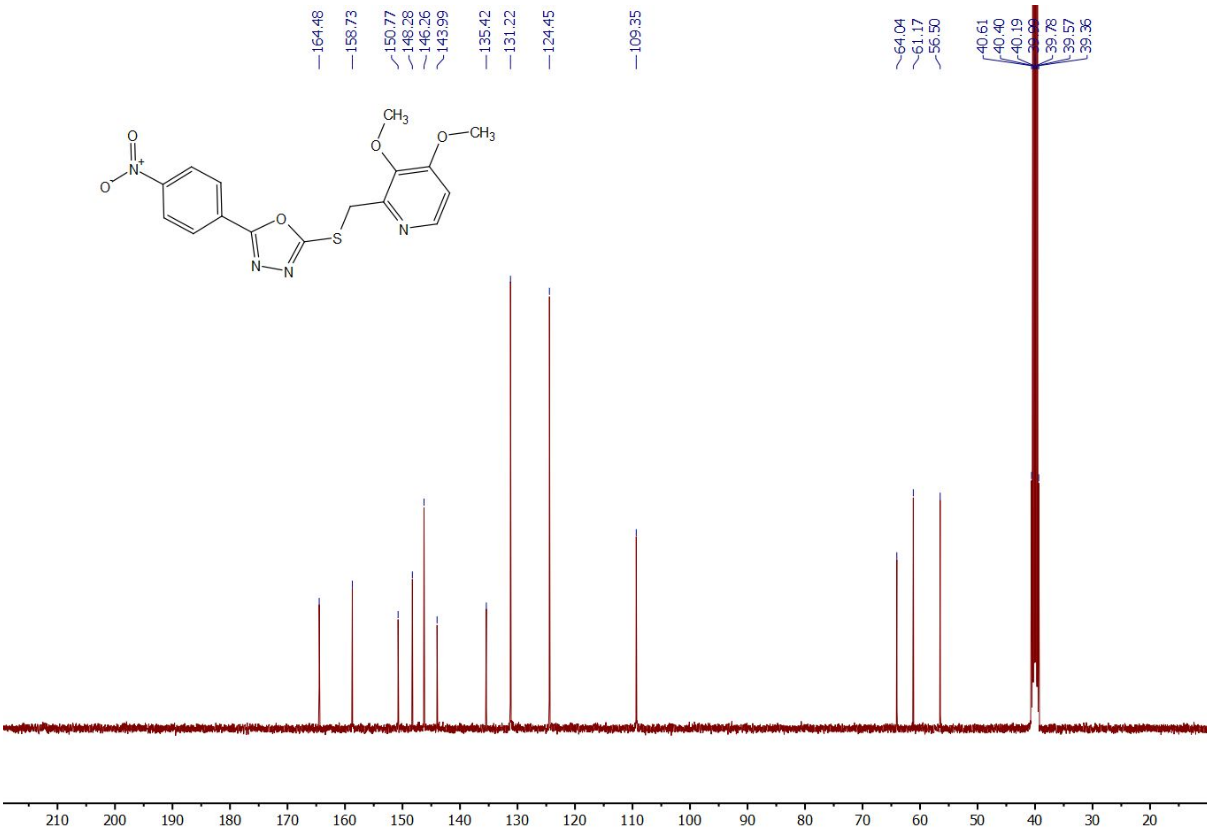

Figure S29: ^{13}C-NMR (100 MHz, DMSO-d$_6$) spectrum of compound **5e**

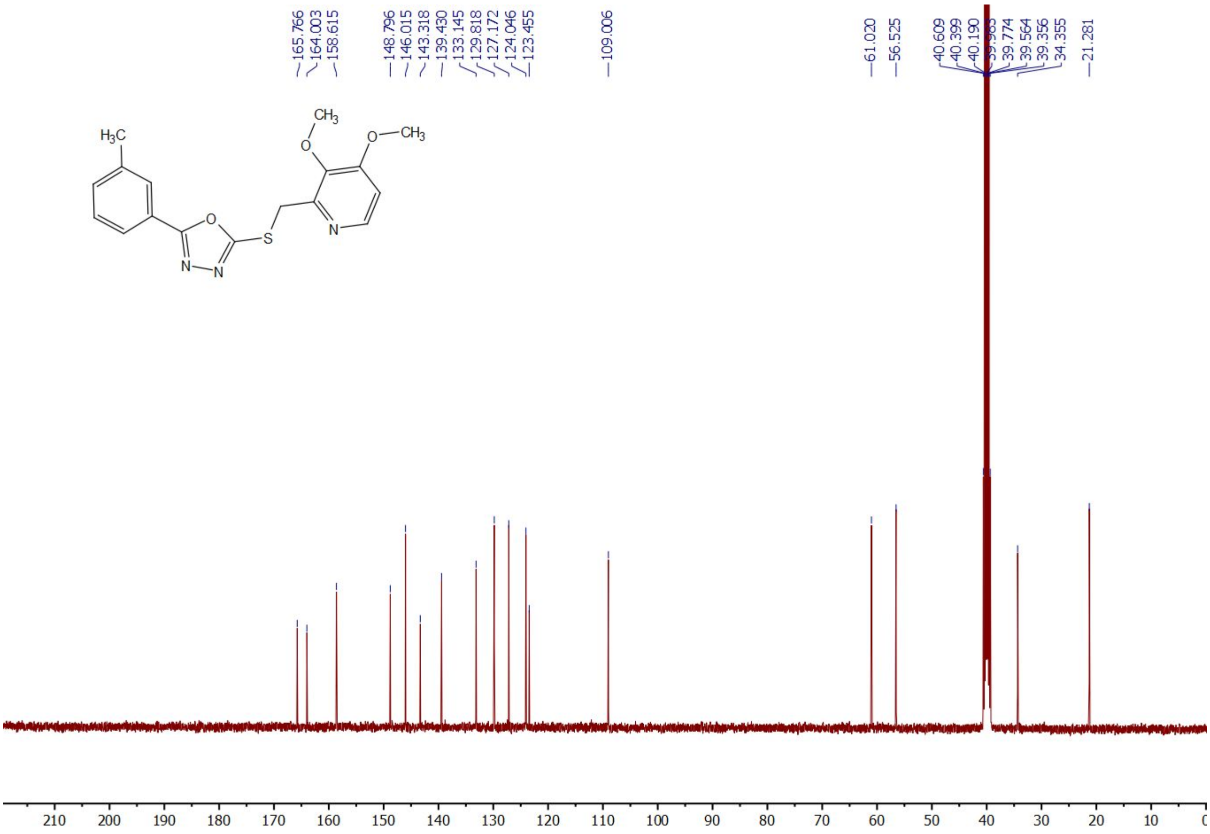

Figure S30: ^{13}C-NMR (100 MHz, DMSO-d$_6$) spectrum of compound **5f**

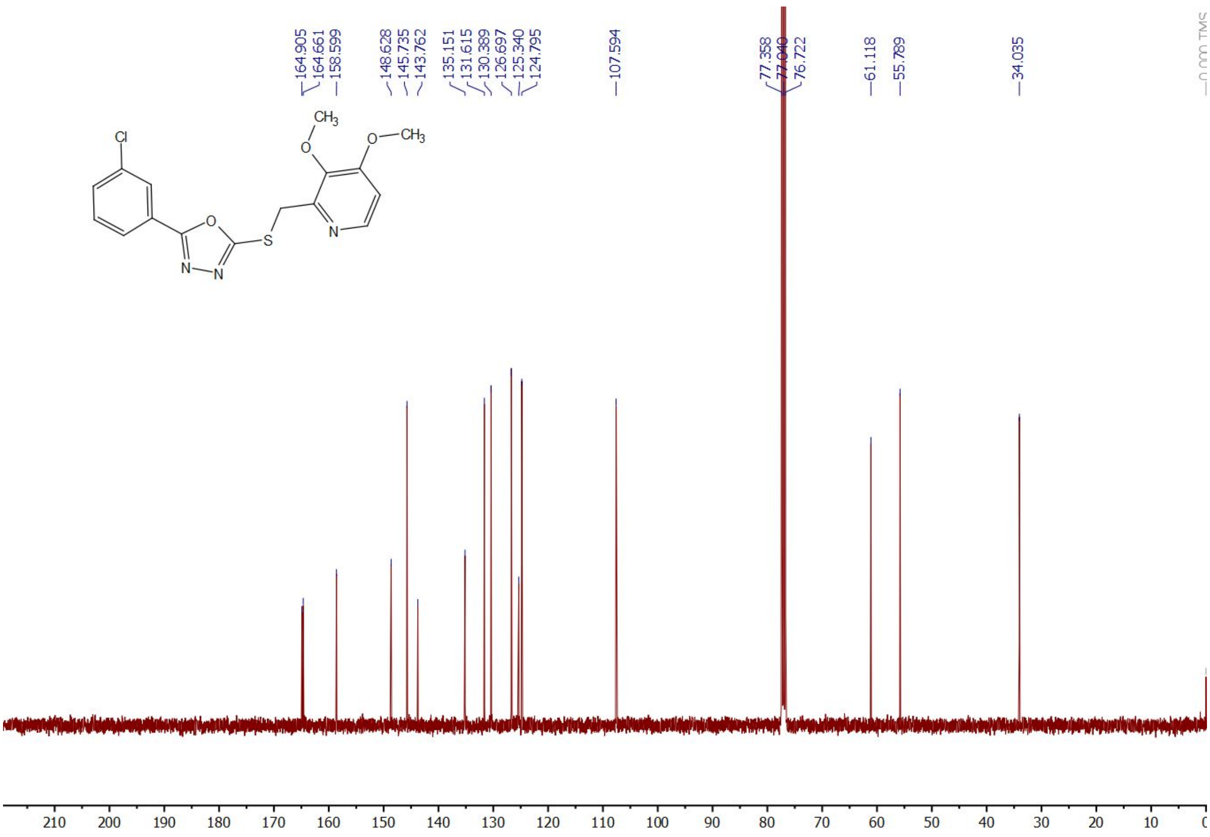

Figure S31: ^{13}C-NMR (100 MHz, $CDCl_3$) spectrum of compound **5g**

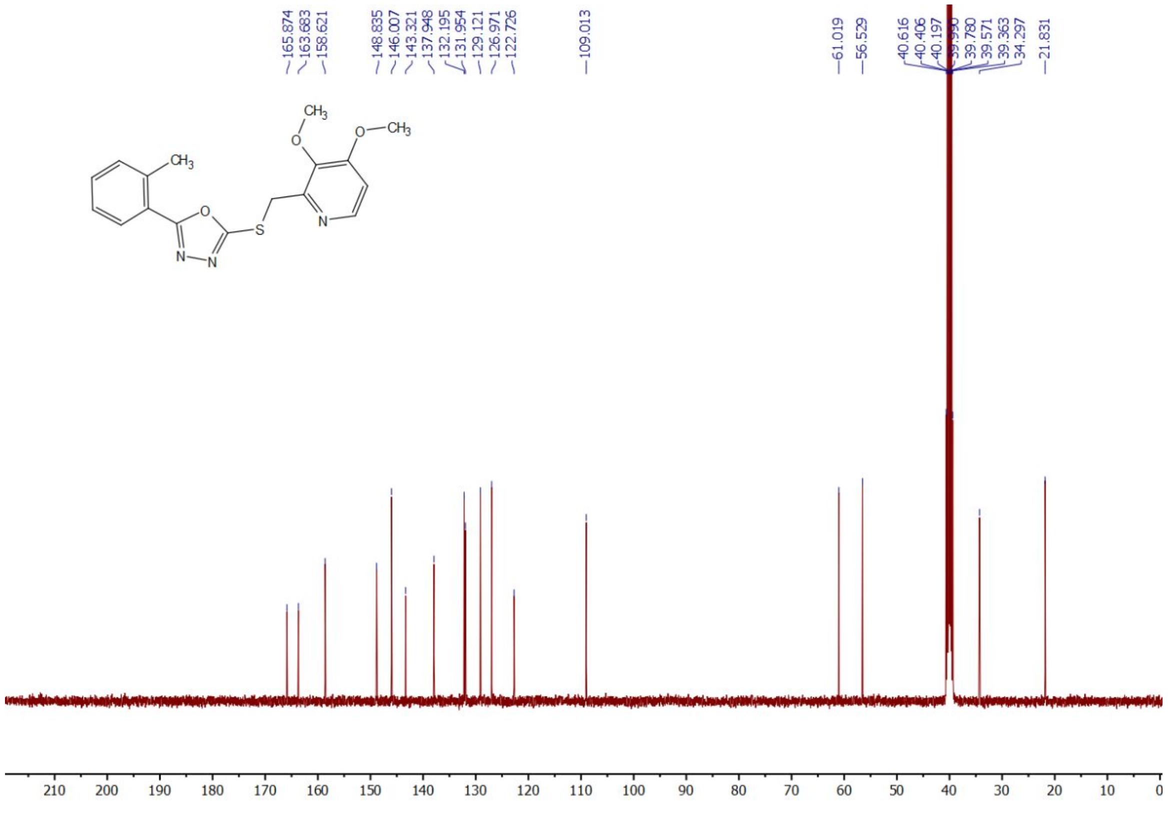

Figure S32: ^{13}C-NMR (100 MHz, DMSO-d6) spectrum of compound **5h**

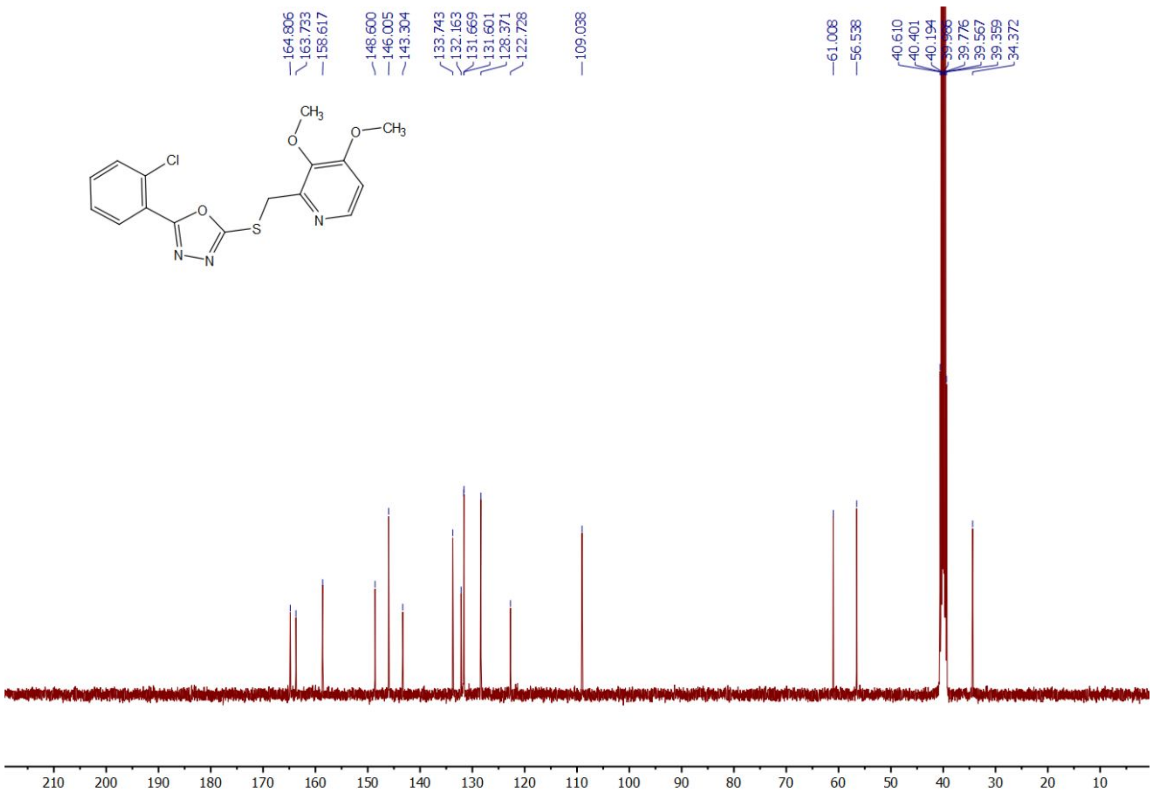

Figure S33: ^{13}C-NMR (100 MHz, DMSO-d6) spectrum of compound **5i**

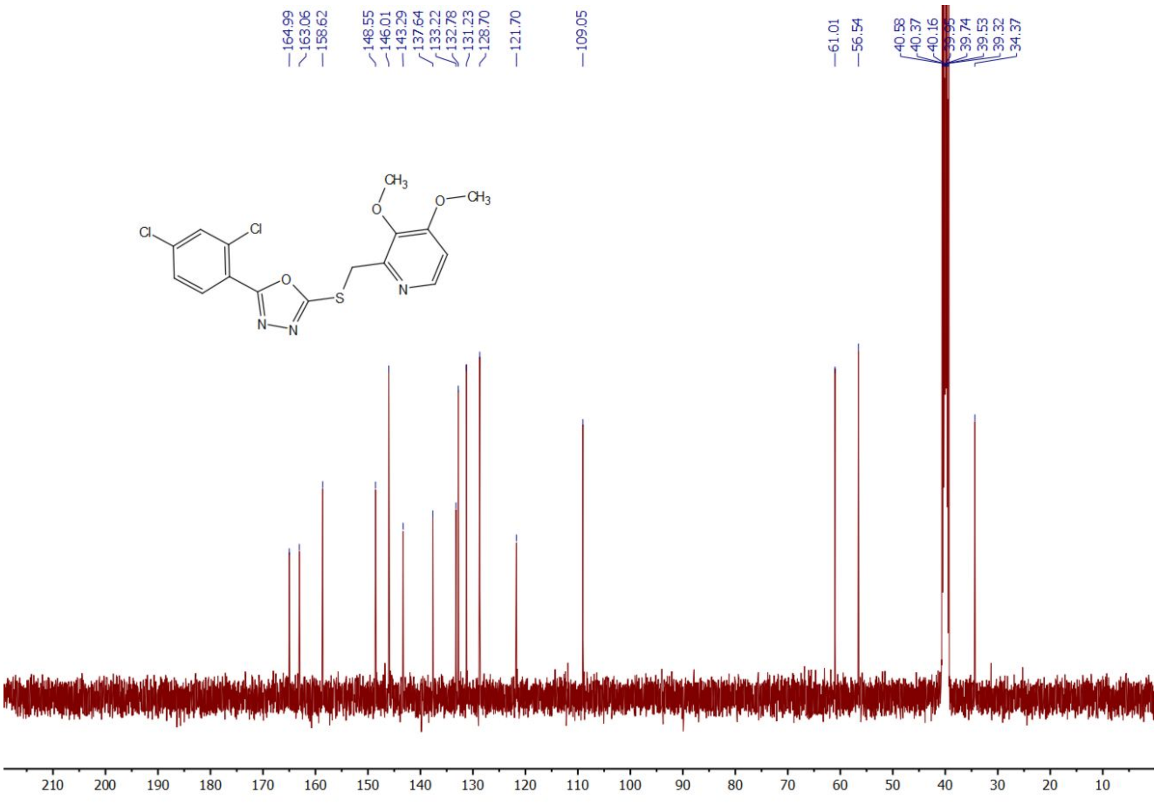

Figure S34: ^{13}C-NMR (100 MHz, DMSO-d$_6$) spectrum of compound **5j**

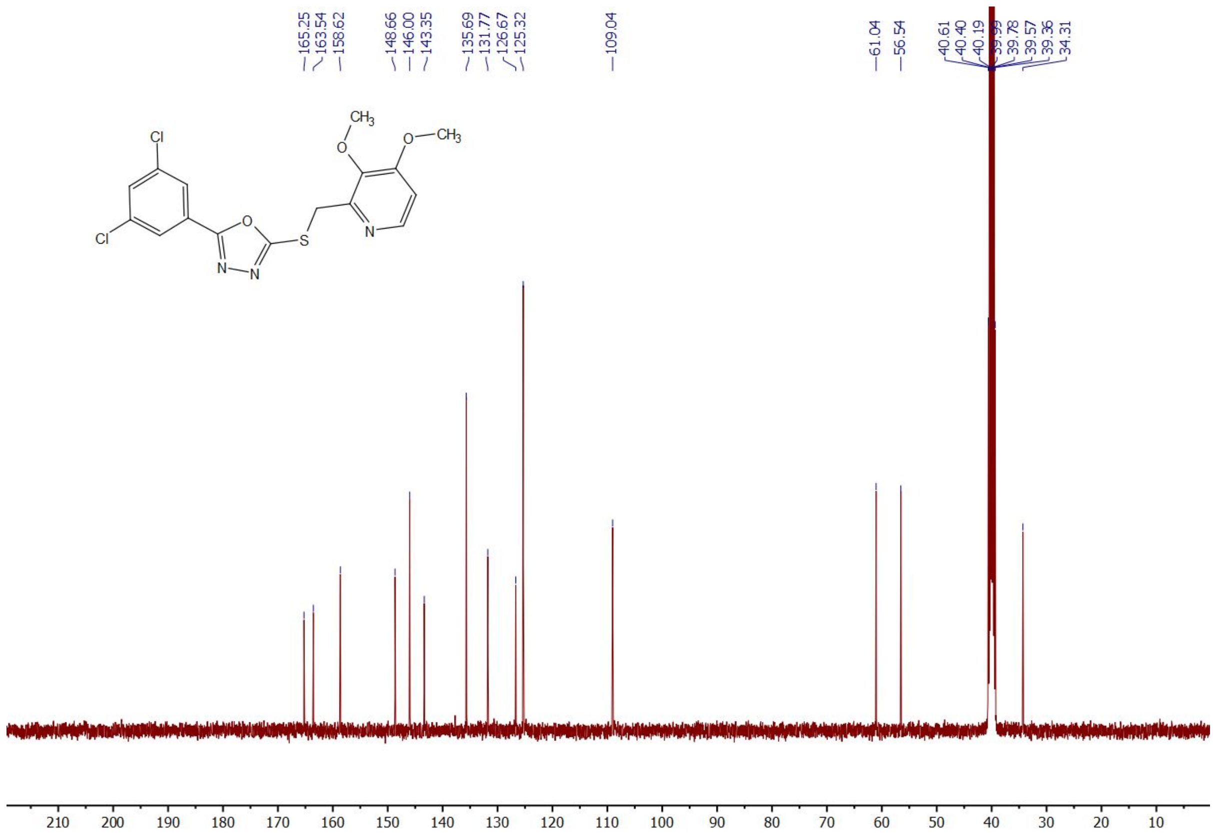

Figure S35: ^{13}C-NMR (100 MHz, DMSO-d$_6$) spectrum of compound **5k**

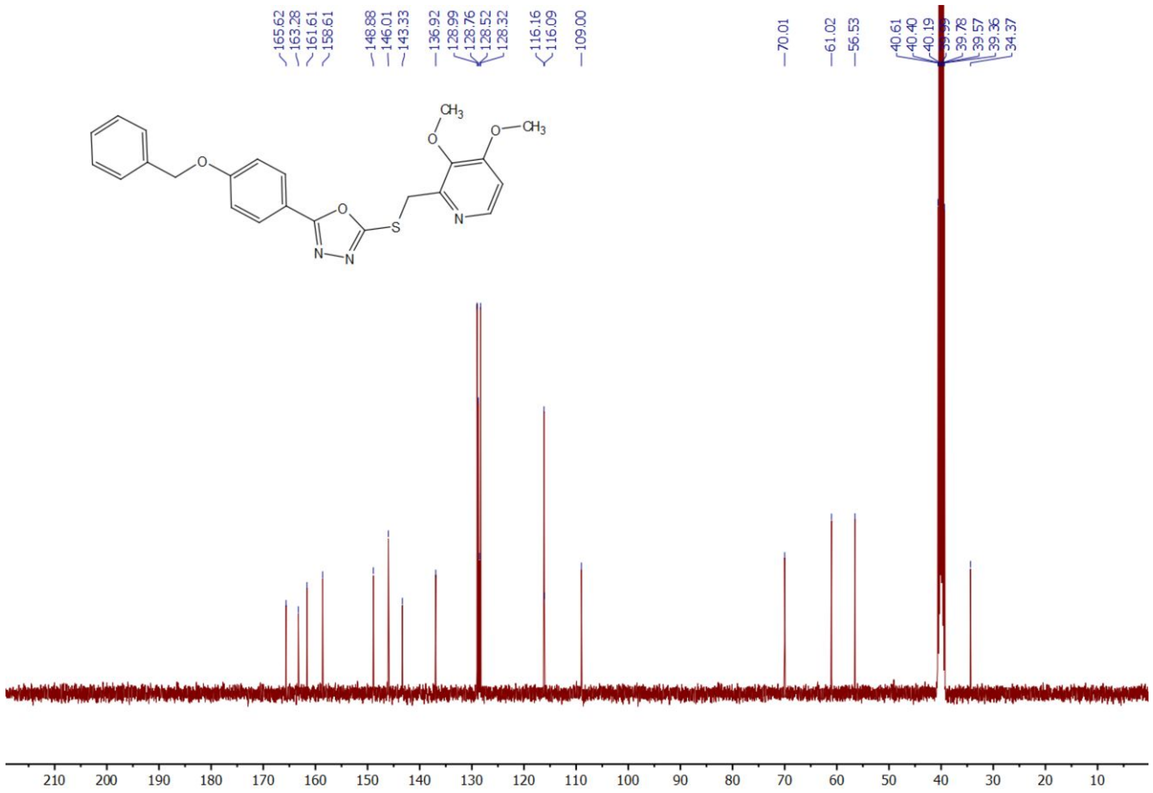

Figure S36: ^{13}C-NMR (100 MHz, DMSO-d$_6$) spectrum of compound **5l**

HRMS of Pyridine Based 1,3,4-Oxadizole Derivatives (5a–l)

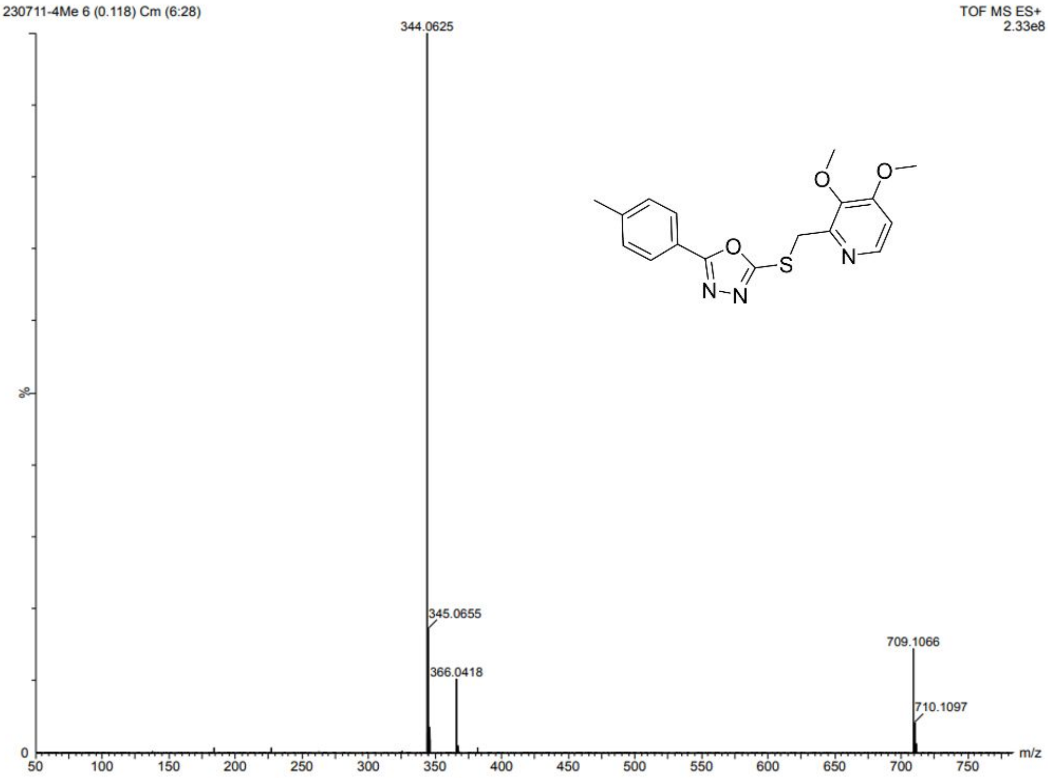

Figure S37: HRMS of compound **5a**

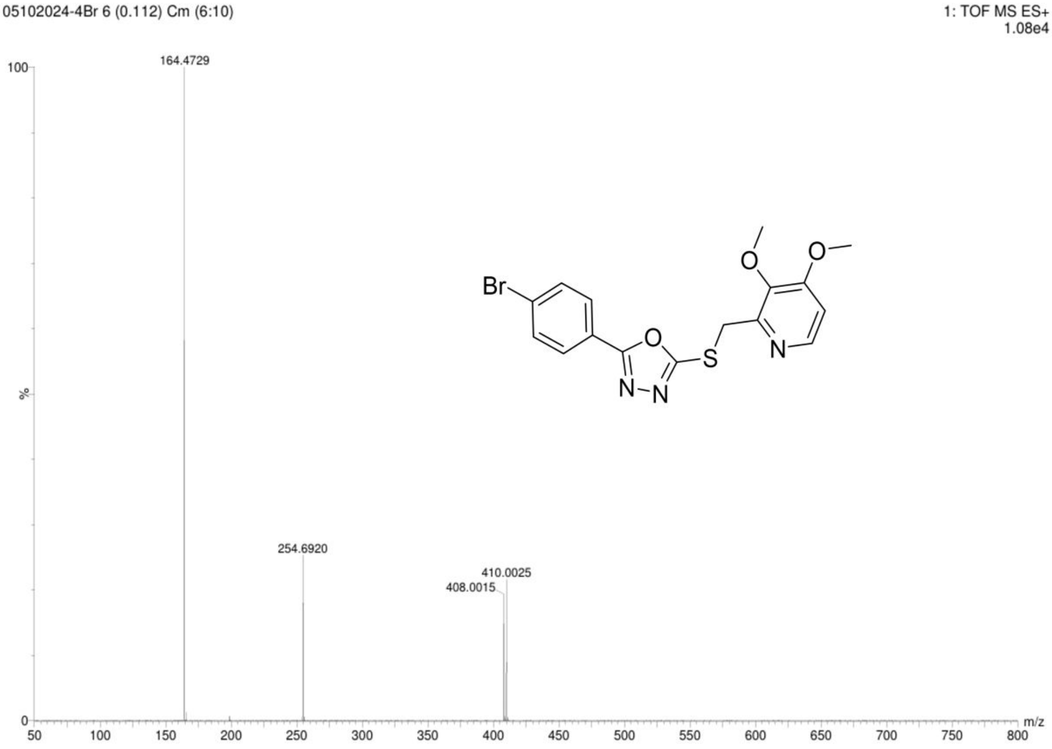

Figure S38: HRMS of compound **5b**

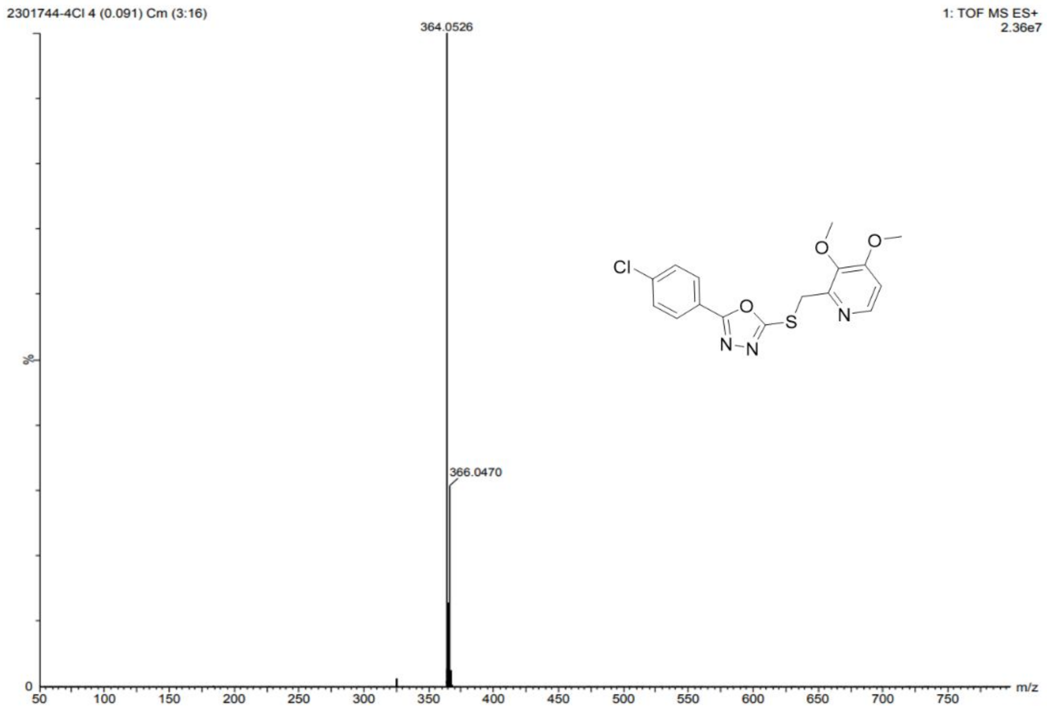

Figure S39: HRMS of compound **5c**

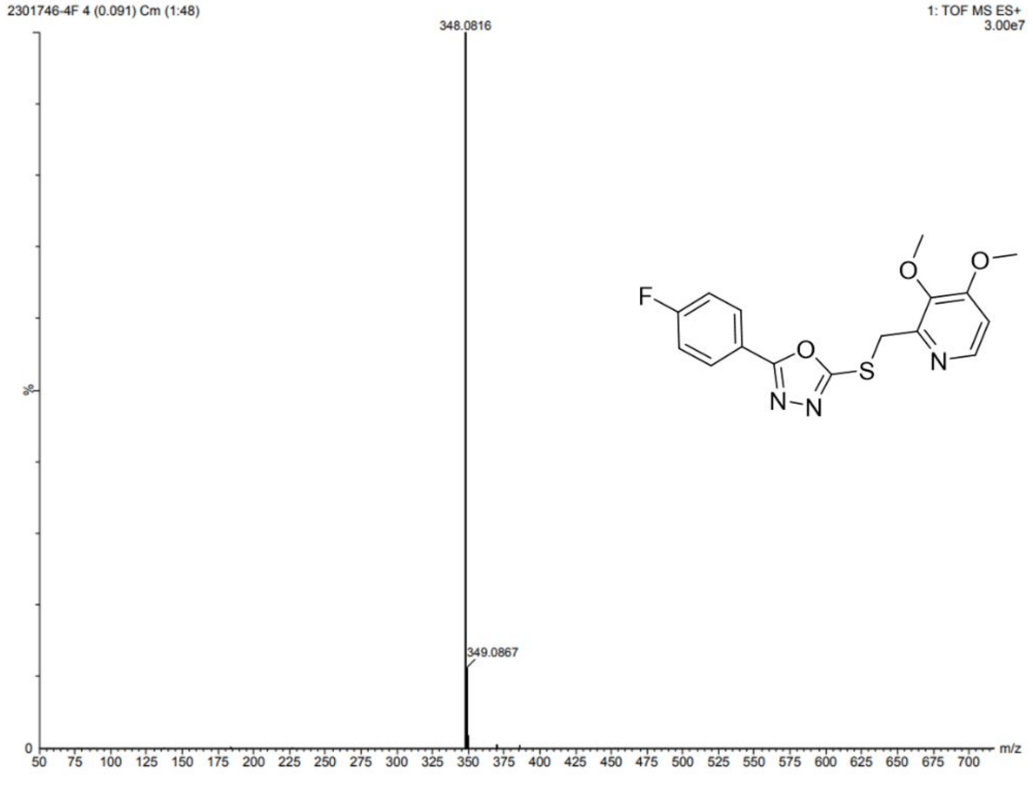

Figure S40: HRMS of compound **5d**

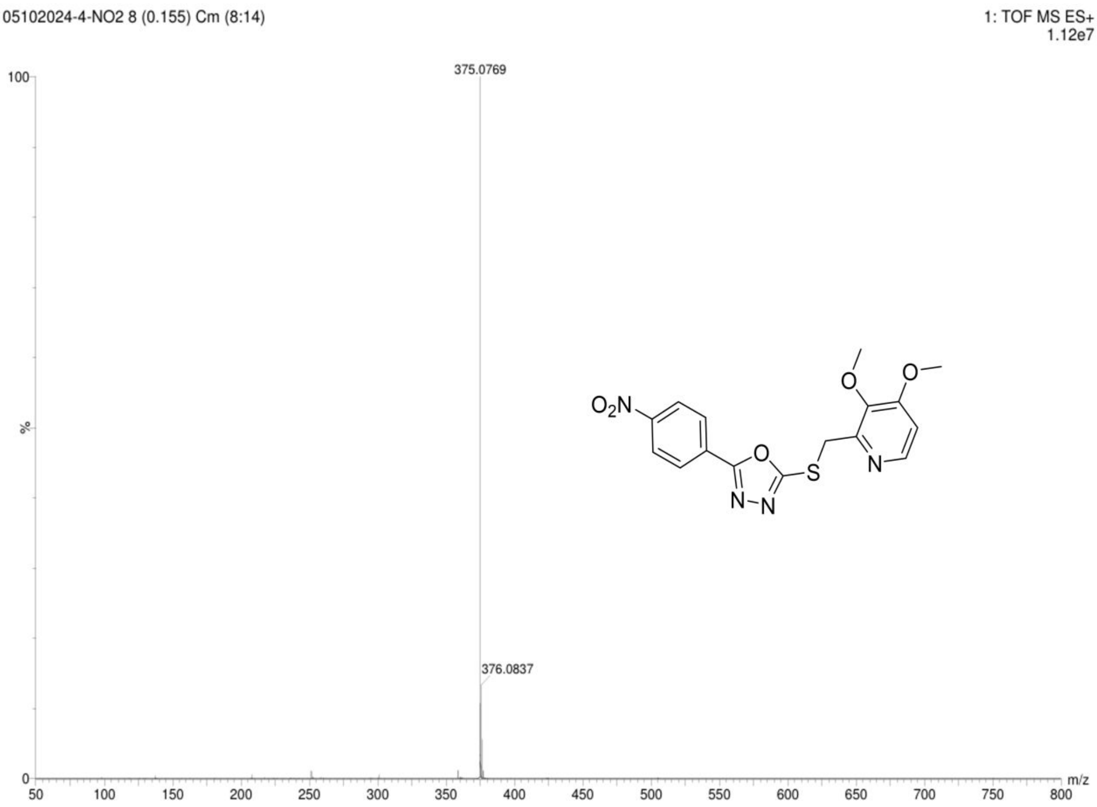

Figure S41: HRMS of compound **5e**

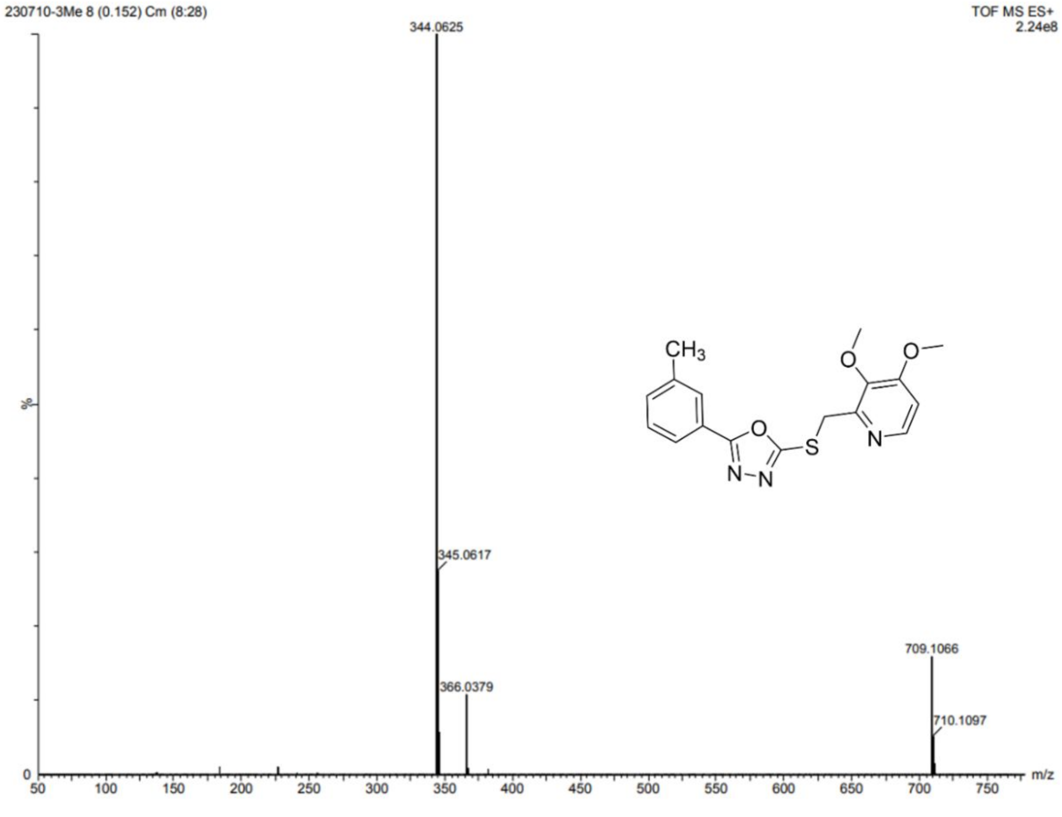

Figure S42: HRMS of compound **5f**

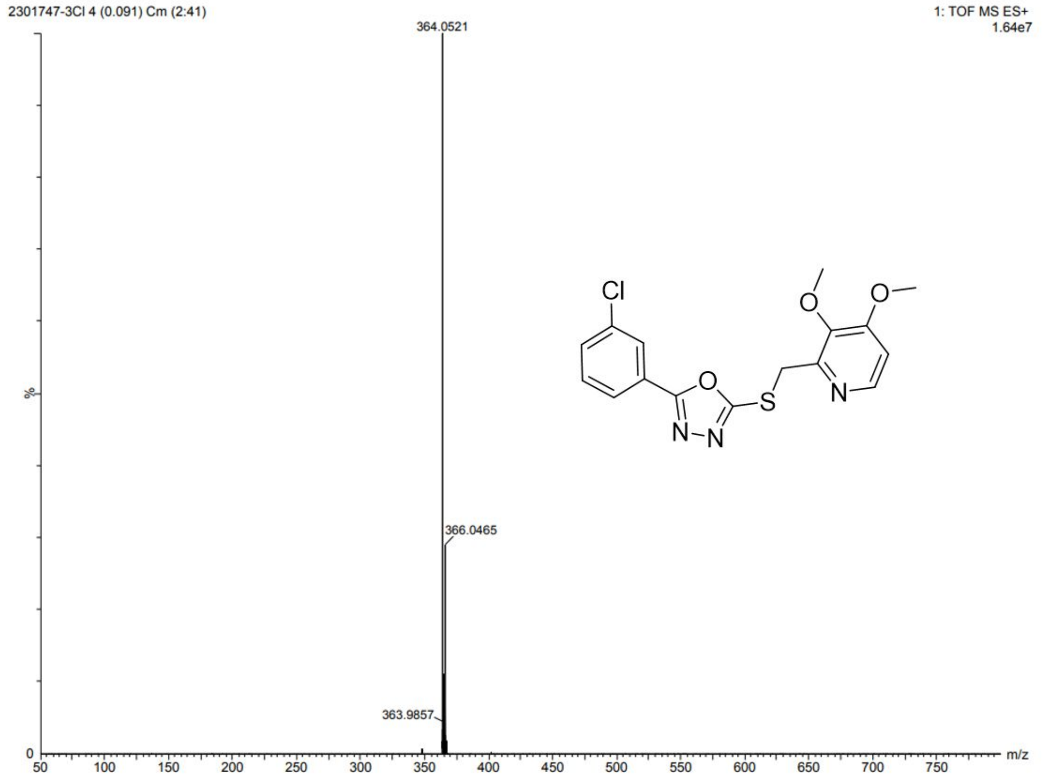

Figure S43: HRMS of compound **5g**

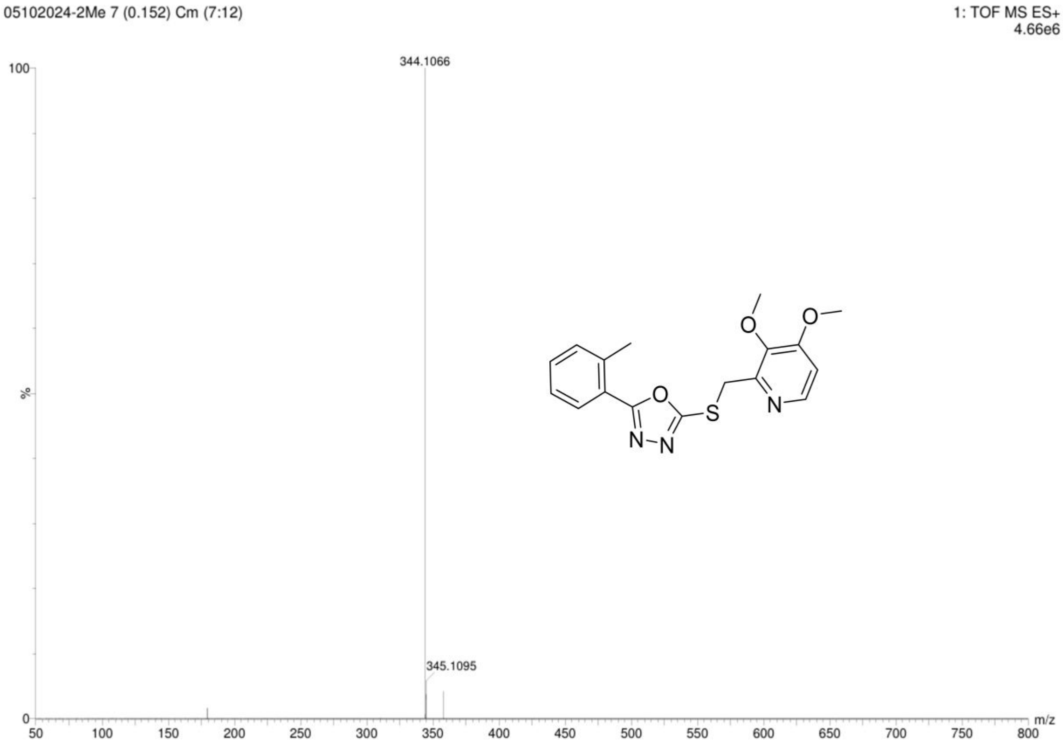

Figure S44: HRMS of compound **5h**

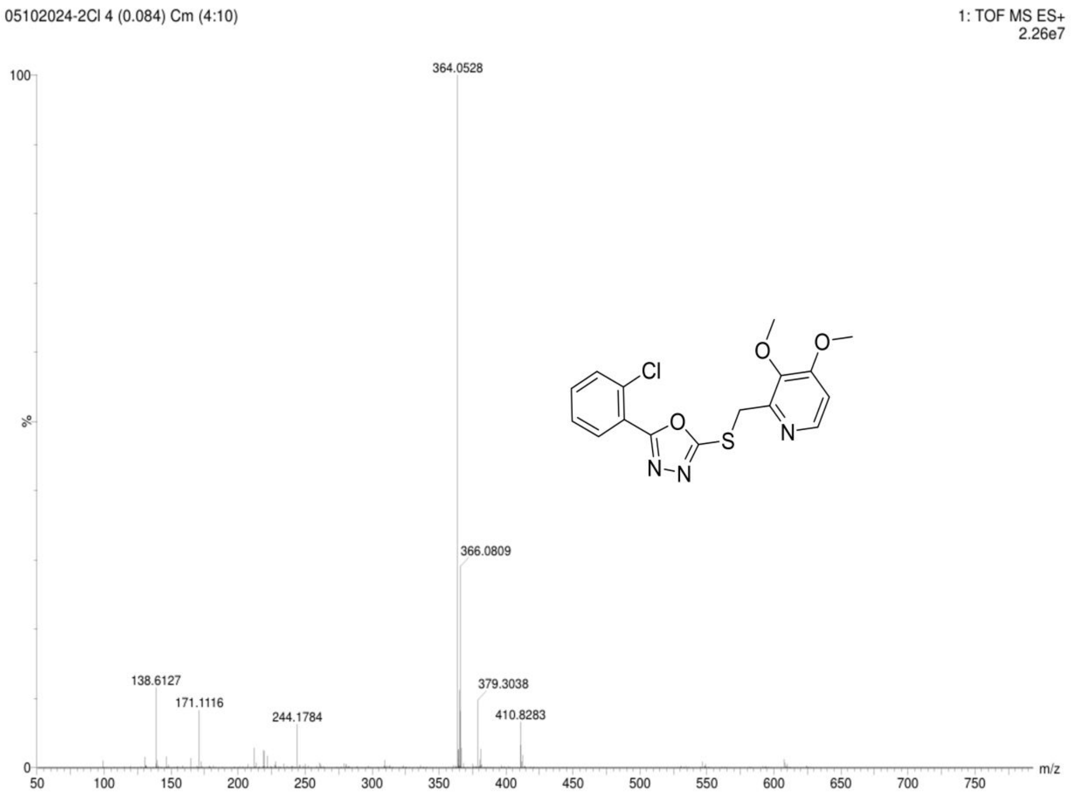

Figure S45: HRMS of compound **5i**

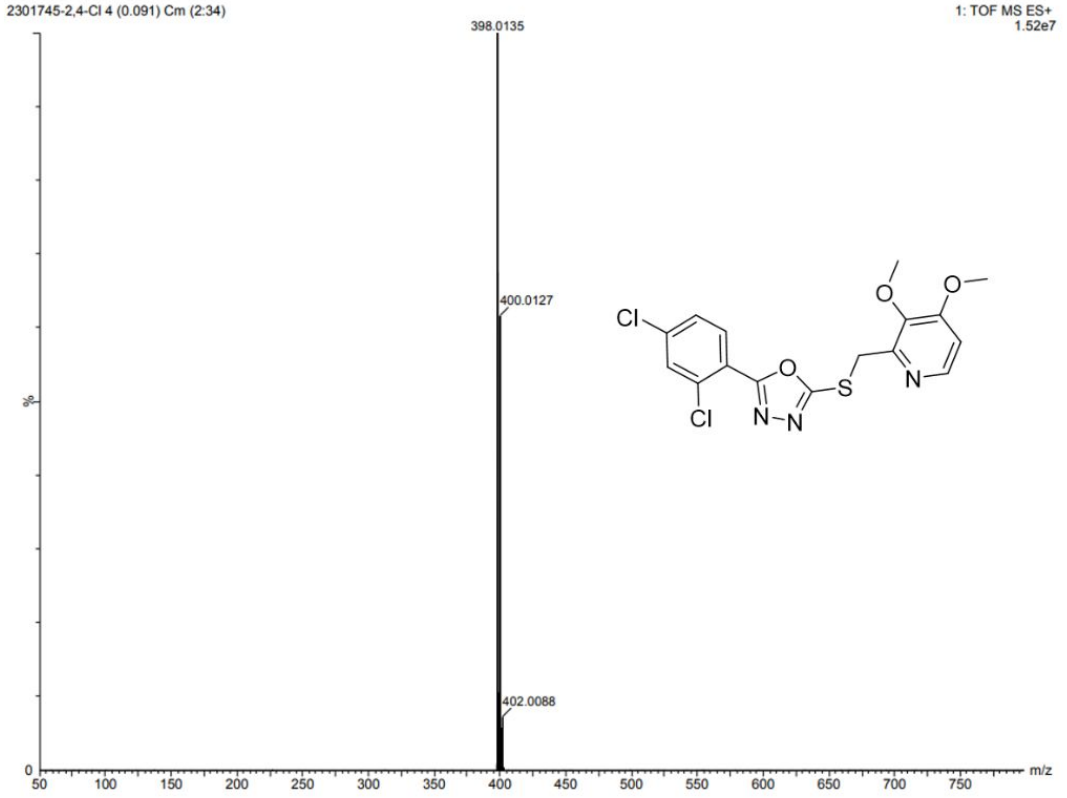

Figure S46: HRMS of compound **5j**

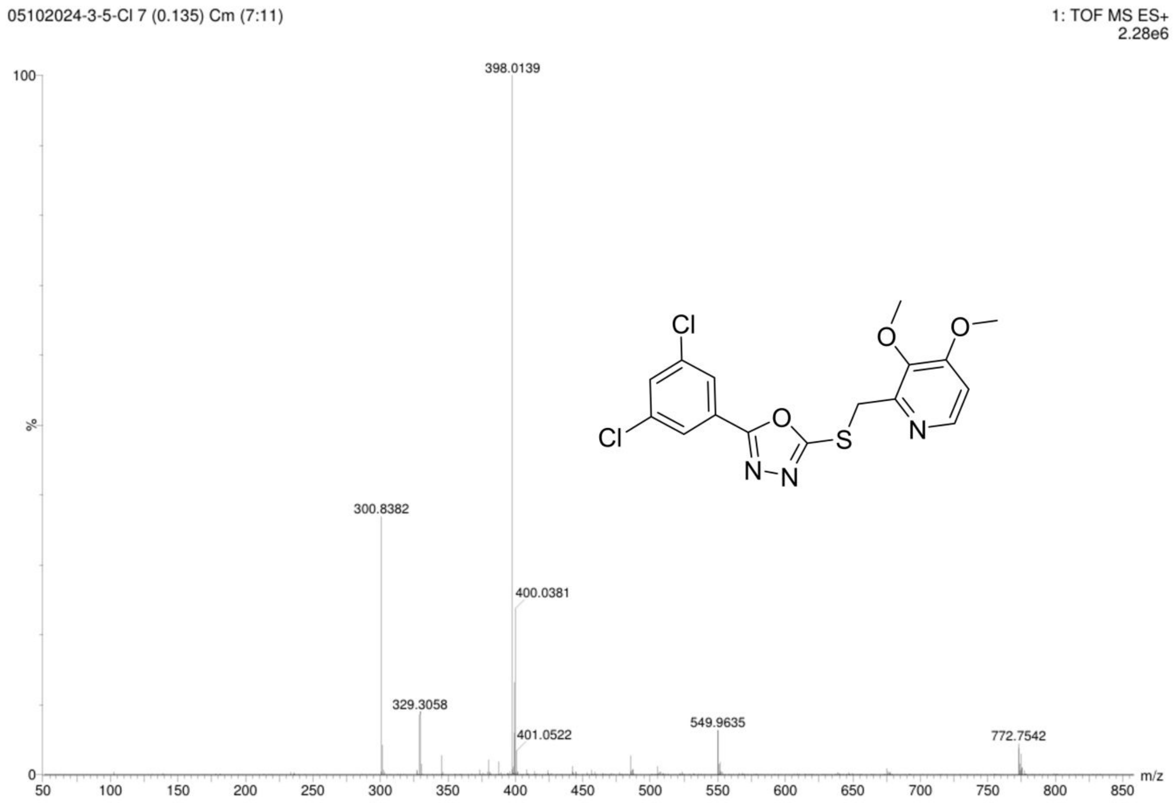

Figure S47: HRMS of compound **5k**

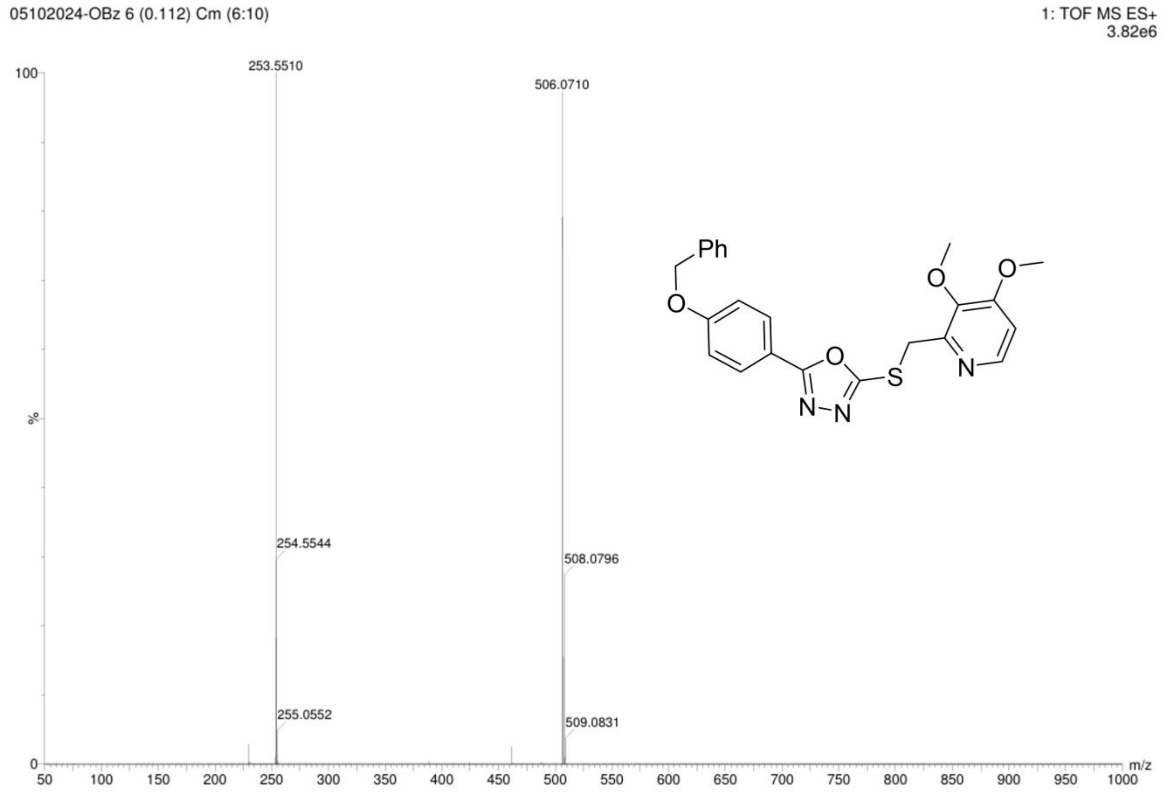

Figure S481: HRMS of compound **5l**

3] SC-XRD

Table S1: Data collection and structure refinement for **5f**

**Theta range for data collection**	2.71 to 28.42°	
**Index ranges**	-19<=h<=19, -11<=k<=12, -32<=l<=31	
**Reflections collected**	39963	
**Independent reflections**	4165 [R(int) = 0.0938]	
**Coverage of independent reflections**	99.2%	
**Absorption correction**	Multi-Scan	
**Structure solution technique**	direct methods	
**Structure solution program**	XT, VERSION 2018/2	
**Refinement method**	Full-matrix least-squares on F^2	
**Refinement program**	SHELXL-2019/1 (Sheldrick, 2019)	
**Function minimized**	$\Sigma w(F_o^2 - F_c^2)^2$	
**Data / restraints / parameters**	4165 / 0 / 221	
**Goodness-of-fit on F^2**	1.038	
**Δ/σ_{max}**	0.001	
**Final R indices**	2251 data; I>2σ(I)	R1 = 0.0487, wR2 = 0.0991
	all data	R1 = 0.1258, wR2 = 0.1318
**Weighting scheme**	$w=1/[\sigma^2(F_o^2)+(0.0441P)^2+1.2922P]$ where $P=(F_o^2+2F_c^2)/3$	
**Extinction coefficient**	0.0060(10)	
**Largest diff. peak and hole**	0.201 and -0.217 eÅ^{-3}	
**R.M.S. deviation from mean**	0.046 eÅ^{-3}	

Table S2: Atomic coordinates and equivalent isotropic atomic displacement parameters (Å2) for **5f**

U(eq) is defined as one third of the trace of the orthogonalized U_{ij} tensor.

	x/a	y/b	z/c	U(eq)
S1	0.54940(4)	0.44336(6)	0.57383(2)	0.0474(2)
O1	0.66701(11)	0.23384(18)	0.42037(7)	0.0585(5)
O2	0.62103(9)	0.63514(15)	0.64059(6)	0.0445(4)
O3	0.56354(12)	0.02556(18)	0.37125(7)	0.0656(5)
N1	0.72161(13)	0.5609(2)	0.57904(8)	0.0543(5)
N2	0.76478(13)	0.6604(2)	0.61411(8)	0.0565(6)
N3	0.46483(12)	0.2436(2)	0.50725(8)	0.0481(5)
C1	0.71501(15)	0.7994(2)	0.69462(9)	0.0457(6)
C2	0.80318(17)	0.8383(3)	0.70978(11)	0.0619(7)
C3	0.81523(19)	0.9307(3)	0.75301(12)	0.0765(9)
C4	0.7411(2)	0.9856(3)	0.78027(10)	0.0666(7)
C5	0.65297(17)	0.9497(3)	0.76603(10)	0.0559(7)
C6	0.64075(16)	0.8545(3)	0.72291(10)	0.0519(6)
C7	0.70398(14)	0.6998(2)	0.64915(9)	0.0450(6)
C8	0.63844(15)	0.5492(2)	0.59632(9)	0.0424(5)
C9	0.5729(2)	0.0131(4)	0.79623(13)	0.0905(10)
C10	0.60994(15)	0.3703(2)	0.51569(9)	0.0489(6)
C11	0.54932(14)	0.2649(2)	0.48678(9)	0.0426(5)
C12	0.41275(15)	0.1475(2)	0.48118(10)	0.0517(6)
C13	0.44041(16)	0.0701(2)	0.43616(10)	0.0511(6)
C14	0.52754(16)	0.0935(2)	0.41543(9)	0.0484(6)
C15	0.58237(14)	0.1956(2)	0.44079(9)	0.0434(5)
C16	0.5199(2)	0.8974(3)	0.35387(11)	0.0769(9)
C17	0.73846(18)	0.1330(4)	0.42957(12)	0.0780(9)

Table S3: Bond lengths (Å) for **5f**

S1-C8	1.724(2)	S1-C10	1.805(2)
O1-C15	1.379(3)	O1-C17	1.425(3)
O2-C7	1.369(2)	O2-C8	1.371(2)
O3-C14	1.357(3)	O3-C16	1.425(3)
N1-C8	1.289(3)	N1-N2	1.414(3)
N2-C7	1.286(3)	N3-C12	1.339(3)
N3-C11	1.344(3)	C1-C6	1.384(3)
C1-C2	1.387(3)	C1-C7	1.459(3)
C2-C3	1.376(3)	C2-H2	0.930000
C3-C4	1.369(4)	C3-H3	0.930000
C4-C5	1.373(4)	C4-H4	0.930000
C5-C6	1.391(3)	C5-C9	1.504(3)
C6-H6	0.930000	C9-H9A	0.960000
C9-H9B	0.960000	C9-H9C	0.960000
C10-C11	1.502(3)	C10-H10A	0.970000
C10-H10B	0.970000	C11-C15	1.383(3)
C12-C13	1.376(3)	C12-H12	0.930000
C13-C14	1.385(3)	C13-H13	0.930000
C14-C15	1.392(3)	C16-H16A	0.960000
C16-H16B	0.960000	C16-H16C	0.960000
C17-H17A	0.960000	C17-H17B	0.960000
C17-H17C	0.960000		

Table S4: Torsion angles (°) for **5f**

C8-N1-N2-C7	-0.1(3)	C6-C1-C2-C3	0.3(4)
C7-C1-C2-C3	-179.2(2)	C1-C2-C3-C4	-1.0(4)
C2-C3-C4-C5	0.7(5)	C3-C4-C5-C6	0.4(4)
C3-C4-C5-C9	-179.2(3)	C2-C1-C6-C5	0.7(4)
C7-C1-C6-C5	-179.8(2)	C4-C5-C6-C1	-1.0(4)
C9-C5-C6-C1	178.5(3)	N1-N2-C7-O2	-0.4(3)
N1-N2-C7-C1	178.8(2)	C8-O2-C7-N2	0.8(2)
C8-O2-C7-C1	-178.50(19)	C6-C1-C7-N2	167.1(2)
C2-C1-C7-N2	-13.4(4)	C6-C1-C7-O2	-13.7(3)
C2-C1-C7-O2	165.8(2)	N2-N1-C8-O2	0.7(3)
N2-N1-C8-S1	-178.03(18)	C7-O2-C8-N1	-0.9(2)
C7-O2-C8-S1	178.01(15)	C10-S1-C8-N1	-4.8(2)
C10-S1-C8-O2	176.55(16)	C8-S1-C10-C11	178.16(16)
C12-N3-C11-C15	1.3(3)	C12-N3-C11-C10	-179.02(19)
S1-C10-C11-N3	0.4(3)	S1-C10-C11-C15	-179.92(16)
C11-N3-C12-C13	0.7(3)	N3-C12-C13-C14	-0.9(4)
C16-O3-C14-C13	-17.3(3)	C16-O3-C14-C15	163.9(2)
C12-C13-C14-O3	-179.7(2)	C12-C13-C14-C15	-0.8(3)
C17-O1-C15-C11	107.2(2)	C17-O1-C15-C14	-75.4(3)
N3-C11-C15-O1	174.50(19)	C10-C11-C15-O1	-5.2(3)
N3-C11-C15-C14	-3.0(3)	C10-C11-C15-C14	177.3(2)
O3-C14-C15-O1	4.2(3)	C13-C14-C15-O1	-174.7(2)
O3-C14-C15-C11	-178.4(2)	C13-C14-C15-C11	2.7(3)

Table S5: Hydrogen bond distances (Å) and angles (°) for **5f**

	Donor-H	Acceptor-H	Donor-Acceptor	Angle
C17-H17B···O3	0.96	2.54	3.090(3)	116.7

Table S6: Bond angles (°) for **5f**

C8-S1-C10	95.74(10)	C15-O1-C17	115.16(19)
C7-O2-C8	102.52(16)	C14-O3-C16	117.34(19)
C8-N1-N2	106.11(18)	C7-N2-N1	106.54(18)
C12-N3-C11	116.3(2)	C6-C1-C2	119.6(2)
C6-C1-C7	122.1(2)	C2-C1-C7	118.2(2)
C3-C2-C1	119.2(2)	C3-C2-H2	120.400000
C1-C2-H2	120.400000	C4-C3-C2	120.5(3)
C4-C3-H3	119.700000	C2-C3-H3	119.700000
C3-C4-C5	121.6(2)	C3-C4-H4	119.200000
C5-C4-H4	119.200000	C4-C5-C6	117.9(2)
C4-C5-C9	120.4(2)	C6-C5-C9	121.6(2)
C1-C6-C5	121.1(2)	C1-C6-H6	119.500000
C5-C6-H6	119.500000	N2-C7-O2	112.38(19)
N2-C7-C1	127.8(2)	O2-C7-C1	119.79(19)
N1-C8-O2	112.45(19)	N1-C8-S1	130.82(18)
O2-C8-S1	116.71(15)	C5-C9-H9A	109.500000
C5-C9-H9B	109.500000	H9A-C9-H9B	109.500000
C5-C9-H9C	109.500000	H9A-C9-H9C	109.500000
H9B-C9-H9C	109.500000	C11-C10-S1	109.31(15)
C11-C10-H10A	109.800000	S1-C10-H10A	109.800000
C11-C10-H10B	109.800000	S1-C10-H10B	109.800000
H10A-C10-H10B	108.300000	N3-C11-C15	123.4(2)
N3-C11-C10	117.6(2)	C15-C11-C10	119.01(19)
N3-C12-C13	124.5(2)	N3-C12-H12	117.700000
C13-C12-H12	117.700000	C12-C13-C14	118.5(2)
C12-C13-H13	120.800000	C14-C13-H13	120.800000
O3-C14-C13	124.8(2)	O3-C14-C15	116.9(2)
C13-C14-C15	118.3(2)	O1-C15-C11	118.86(19)
O1-C15-C14	122.2(2)	C11-C15-C14	118.9(2)
O3-C16-H16A	109.500000	O3-C16-H16B	109.500000
H16A-C16-H16B	109.500000	O3-C16-H16C	109.500000
H16A-C16-H16C	109.500000	H16B-C16-H16C	109.500000
O1-C17-H17A	109.500000	O1-C17-H17B	109.500000
H17A-C17-H17B	109.500000	O1-C17-H17C	109.500000
H17A-C17-H17C	109.500000	H17B-C17-H17C	109.500000

Table S7: Anisotropic atomic displacement parameters (Å2) for **5f**

The anisotropic atomic displacement factor exponent takes the form: $-2\pi^2[h^2 a^{*2} U_{11} + ... + 2 h k a^* b^* U_{12}]$

	U_{11}	U_{22}	U_{33}	U_{23}	U_{13}	U_{12}
S1	0.0421(3)	0.0468(4)	0.0533(4)	-0.0024(3)	0.0027(3)	-0.0046(3)
O1	0.0492(9)	0.0612(11)	0.0650(11)	-0.0014(9)	0.0104(8)	-0.0126(9)
O2	0.0384(8)	0.0470(9)	0.0479(9)	-0.0019(8)	0.0038(7)	-0.0040(7)
O3	0.0804(12)	0.0644(11)	0.0520(11)	-0.0132(9)	0.0085(9)	-0.0240(10)
N1	0.0448(11)	0.0586(13)	0.0595(13)	-0.0143(10)	0.0050(10)	-0.0060(10)
N2	0.0441(11)	0.0631(13)	0.0621(13)	-0.0144(11)	0.0054(10)	-0.0088(10)
N3	0.0412(10)	0.0493(11)	0.0539(12)	0.0046(10)	-0.0012(9)	-0.0052(9)
C1	0.0463(13)	0.0463(13)	0.0445(13)	0.0029(11)	0.0016(11)	-0.0061(11)
C2	0.0462(14)	0.0746(18)	0.0649(17)	-0.0106(15)	0.0051(12)	-0.0056(13)
C3	0.0558(17)	0.101(2)	0.073(2)	-0.0269(18)	-0.0060(15)	-0.0182(16)
C4	0.0703(18)	0.0788(19)	0.0507(16)	-0.0146(14)	0.0039(14)	-0.0127(15)
C5	0.0566(15)	0.0660(17)	0.0449(14)	-0.0022(13)	0.0060(11)	-0.0039(13)
C6	0.0459(13)	0.0597(15)	0.0502(15)	-0.0027(12)	0.0022(11)	-0.0061(12)
C7	0.0399(12)	0.0455(13)	0.0496(14)	-0.0002(11)	0.0007(11)	-0.0046(10)
C8	0.0426(12)	0.0419(12)	0.0428(13)	-0.0008(11)	0.0023(10)	0.0016(10)
C9	0.079(2)	0.119(3)	0.074(2)	-0.0350(19)	0.0230(17)	0.0008(19)
C10	0.0416(12)	0.0556(14)	0.0496(14)	-0.0067(12)	0.0023(10)	-0.0041(11)
C11	0.0393(12)	0.0404(12)	0.0482(14)	0.0057(11)	-0.0037(10)	-0.0027(10)
C12	0.0398(12)	0.0538(14)	0.0616(16)	0.0091(13)	-0.0021(11)	-0.0073(11)
C13	0.0518(14)	0.0485(14)	0.0530(15)	0.0067(12)	-0.0112(11)	-0.0132(11)
C14	0.0554(14)	0.0457(13)	0.0442(14)	0.0042(11)	-0.0040(11)	-0.0061(11)
C15	0.0429(12)	0.0417(12)	0.0458(13)	0.0049(11)	-0.0019(10)	-0.0070(10)
C16	0.114(2)	0.0585(17)	0.0587(18)	-0.0099(14)	0.0045(17)	-0.0298(17)
C17	0.0487(15)	0.094(2)	0.091(2)	-0.0176(18)	0.0041(15)	0.0020(16)

Table S8: Hydrogen atomic coordinates and isotropic atomic displacement parameters (Å2) for **5f**

	x/a	y/b	z/c	U(eq)
H2	0.8536	0.8023	0.6910	0.074000
H3	0.8742	0.9561	0.7638	0.092000
H4	0.7507	1.0486	0.8091	0.080000
H6	0.5817	0.8275	0.7129	0.062000
H9A	0.5329	0.9382	0.8084	0.136000
H9B	0.5398	1.0759	0.7722	0.136000
H9C	0.5947	1.0658	0.8274	0.136000
H10A	0.6269	0.4464	0.4907	0.059000
H10B	0.6656	0.3235	0.5280	0.059000
H12	0.3538	0.1320	0.4944	0.062000
H13	0.4015	0.0037	0.4200	0.061000
H16A	0.5548	-0.1451	0.3248	0.115000
H16B	0.4592	-0.0813	0.3410	0.115000
H16C	0.5163	-0.1679	0.3841	0.115000
H17A	0.7947	0.1687	0.4145	0.117000
H17B	0.7230	0.0444	0.4122	0.117000
H17C	0.7458	0.1180	0.4683	0.117000

4] Hirshfield Surface Analysis

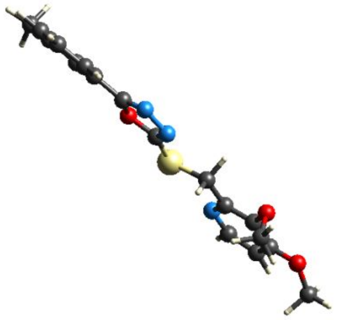	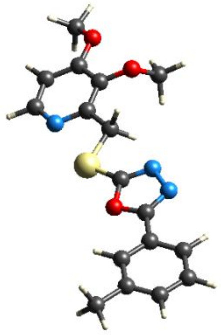
Along a axis	Along b axis
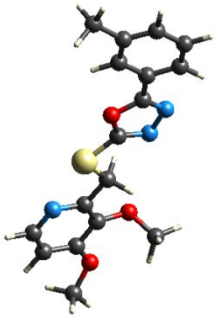	
Along c axis	

Figure S49: Molecular structure of **5f** along three different axes

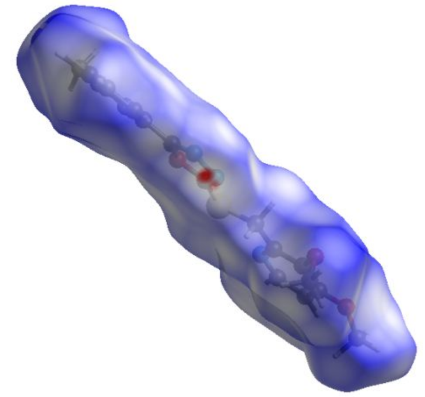	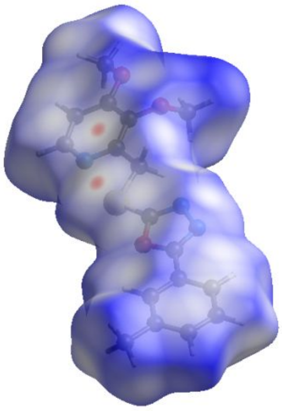
along a axis	Along b axis
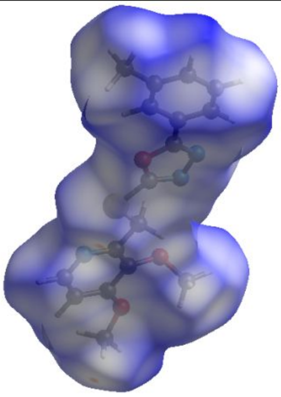	
Along c axis	

Figure S50: Hirshfeld surface (d_{norm} mapped in the range -0.1021 to 1.2464 au)

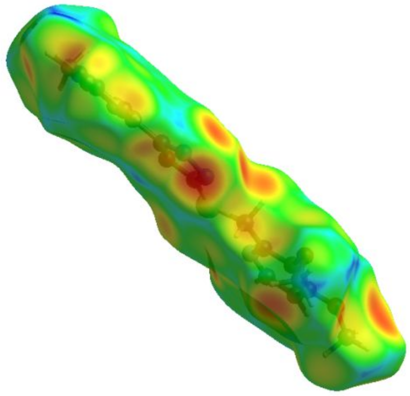	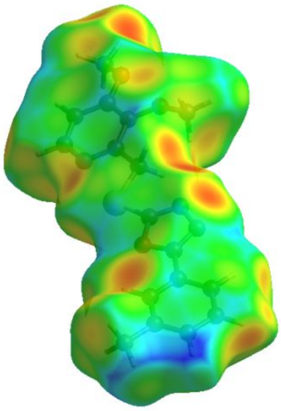
Along a axis	Along b axis

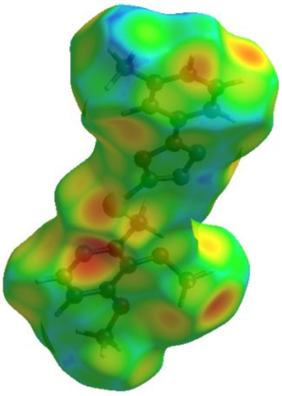	
Along c axis	

Figure S51: Hirshfeld surface (d_e mapped in the range 1.0371 to 2.5413 au)

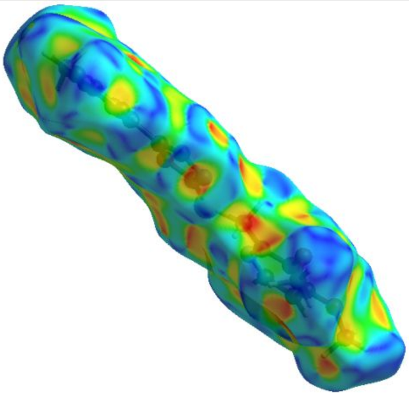	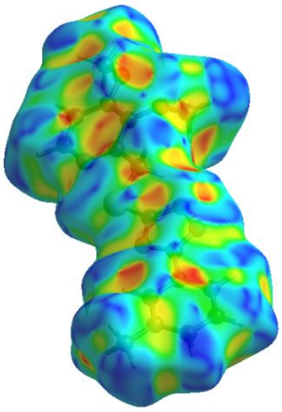
Along a axis	Along b axis
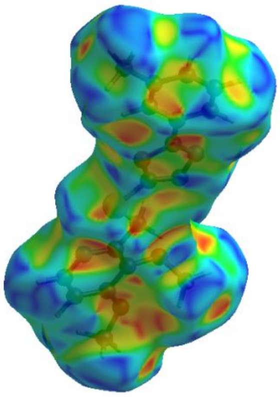	
Along c axis	

Figure S52: Shape index (range -1.0 to 1.0 au)

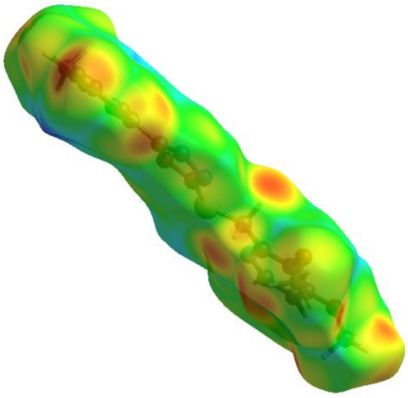	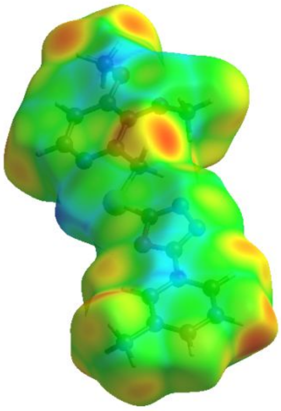
Along a axis	Along b axis
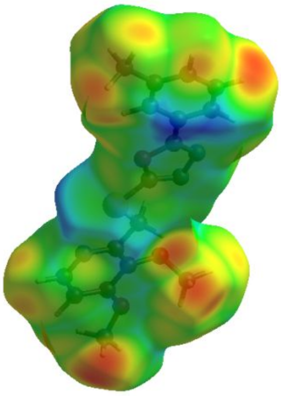	
Along c axis	

Figure S53: Hirshfeld surface (d_i mapped in the range 1.0369 to 2.5831 au)

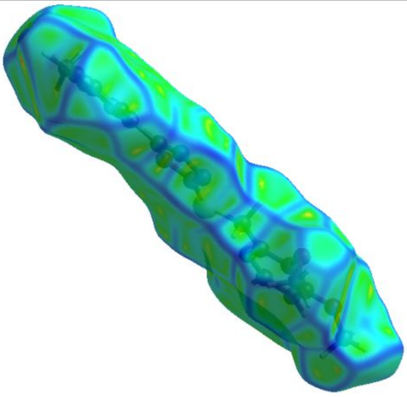	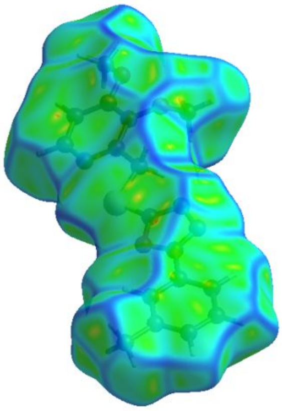
Along a axis	Along b axis

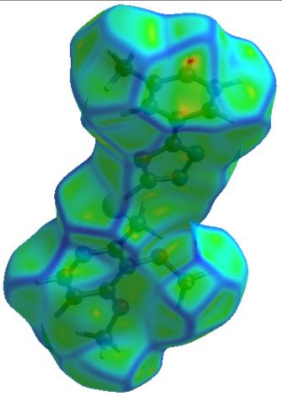	
Along c axis	

Figure S54: Hirshfeld surface (curvedness mapped in the range -4.00 to 4.0 au)

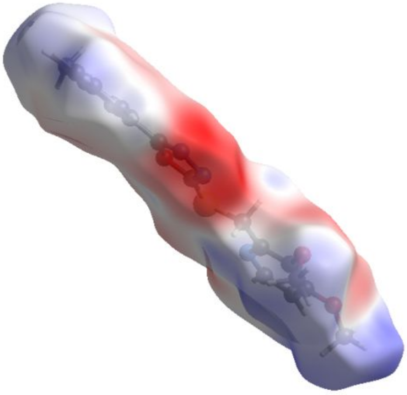	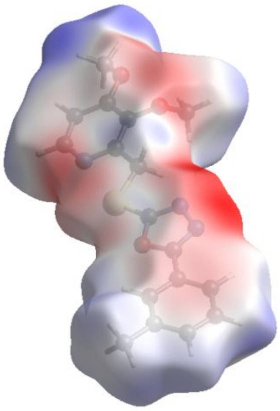
Along a axis	Along b axis
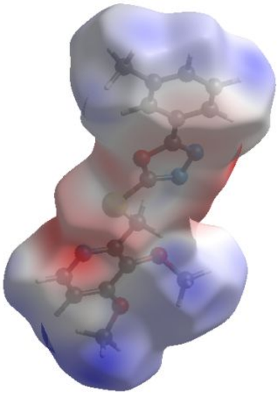	
Along c axis	

Figure S55: Electrostatic potential (calculated using B3LYP/6-31G(d,p); (range: -0.080 to 0.0632)

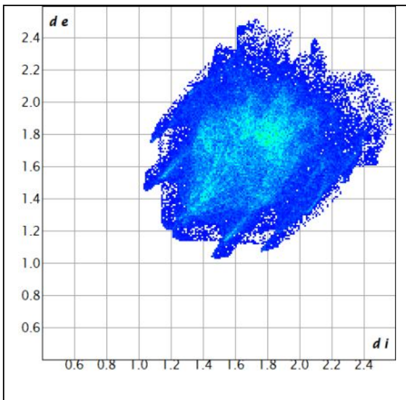

All-All

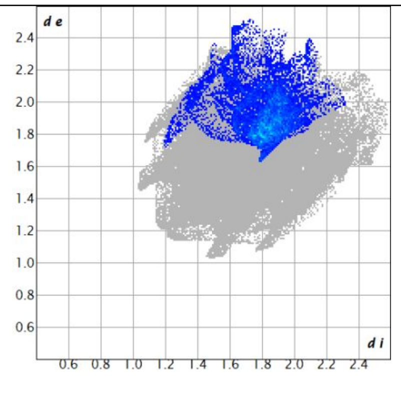

All-C

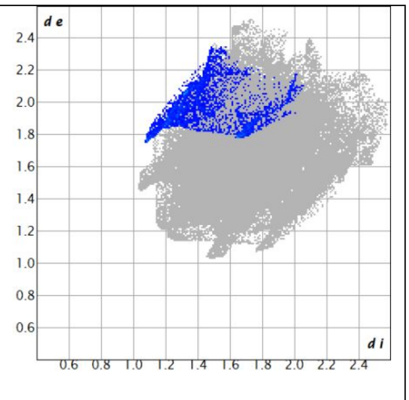

All-S

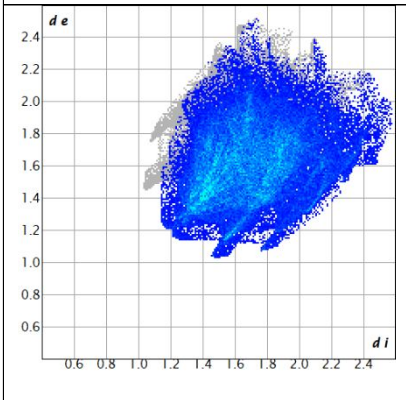

All-H

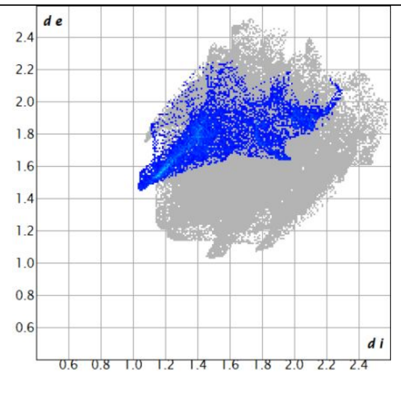

All-N

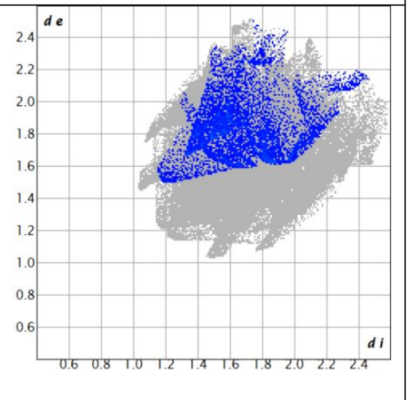

All – O

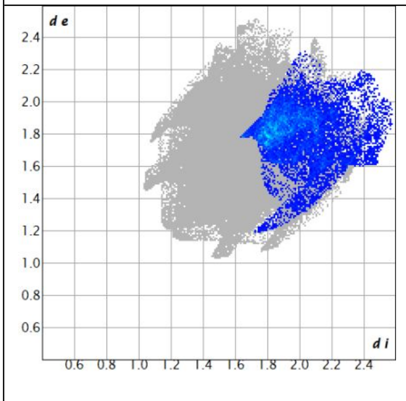

C - All

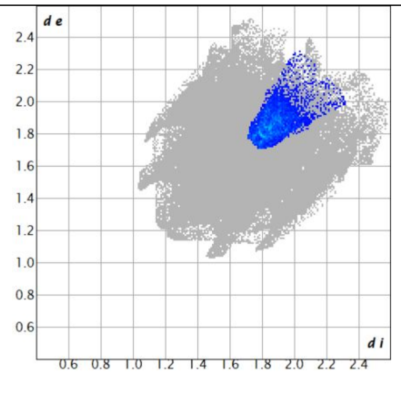

C - C

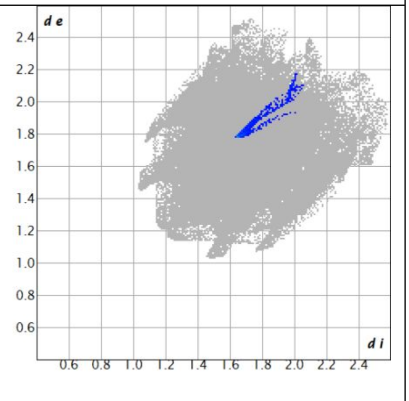

C – S

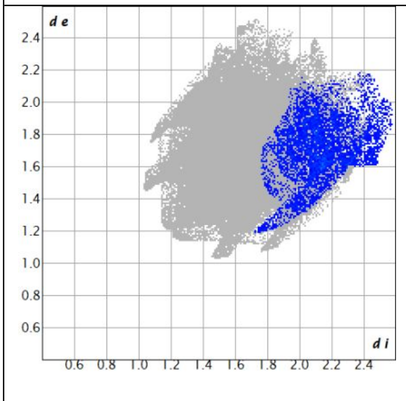

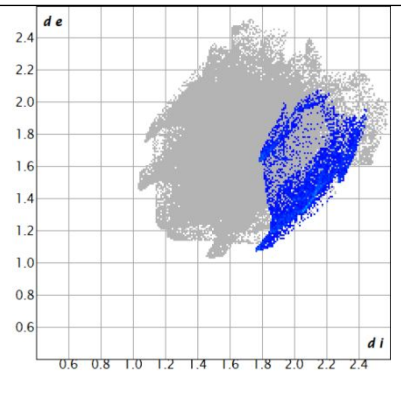

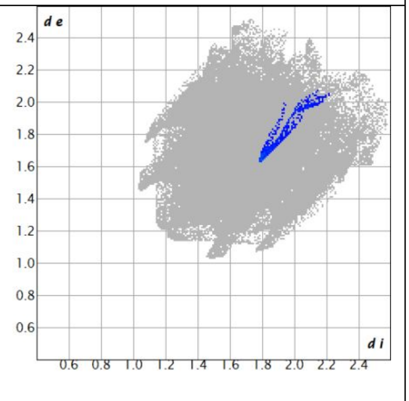

C - H	S - All	S – C
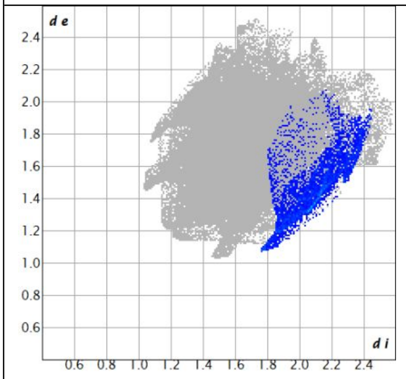	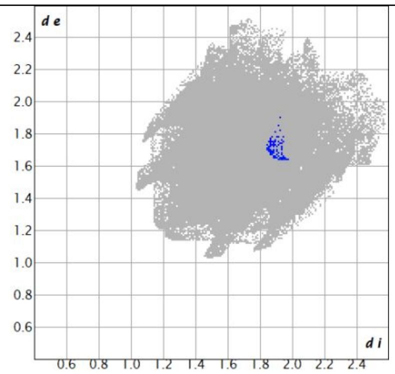	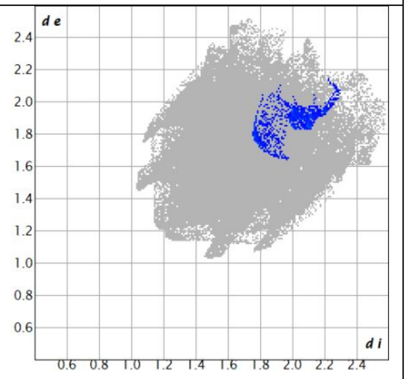
S - H	S-N	C – N
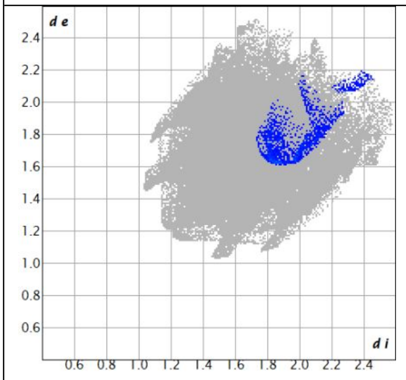	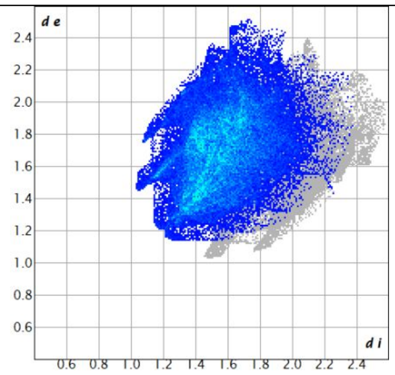	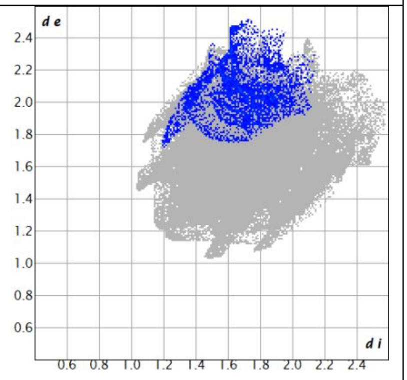
C - O	H- All	H – C
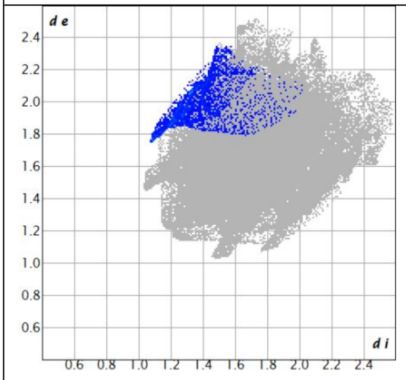	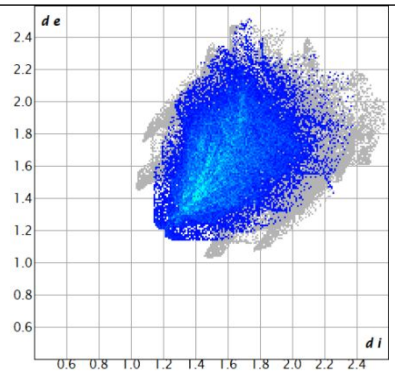	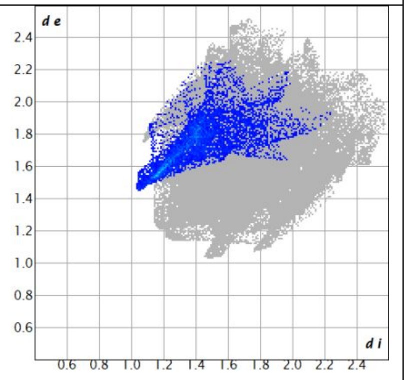
H- S	H - H	H – N
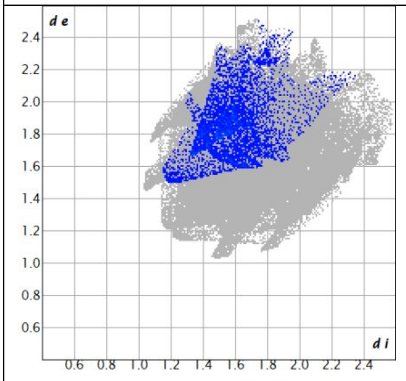	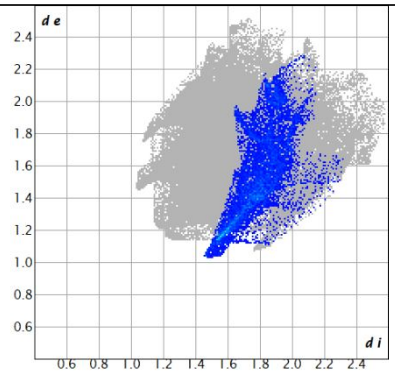	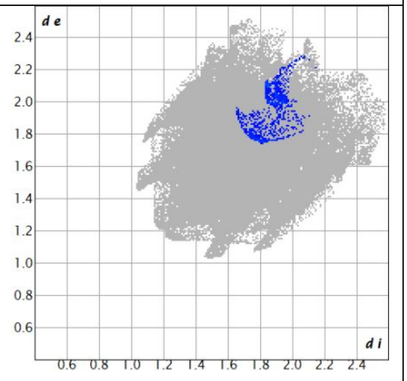

H-O	N- All	N – C

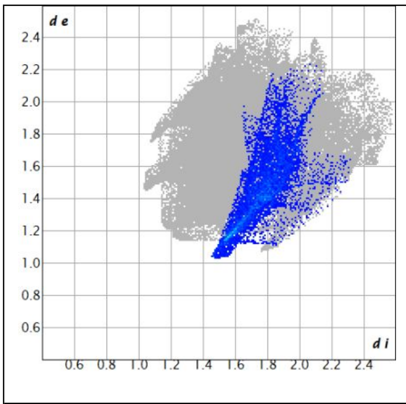	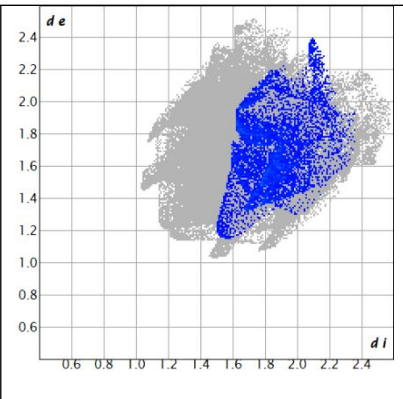	
N-H	O-all	
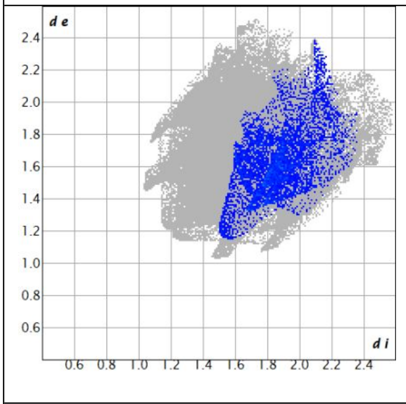	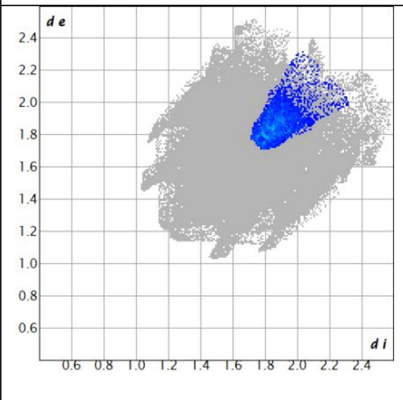	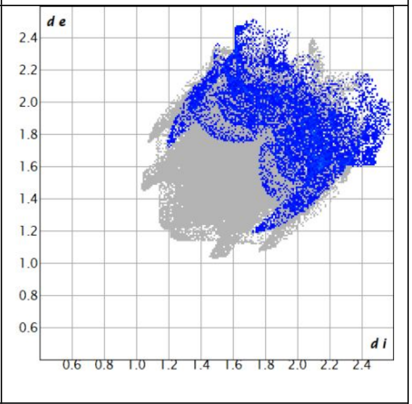
O-H	C – C reciprocal	C – H reciprocal
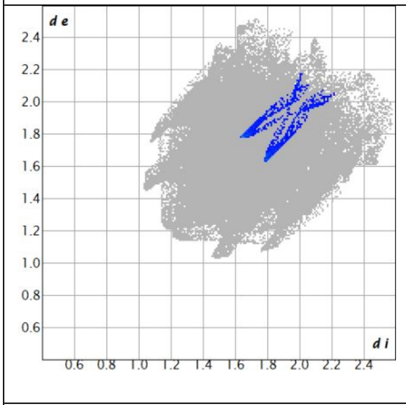	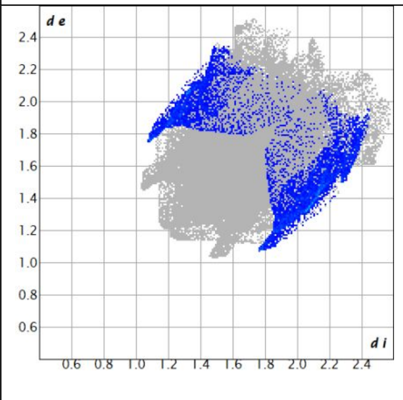	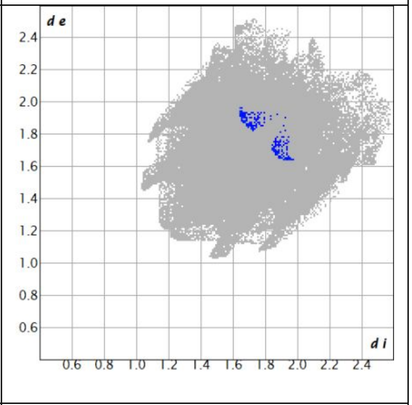
S – C reciprocal	S – H reciprocal	S-N reciprocal

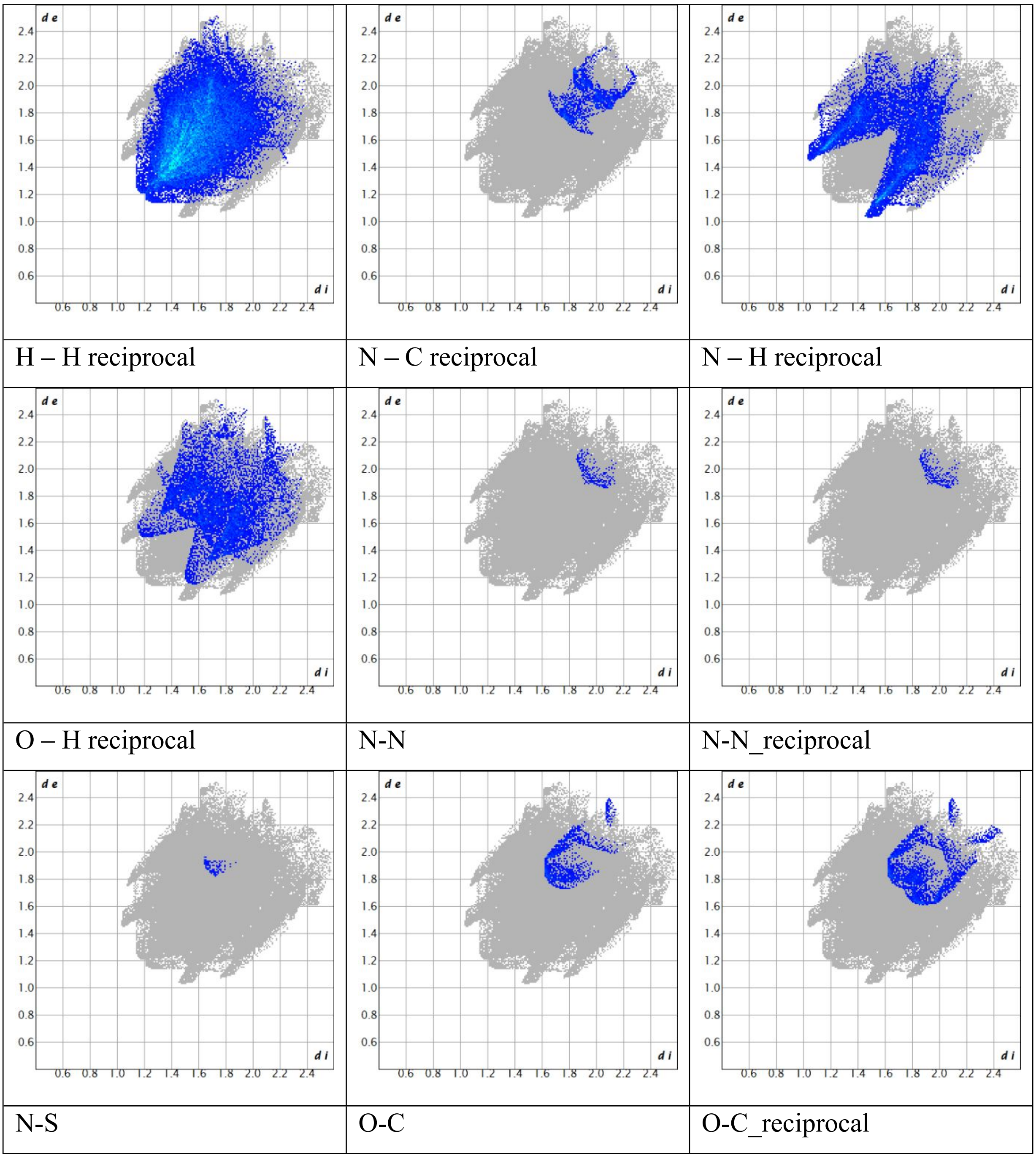

Figure S56: 2D fingerprint plot

Table S9: Fingerprint data

**D_i – D_e**	**Surface area included (%)**
All-All	100
All-C	12.3
All-O	6.6
All-N	8.3
All-S	3.1

All-H	69.6
S-All	5.1
O-All	0.4
N-All	9.6
C-All	14.5
H-All	63.4
S-S	0.0
S-O	0.0
S-N	0.1
S-C	0.6
S-H	4.4
O-S	0.0
O-O	0.0
O-C	1.4
O-H	6.0
N-S	0.1
N-N	0.3
N-C	1.1
N-H	8.1
C-S	0.5
C-O	1.6
C-N	1.3
C-C	4.9
C-H	6.1
H-S	2.5
H-O	5.0
H-N	6.6
H-C	4.3
H-H	45.0
S-N_reciprocal	0.3
S-C_reciprocal	1.1
S-H_reciprocal	6.9

O-C_reciprocal	3.0
O-H_reciprocal	11.0
N-N_reciprocal	0.3
N-C_reciprocal	2.4
N-H_reciprocal	14.8
C-C_reciprocal	4.9
C-H_reciprocal	10.4
H-H_reciprocal	45.0

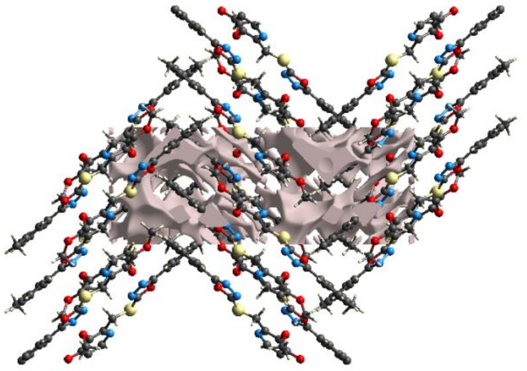	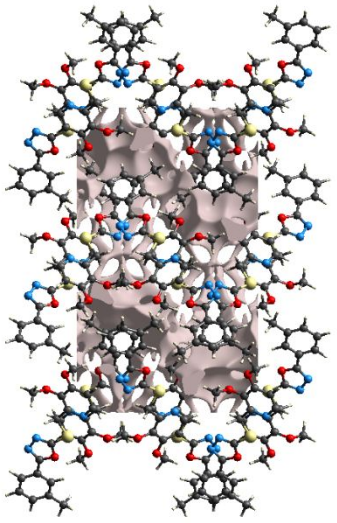
a-axis	b-axis
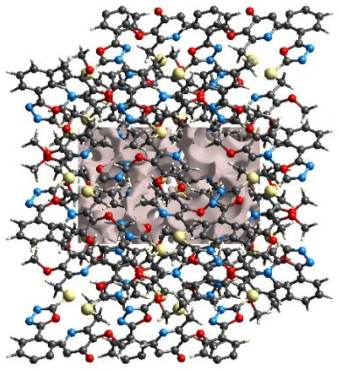	
C-axis	

Figure S57: Void analysis

Table S10: Void parameters

Void parameters	
Volume	354.45 Å^3

Area	1264.39 Å^2
Globularity	0.192
Asphericity	0.248
Crystal Parameter	
Formula	$C_{17}H_{17}N_3O_3S$
Spacegroup	p b c a
a	14.583
b	9.3773
c	24.386
alpha	90
beta	90
gamma	90
% of void	
Void volume * 100/(a*b*c)	10.63%

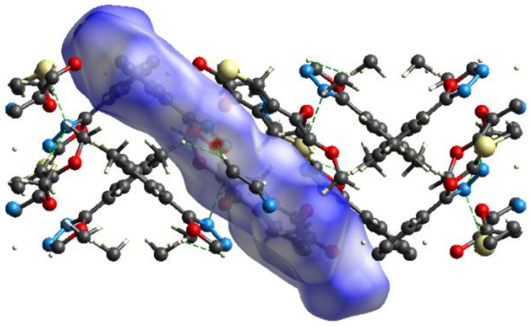	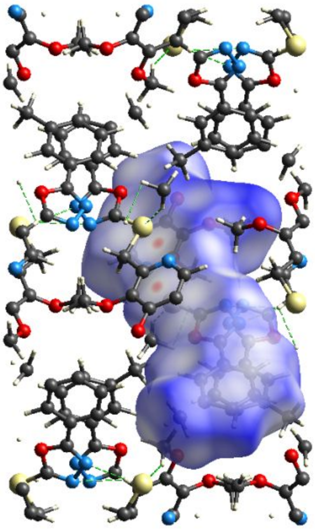
Along a axis	Along b axis
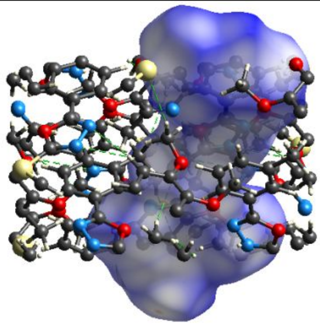	
Along c axis	

Figure S58: interaction of molecule with neighboring molecules in unit cell

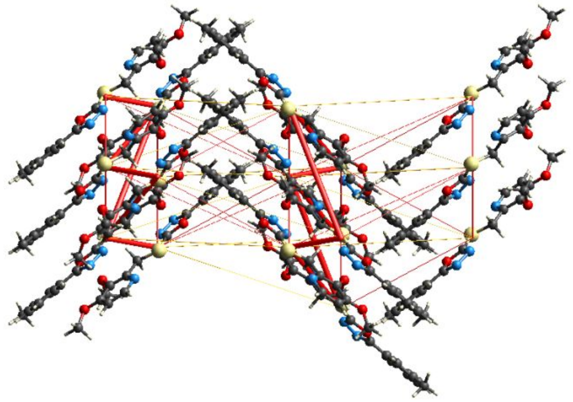

Coulomb energy (along a axis)

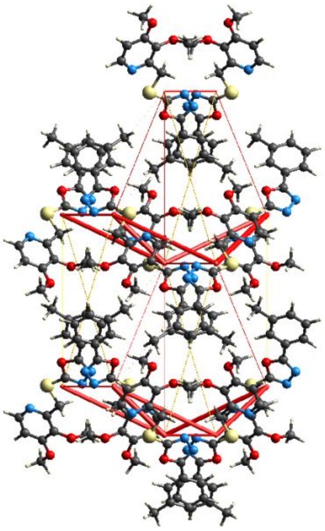

Coulomb energy (along b axis)

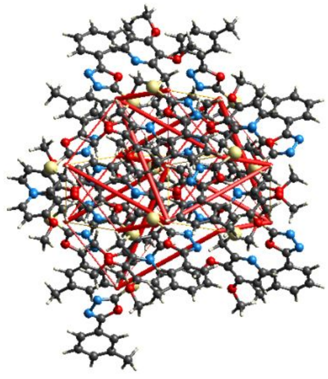

Coulomb energy (along c axis)

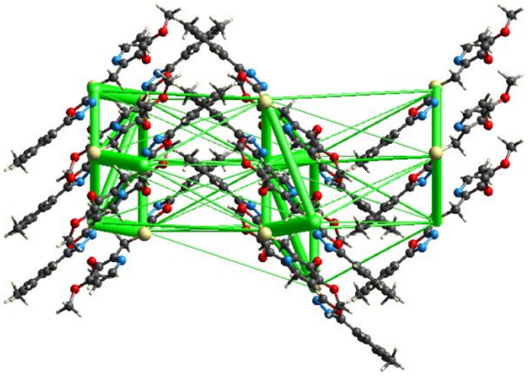

Dispersion energy (along a axis)

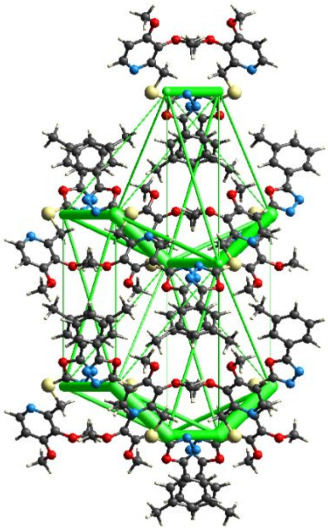

Dispersion energy (along b axis)

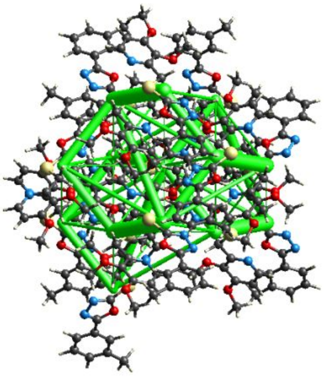

Dispersion energy (along c axis)

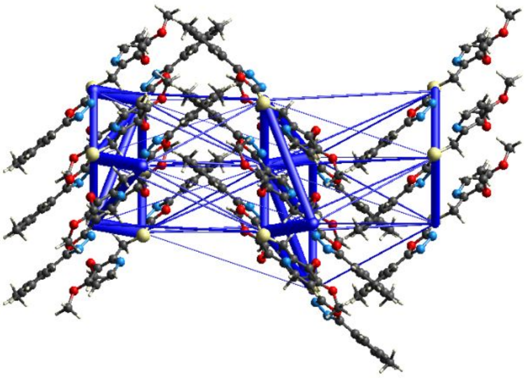
Total energy (along a axis)
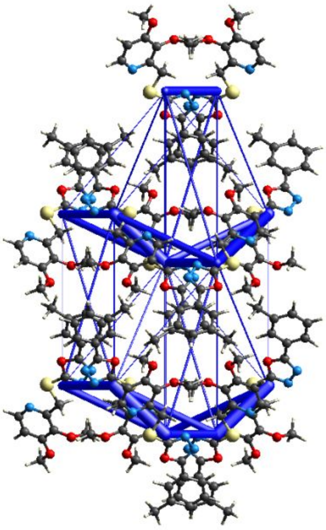
Total energy (along b axis)
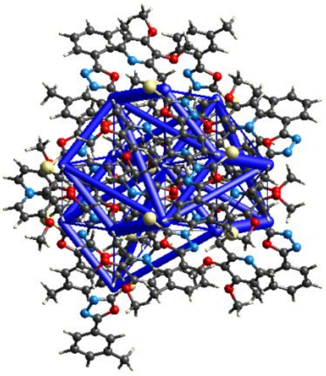
Total energy (along c axis)

Figure S59: Energy framework

Table S11: Interaction energies (kJ/mol) calculated using B3LYP/6-31G(d,p)

Interaction Energies (kJ/mol)
R is the distance between molecular centroids (mean atomic position) in Å.

Total energies, only reported for two benchmarked energy models, are the sum of the four energy components, scaled appropriately (see the scale factor table below)

	N	Symop	R	Electron Density	E_ele	E_pol	E_dis	E_rep	E_tot
	1	-x, -y, -z	10.11	B3LYP/6-31G(d,p)	-23.7	-5.2	-50.2	47.3	-43.4
	2	-x+1/2, y+1/2, z	6.01	B3LYP/6-31G(d,p)	-7.2	-5.0	-50.2	24.3	-40.1
	2	x+1/2, -y+1/2, -z	9.05	B3LYP/6-31G(d,p)	-19.1	-6.2	-22.9	16.1	-34.8
	2	-x+1/2, -y, z+1/2	12.77	B3LYP/6-31G(d,p)	3.3	-0.9	-15.5	0.0	-10.8
	2	x, -y+1/2, z+1/2	12.89	B3LYP/6-31G(d,p)	1.9	-0.7	-6.5	0.0	-4.1
	1	-x, -y, -z	4.90	B3LYP/6-31G(d,p)	-20.6	-4.6	-78.9	58.3	-57.8
	2	-x, y+1/2, -z+1/2	10.61	B3LYP/6-31G(d,p)	-2.6	-0.4	-11.1	7.7	-8.0
	2	x+1/2, y, -z+1/2	11.46	B3LYP/6-31G(d,p)	0.3	-0.2	-7.0	2.7	-4.2
	2	x, -y+1/2, z+1/2	13.26	B3LYP/6-31G(d,p)	-2.3	-0.5	-6.0	0.0	-8.0

Scale factors for benchmarked energy models
See Mackenzie et al. IUCrJ (2017)

Energy Model	k_ele	k_pol	k_disp	k_rep
CE-HF ... HF/3-21G electron densities	1.019	0.651	0.901	0.811
CE-B3LYP ... B3LYP/6-31G(d,p) electron densities	1.057	0.740	0.871	0.618

5]Molecular Docking

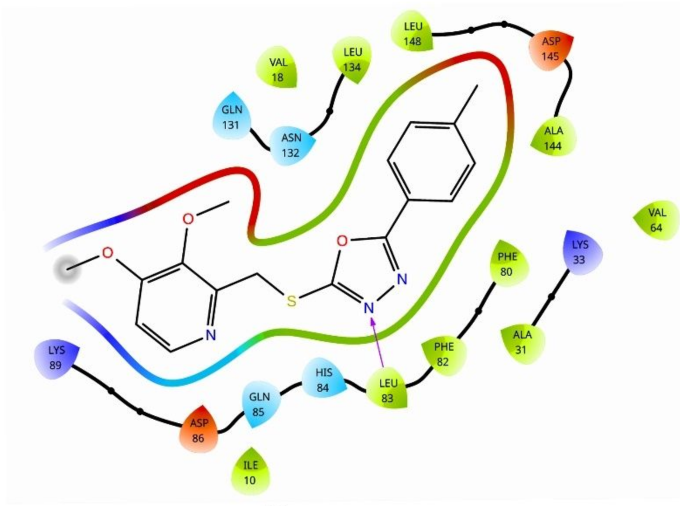

5a

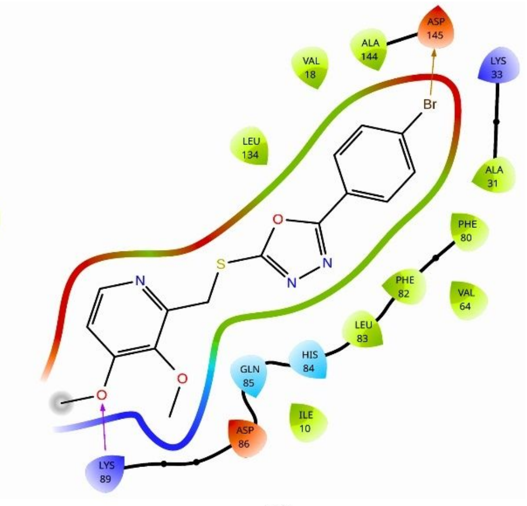

5b

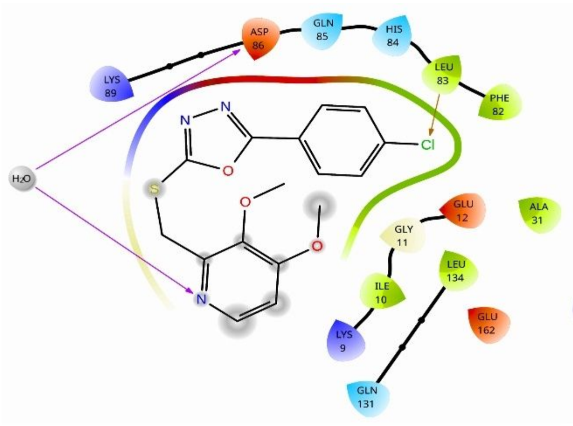

5c

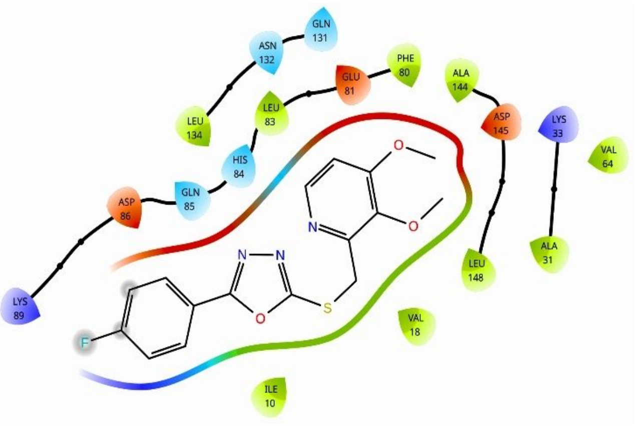

5d

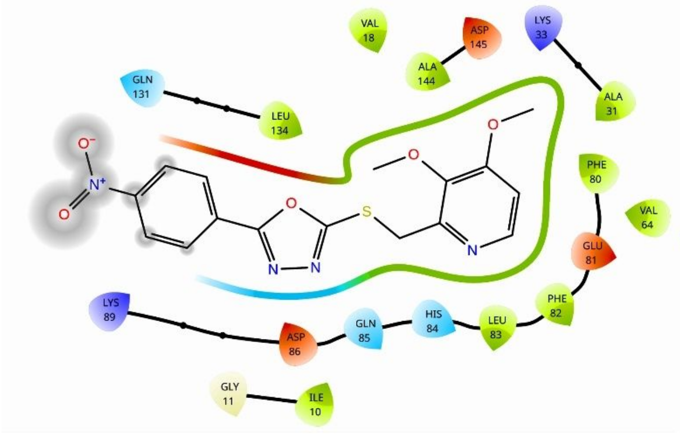

5e

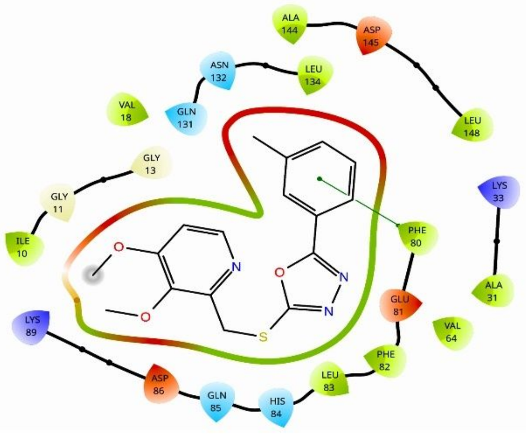

5f

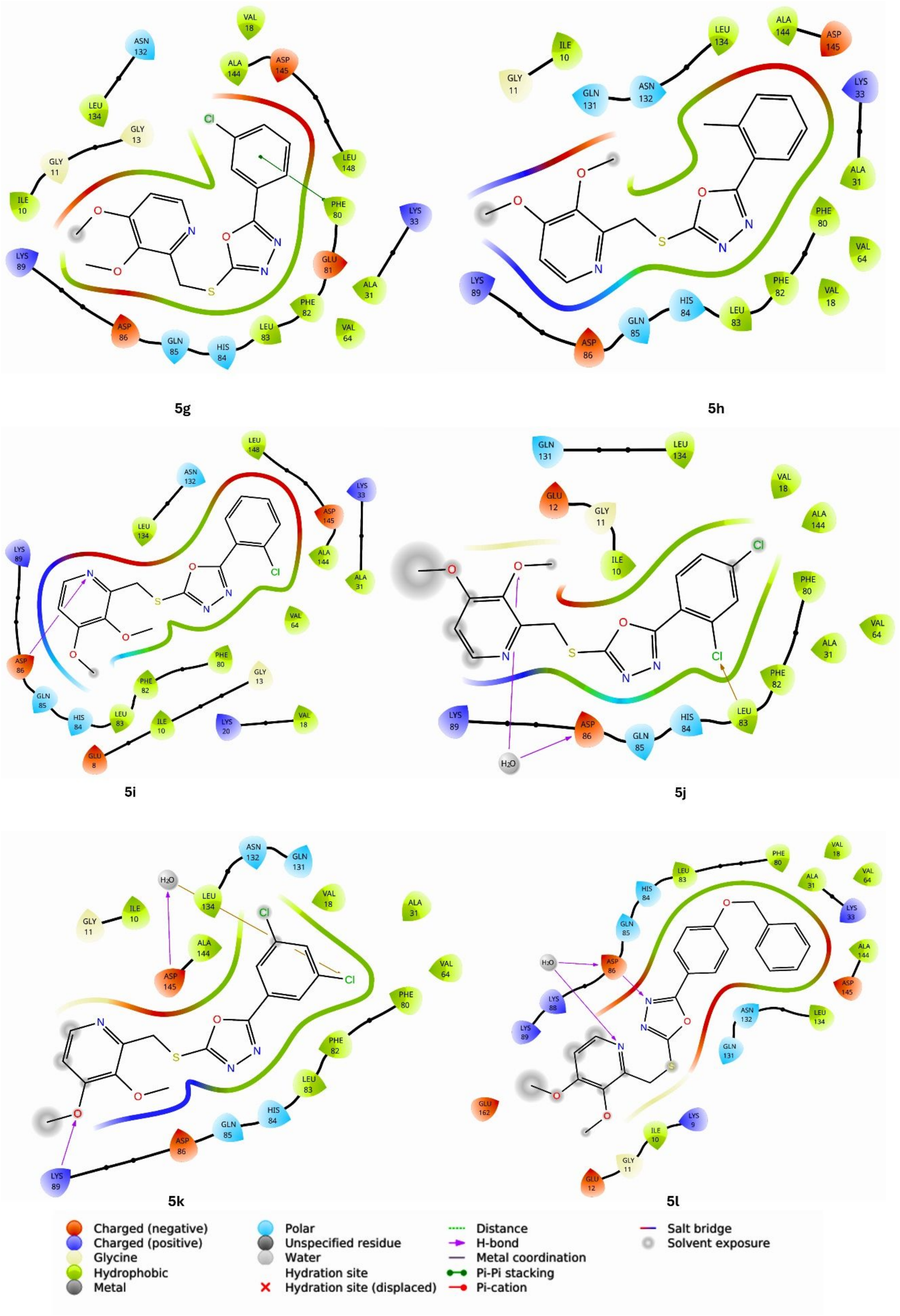

Figure S60: Molecular interactions of pyridine-based 1,3,4-oxadiazole hybrids (**5a–l**) at the binding site of Cyclin-Dependent Kinase 2 (CDK2). Interaction types are color-coded as

follows: hydrogen bonds (magenta arrows), π–π stacking interactions (green lines), Halogen bonds (tangerine arrow). Residues are classified by chemical nature: charged (red for negative, blue for positive), polar (sky blue), hydrophobic (green) and glycine (light yellow). Water molecules and hydration sites are also indicated

Table S12: Summary of key interactions between synthesized ligands and binding pocket residues of CDK2

**Ligand**	**Key interactions**
**5a**	H-bond (Leu83), Hydrophobic (Leu83, Phe82, Phe80, Leu134)
**5b**	H-bond (Lys89), Halogen bond (Asp145), Hydrophobic (Leu83, Phe82, Phe80, Leu134, Val18)
**5c**	Water mediated H-bond (Asp86), Halogen bond (Leu83), Hydrophobic (Leu83, Phe82)
**5d**	Hydrophobic (Val18, Leu148, Ile10)
**5e**	Hydrophobic (Leu83, Phe82, Phe80, Ala144, Ala31)
**5f**	π–π interactions (Phe80), Hydrophobic (Leu83, Phe82, Phe80)
**5g**	π–π interactions (Phe80), Hydrophobic (Leu83, Phe82, Phe80)
**5h**	Hydrophobic (Leu83, Phe82, Phe80, Ala31)
**5i**	H-bond (Asp86), Hydrophobic (Phe80, Val64)
**5j**	Water mediated H-bond (Asp86), Halogen bond (Leu83), Hydrophobic (Leu83, Phe82, Phe80, Ile10)
**5k**	H-bond (Lys89), Water mediated polar interaction (Asp145), Hydrophobic (Leu83, Phe82, Phe80, Val18, Ala144)
**5l**	Water mediated H-bond (Asp86), H-bond (Asp86), Hydrophobic (Leu83, Phe80, Ala31, Leu134)

6] Molecular Dynamics

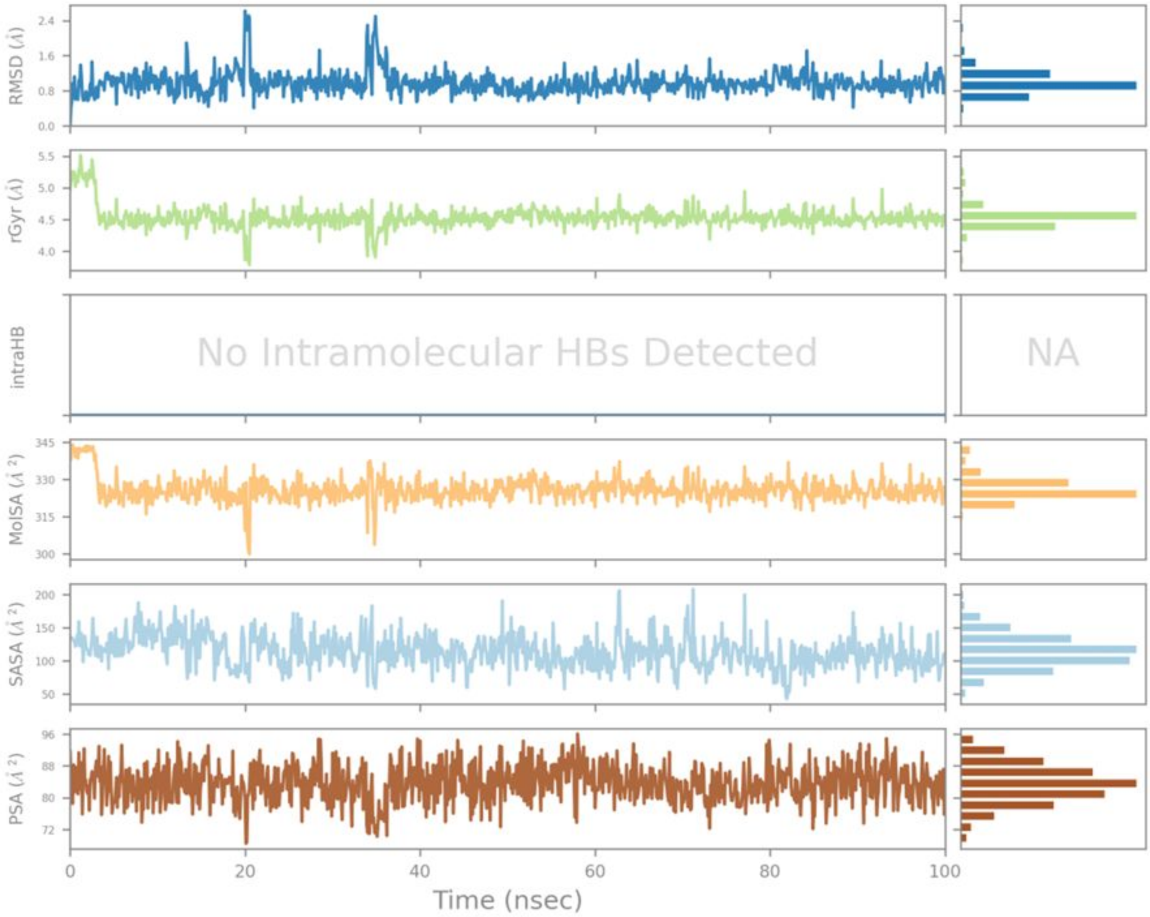

Figure S61: Time evolution of structural parameters during molecular dynamics simulations: radius of gyration (Rg), polar surface area (PSA), molecular surface area (MolSA), and solvent-accessible surface area (SASA)

References

(1) Kumar, A. C.; Rangaswamy, J.; Madalambika; BharathKumar, P. M.; Patil, P. R.; Salavadi, M.; Naik, N. Novel 1,3,4-Oxadiazole-2- Thiol Derivatives: Unlocking the Therapeutic Potential as Anti-Inflammatory and Anticancer Agents. *J. Mol. Struct.* **2024**, *1315*, 138749. https://doi.org/10.1016/J.MOLSTRUC.2024.138749.

(2) El-Zahabi, M. A.; Sakr, H.; El-Adl, K.; Zayed, M.; Abdelraheem, A. S.; Eissa, S. I.; Elkady, H.; Eissa, I. H. Design, Synthesis, and Biological Evaluation of New Challenging Thalidomide Analogs as Potential Anticancer Immunomodulatory Agents. *Bioorg. Chem.* **2020**, *104*, 104218. https://doi.org/10.1016/J.BIOORG.2020.104218.

(3) Khan, A.; Elhenawy, A. A.; Rehman, M. U.; Alam, M.; Alam, A.; Rehman, N. U.;

Ibrahim, M. Synthesis of Novel 2-Mercapto-1,3,4-Oxadiazole Derivatives as Potent Urease Inhibitors: In Vitro and in Silico Investigations. *J. Mol. Struct.* **2024**, *1312*, 138596. https://doi.org/10.1016/J.MOLSTRUC.2024.138596.
